# Strategy for improved characterization of human metabolic phenotypes using a COmbined Multi-block Principal components Analysis with Statistical Spectroscopy (COMPASS)

**DOI:** 10.1093/bioinformatics/btaa649

**Published:** 2020-07-21

**Authors:** Ruey Leng Loo, Queenie Chan, Henrik Antti, Jia V Li, H Ashrafian, Paul Elliott, Jeremiah Stamler, Jeremy K Nicholson, Elaine Holmes, Julien Wist

**Affiliations:** Centre for Computational and Systems Medicine, Perth, WA 6150, Australia; The Australian National Phenome Centre, Health Futures Institute, Murdoch University, Perth, WA 6150, Australia; Department of Epidemiology and Biostatistics, London W2 1PG, UK; MRC Centre for Environment and Health, School of Public Health, Imperial College London, London W2 1PG, UK; Department of Chemistry, Umea Universitet, 901 87 Umeå, Sweden; Department of Surgery and Cancer, Imperial College London, London W2 1PG, UK; Department of Surgery and Cancer, Imperial College London, London W2 1PG, UK; Department of Epidemiology and Biostatistics, London W2 1PG, UK; MRC Centre for Environment and Health, School of Public Health, Imperial College London, London W2 1PG, UK; Department of Preventive Medicine, Feinberg School of Medicine, Northwestern University, Chicago, IL, USA; Centre for Computational and Systems Medicine, Perth, WA 6150, Australia; The Australian National Phenome Centre, Health Futures Institute, Murdoch University, Perth, WA 6150, Australia; Centre for Computational and Systems Medicine, Perth, WA 6150, Australia; The Australian National Phenome Centre, Health Futures Institute, Murdoch University, Perth, WA 6150, Australia; Department of Surgery and Cancer, Imperial College London, London W2 1PG, UK; Chemistry Department, Universidad del Valle, Cali, Colombia

## Abstract

**Motivation:**

Large-scale population omics data can provide insight into associations between gene–environment interactions and disease. However, existing dimension reduction modelling techniques are often inefficient for extracting detailed information from these complex datasets.

**Results:**

Here, we present an interactive software pipeline for exploratory analyses of population-based nuclear magnetic resonance spectral data using a COmbined Multi-block Principal components Analysis with Statistical Spectroscopy (COMPASS) within the R-library hastaLaVista framework. Principal component analysis models are generated for a sequential series of spectral regions (blocks) to provide more granular detail defining sub-populations within the dataset. Molecular identification of key differentiating signals is subsequently achieved by implementing Statistical TOtal Correlation SpectroscopY on the full spectral data to define feature patterns. Finally, the distributions of cross-correlation of the reference patterns across the spectral dataset are used to provide population statistics for identifying underlying features arising from drug intake, latent diseases and diet. The COMPASS method thus provides an efficient semi-automated approach for screening population datasets.

**Availability and implementation:**

Source code is available at https://github.com/cheminfo/COMPASS.

**Supplementary information:**

Supplementary data are available at *Bioinformatics* online.

## 1 Introduction

Systems biology approaches using multi-omic platforms can inform on biological pathways and mechanisms underlying disease risk and identify potential targets for new treatments or preventive measures (Elliott *et al.*, 2015; Holmes *et al.*, 2008). Metabolic profiling (‘metabolomics/metabonomics’), using mass spectrometric (MS) and nuclear magnetic resonance (NMR) spectroscopic platforms, is increasingly being used to generate multi-parametric metabolic data for understanding biological systems. A multitude of informatic tools have been used to model and interpret complex metabolic profiling datasets generated by these analytical platforms. Principal component analysis (PCA) (Jackson, 1991; Wold *et al.*, 1987) and partial least squares projections to latent structures (PLS) (Tennenhaus, 1998; Wold *et al.*, 2001a,b) are two of the most widely implemented statistical tools on the basis of their data compression ability, visualization properties, high interpretability, robustness and inherent transparency with respect to feature (metabolite) weighting, compared to other methods. These linear projection methods and other multivariate statistic methods aim to reduce data dimensionality by summarising the main variance in the data in two or three components. However, such methods become inefficient as the number of variables increases to the order of thousands, as is the case with most 'omics studies (Wold *et al.*, 2002).

Application of metabolic profiling and multivariate statistics to human population studies has the added challenge of dealing with large numbers of samples as well as variables and many important biological phenomena in these studies are masked by the main sources of variance in the dataset, which may not be relevant to the biological focus of the study. Therefore, the challenge lies in finding a robust strategy to extract and interpret relevant information from the dataset with high fidelity and reproducibility. A common approach is to use variable selection prior to modelling. However, this increases the risk of misinterpretation and can result in loss of information and poor model robustness. To avoid this, the data variables and/or samples can be divided into regions (blocks) and subsequently analysed in a hierarchical fashion, e.g. Quilt-PCA or -PLS (Westerhuis *et al.*, 1998; Wold *et al.*, 2002). Here, we extend this concept by combining a multi-block approach with statistical spectroscopy to provide a toolbox that focuses on stratified analysis of the data variables to facilitate recovery of metabolic features that would otherwise be dominated by the one or two major sources of variance (usually high concentration compounds) in the dataset. Sub-dividing the spectra into blocks allows key signals from each block that relate to sources of variance in the data such as presence of latent disease, drug use or dietary behaviours to be more easily identified. However, since molecules generally have signals in different regions of the NMR spectrum using the block data approach would reduce the information available for structural identification and hence, having identified key ‘biomarker’ signals in the blocked data we revert to the whole spectrum to compute correlations from the key signals in each block across the full spectral range to extract the total signal pattern for metabolites of interest. Using these signal patterns as a reference to define a cross-correlation (CC) cut-off point, a semi-automated system for identification of particular drug metabolites, dietary behaviours or pathologies can be built. The whole pipeline, referred to as COmbined Multi-block Principal components Analysis with Statistical Spectroscopy (COMPASS), was developed within the open source R environment and the hastaLaVista framework (Wist, 2019). We demonstrate the functionality of COMPASS using an exemplary dataset, the International Study of Macro/Micronutrients and Blood Pressure (INTERMAP) Study, a multi-country, cross-sectional population study on diet and blood pressure. The approach presented offers a rapid and efficient framework for preliminary screening various features within the INTERMAP dataset.

## 2 Materials and methods

### 2.1 Implementation

Supplementary Material S1 describes the COMPASS pipeline which utilizes two R-scripts: multi-blocking. R (Supplementary Material S2) and MBCC-metaboliteX.Rmd (Supplementary Materials S3) and two json files: (modelExplorer.view.json: Supplementary Material S4) and crossCorrelationExplorer.view.json (Supplementary Material S5). These scripts utilize the R-packages MetaboMate (code by Kimhofer: https://github.com/kimsche/MetaboMate) for multivariate statistics and hastaLaVista (https://github.com/jwist/hastaLaVista; Wist, 2019) for enhanced graphical and interactive visualization. The output is formatted as a pdf or docx format.

### 2.2 Usage of the COMPASS approach

**Fig. 1.**
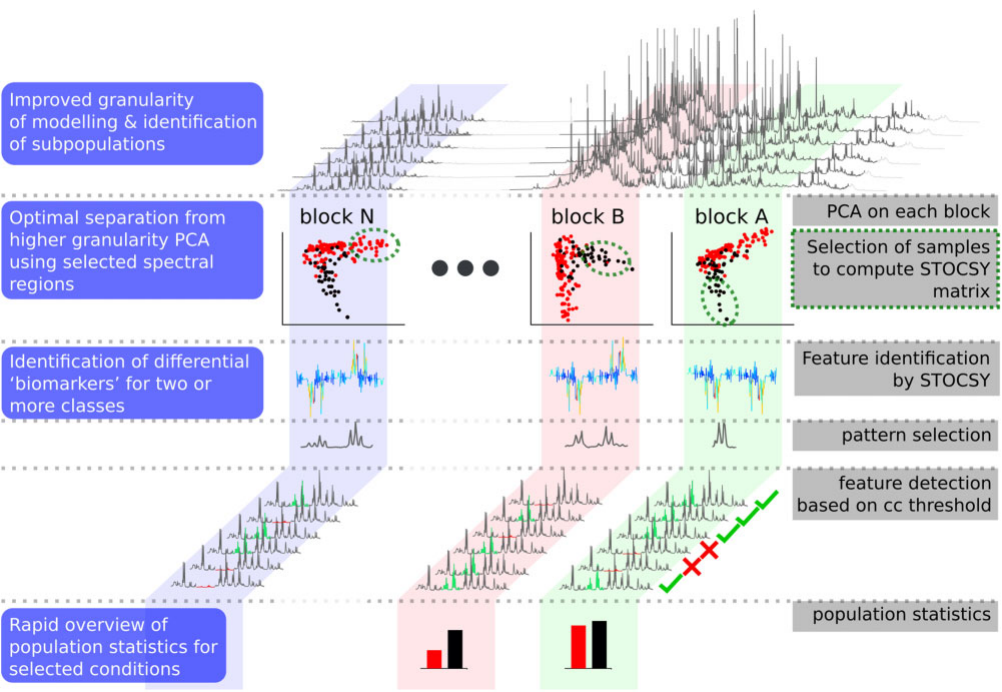
Schematic of the COMPASS analysis pipeline. This involves (i) sub-dividing the spectra into regions (blocks) to allow extraction of finer structure using the PCA scores and loading plots without strong signals in other regions of the spectrum dominating the variance; (ii) the user can then select spectra relating to sub-clusters of interest for computing the STOCSY from the individual multi-block-PCA scores plots, as illustrated using the green ellipses (arbitrary user defined subset based on inherent structure in the data). Selecting spectral data that are clustered together in the PCA scores plot increases the likelihood that the corresponding spectra contain the same feature of interest thereby reducing statistical noise in identifying reference patterns via STOCSY analysis; (iii) a reference exhibiting the reference patterns identified *via* the STOCSY is used to calculate a CC for each spectrum in the dataset. The CC distribution is then used as a guideline for choosing a suitable CC threshold for a particular metabolite feature; (iv) based on the CC distribution. The population statistics can be calculated for the compound/feature of interest and spectra displaying this feature can be presented in any format allowable in R (here, we use PDF or docx)

A schematic of the analysis workflow (Fig. 1) outlines the four-step goal:

1. to calculate individual PCA models for each spectral region (illustrated for blocks of 0.5 ppm width) to extract the finer structure in the spectral data relating to metabolic features associated with sources of variance in the dataset;
2. to obtain a reference pattern for identified features of interest by applying Statistical TOtal Correlation SpectroscopY (STOCSY) (Cloarec *et al.*, 2005), involving the computation of correlation matrices between the intensity of all composite points in the spectral data for a subset of samples containing the specific feature(s) of interest to provide additional molecular structural information and thereby allowing the visualization of a wider range of features;
3. to compute the distribution of the CC of any given pattern of interest (e.g. signal from a specific drug) and estimate the optimal CC value, which can be interactively adjusted by the user, thereby providing a means for screening the NMR spectral dataset for specific metabolites of interest. This can be simply visualized using a traffic light display system, i.e. green for spectra with high confidence in the presence of the signal pattern, amber for intermediate and red for those deemed not to contain the feature;
4. to generate a report in pdf or docx format based on the user-defined threshold CC value, which provides an estimation of the number of samples in the dataset containing a specific signal pattern (metabolite). The COMPASS pipeline, therefore, provides users with an interactive tool for rapid screening of different metabolic features of interest associated with, e.g. drug, alcohol, inborn errors of metabolism and dietary intake.

### 2.3 Description of the INTERMAP study

The INTERMAP study surveyed 4680 men and women 40–59 years of age from Japan, People’s Republic of China (PRC), United Kingdom (UK) and United States (US) in 1996–1999 (Stamler *et al.*, 2003). Participants were selected randomly from community or workplace population lists, arrayed into four age/sex strata. Each participant attended four clinic visits, two on consecutive days and two further visits on consecutive days on average 3 weeks later. Of the 4895 recruited into the study, participants were excluded if they failed to attend all four clinic visits (*n* = 110) or had incomplete/missing data or 24-h urine sample (*n* = 61), presented unreliable dietary data (*n* = 7) or had extreme total energy intake (>5000 kcal/day for women and >8000 kcal/day for men) from any 24-h recall (*n* = 37); giving a final 4680 participants in the study. Institutional ethics committee approval was obtained for each site; all participants provided written informed consent. The INTERMAP study was registered as NCT00005271 at https://clinicaltrials.gov.

### 2.4 ^1^H NMR spectroscopic analysis

Each participant provided two borate-preserved timed 24-h urine collections; aliquots of urine were frozen on site (-20°C) and air-freighted frozen to the Central Laboratory (Leuven, Belgium) for biochemical analyses. Urine specimens were thawed completely before mixing 500 µL of urine with 250 µL of phosphate buffer (0.2 M) for the stabilization of urinary pH 7.4 (±0.5), and 75 µL of sodium 3-trimethylsilyl-(2,2,3,3-d)-1-propionate (TSP) in deuterium oxide (D_2_O, final concentration 0.1 mg/mL) solution for chemical shift referencing of TSP (d 0.0). ^1^H NMR spectra of the urine specimens were obtained at 300 K using a Bruker (Bruker Biospin, Rheinstetten, Germany) Avance 600 spectrometer at the operating ^1^H frequency of 600.13 MHz. A standard one-dimensional pulse sequence (recycle delay—90°—t_1_—90°—tm—90° acquisition) with a water pre-saturation was used (McKay, 2011; Nicholson *et al.*, 1995), with a relaxation delay of 2 s and mixing time of 100 ms. About 64 free induction decays were collected into 32 K data points using a spectral width of 20 ppm (Loo *et al.*, 2009).

The ^1^H NMR spectra were processed by a standard protocol in which they were phased, referenced to TSP and underwent baseline correction. The region δ4.5–6.55 containing the residual water and urea resonances, and the regions at δ < 0.5 and >9.6 containing predominantly noise were excluded from analyses. Of the 4680 individuals, one urine specimen was lost, 50 ^1^H NMR urinary spectra were unusable due to poor water suppression or excessive glucose metabolites that led to baseline distortion and 15 spectra that did not meet the half-height line width for TSP, leaving a total of 4614 ^1^H NMR urinary spectra (*N* = 826 for China, *N* = 1138 for Japan, *N* = 496 for UK and *N* = 2154 for USA), available for testing the COMPASS workflow. Each spectral segment corresponded to a data point with a spectral width of δ 0.0005 and was normalized using a probabilistic quotient method (Dieterle *et al.*, 2006).

### 2.5 Application of COMPASS using INTERMAP study

Here, we exemplify the functionality of COMPASS using the INTERMAP dataset where NMR urine spectra were obtained for 4614 individuals from four countries (U.S.A., U.K., Japan & China) with differing cardiovascular disease risks. We also provide the reader with a less complex dataset from an animal surgical dataset (Li *et al.*, 2011a, 2011b) for familiarization with the software. A brief description of the data is supplied in Supplementary Material S6 (note: no data interpretation was performed on this animal dataset. The dataset is merely provided to allow the exploration of the software capability as described in Supplementary Material S1).

## 3 Results

**Fig. 2.**
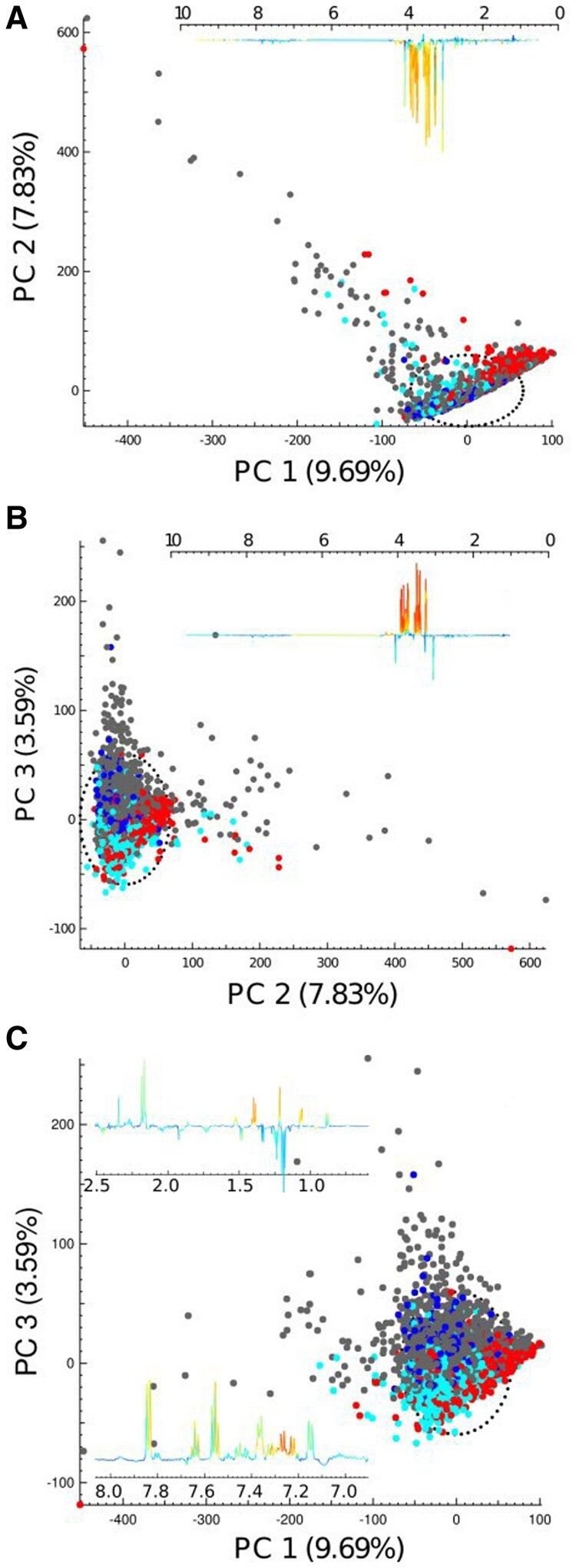
PCA model of the global (total spectrum) showing (**A**) scores plots for PC 1 and 2 and loading for PC 1 dominated by glucose metabolites; (**B**) scores plots for PC 2 and 3 and loading for PC 2 also dominated by glucose metabolites and (**C**) scores plots for PC 1 and 3 and loading for PC 3 dominated by ibuprofen and its related metabolites (in the region between 0.5 and 1.5 ppm), acetaminophen and its related metabolites (region around 2.13 ppm and between 7.1 and 7.4 ppm) and hippurate (region around 7.5–7.8 ppm). All PCA scores plots are colour coded to country: China (red), Japan (turquoise), UK (blue) and USA (grey)

The PCA model generated from the whole dataset showed that the first two PCs are dominated by variance in glucose concentration (Fig. 2A, B) and commonly used drug metabolites, ibuprofen and acetaminophen (PC3, Fig. 2C). Outliers with high concentrations of glucose and drug metabolites were also apparent. In contrast, multi-block-PCA analysis of spectral regions of 0.5 ppm width from 0.5 to 9.6 ppm (excluding the water signal) resulted in the creation of 14 blocks or PCA sub-models with a broader range of metabolites associated with specific coherent patterns in the data. For each multi-block-PCA, spectral data were scaled to unit-variance, although depending on the dataset it may be appropriated to use other scaling methods to maximize information recovery from each block. Additionally, users can examine the scores plots to understand the deeper structure of the data. The blocks can then be collectively co-analysed using the scores (typically using up to three principal components, depending on the *R*^2^*X* values of the model) as the new input variables to build a hierarchical ‘supermodel’ presenting the main features of the individual blocks, with each block given equal weighting thereby ensuring that the model is less influenced by ‘noisy’ high concentration signals that may only be present in one or two blocks.

**Fig. 3.**
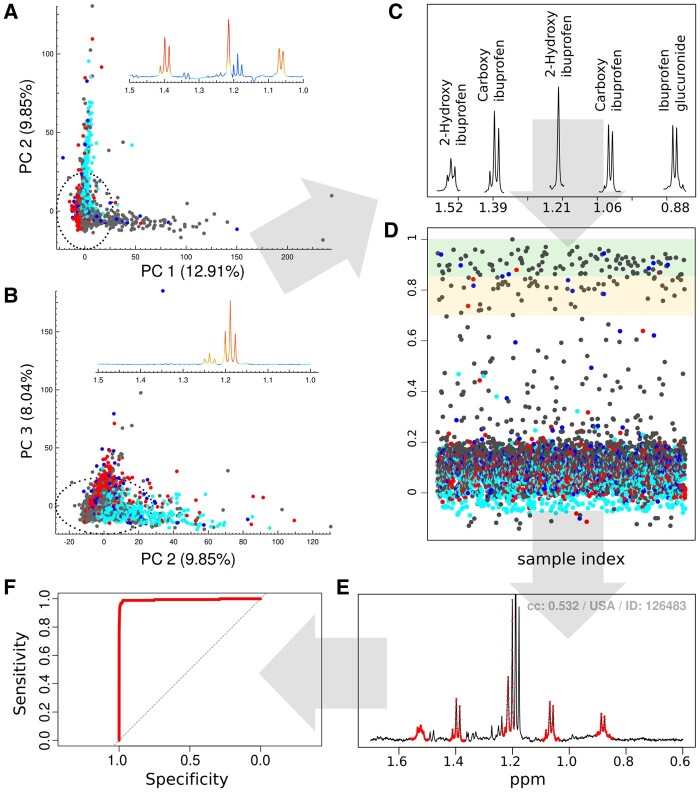
Multi-block-PCA for region 1–1.5 ppm showing: (**A**) scores plots for PC 1 and 2 and loading for PC 1 dominated by ibuprofen and its related metabolites; (**B**) scores plots for PC 2 and 3 and loading for PC 2 dominated by ethanol and its related metabolites, ethyl glucuronide and ethyl glycoside; (**C**) the robust pattern consists of a combination of major ibuprofen metabolites were obtained using STOCSY; (**D**) the distribution of CC for the robust ibuprofen pattern, as shown in C; area are highlighted by green panel corresponding to high confidence CC value whilst amber showing intermediate confidence CC value; (**E**) an exemplar NMR spectrum that show discrepancy between COMPASS and OPLSD-DA methods. This spectrum shows low CC value (0.532) due to low signal intensity of major ibuprofen metabolites with overlapping strong signals from ethanol at 1.2 ppm and (**F**) receiver-operating characteristic (ROC) curve comparing sensitivity and specificity of COMPASS against the results from the OPLSD-DA method. All PCA scores plots are colour coded according to country: China (red), Japan (turquoise), UK (blue) and USA (grey)

To illustrate our approach, we focus on known signals related to exogenous features (e.g. drug or alcohol metabolites) as well as latent diseases (e.g. diabetes reflected as glycosuria). The multi-block-PCA identifies many more signals of interest than the whole PCA model. For example, the first PC for the 1–1.5 ppm block is dominated by ibuprofen and its metabolites (Fig. 3A), whilst ethanol and its related metabolites (ethyl glucuronide; ethyl glycoside) drive the variance in PC2 (Fig. 3B). However, even in the scores plot of the 1–1.5 ppm block, the overlap of ∼5000 data points makes it difficult to distinguish the number of participants excreting ibuprofen. With the use of STOCSY, reference patterns relating to specific metabolites can be identified (Fig. 3C). Here, we increase the confidence in our assignment of ibuprofen based on the signals in the 1.0–1.5 ppm block by computing correlations across the entire spectrum to extract the total signal pattern for ibuprofen metabolites. We identified a representative spectrum exhibiting the ‘typical’ pattern providing a clear definition of the combination of major ibuprofen metabolites (signals) at 0.85–0.91, 1.03–1.09, 1.20–1.24, 1.37–1.42 and 1.50–1.56 ppm. Using this reference spectrum, we then calculated the distribution of the CC and colour coded the coordinates according to country (Fig. 3D). Computing the distribution of the CC of these signal patterns within the whole dataset allows the user to rank spectra according to the likelihood of correctly detecting the corresponding reference patterns and the result is displayed using a traffic-light display system, i.e. green for high CC values, where users are confident that the spectra contain the metabolic feature; amber for intermediate CC values, where users have intermediate confidence that the spectra contain the metabolic feature; and red for CC values deemed to indicate absence of the metabolic feature. As the tool is interactive, the user can focus on the amber category and adjust the CC threshold accordingly thereby minimizing the time spent on visually analysing the spectra in the either category where the predictions are robust.

Using ibuprofen as an exemplar, the computed CC plot (Fig. 3D) shows that the majority of the spectra are clustered at a CC of <0.20 with a further group clustered at CC > 0.7, with the remainder of the spectra being scattered in the range of 0.2–0.7. We first applied a relatively conservative CC cut-off value for detecting ibuprofen reference patterns (Fig. 3C): here >0.85 was considered to represent the high confidence threshold for spectra containing ibuprofen metabolites (green), 0.7–0.85 was considered as an intermediate confidence band for containing ibuprofen metabolites (amber) and <0.7 as the threshold below which there are no ibuprofen metabolites present in the spectrum (red). Using this threshold, the COMPASS approach identified 175 of 4614 spectra as containing ibuprofen metabolites with high or intermediate confidence (China *N* = 4; Japan *N* = 0; UK = 12 and USA =159). Visual examination of these spectra at CC > 0.7 confirmed that all of the selected urinary ^1^H NMR spectra indeed contained ibuprofen metabolites. This shows the false-positive rate (spectra classified as containing ibuprofen metabolites that did not contain ibuprofen metabolites as determined by visual inspection) for COMPASS approach is 0% when the threshold is set at a CC of ≥0.7. This result was comparable to our previously published supervised method using orthogonal PLS-discriminant analysis (OPLS-DA) modelling of pre-selected drug metabolite signals, which found 176 samples containing major ibuprofen metabolites, China *N* = 4 (0.5%); Japan *N* = 1; UK = 10 (2.0%) and USA = 161 (7.5%) (Loo *et al.*, 2012). We calculated the sensitivity and specificity of the COMPASS approach and found it to be highly accurate, ROC AUC = 0.99 (Fig. 3F). We compared the discrepancies between the two methods and found 17 out of 4614 samples identified by COMPASS as containing ibuprofen metabolites that were missed in the OPLS-DA analysis (Supplementary Fig. S7A). Conversely, 16 samples with CC value <0.7 were uniquely identified by the OPLS-DA method (Supplementary Fig. S7B). Visual examination of these 16 spectra showed that one spectrum with CC = 0.063 was unlikely to contain ibuprofen metabolites (decision based on two independent experienced NMR spectroscopists). Thus, based on a CC threshold of 0.7, the performance of COMPASS was similar to the OPLS-DA method whereby each method ‘under’ detected similar numbers of spectra as containing ibuprofen metabolites amounting to a false negative rate of <10% (of the 190 spectra visually confirmed to contain ibuprofen metabolites) for both methods. Specifically for the COMPASS method, under-detection of these spectra was mainly due to low signal intensity of major ibuprofen metabolites combined with overlapping of strong signals from the -CH_3_ resonance of ethanol at 1.2 ppm (Fig. 3E).

Within the COMPASS approach, the user can interactively adjust the CC threshold. For ibuprofen, we have shown an example of a CC threshold of 0.7 (Fig. 3D). For illustration, we relaxed the CC threshold to CC > 0.7 as high confidence and CC between 0.7 and 0.5 as intermediate confidence for spectra containing ibuprofen metabolites whilst CC <0.5 was classified as not containing ibuprofen metabolites. Based on this more lenient threshold, the COMPASS approach identified 201 of 4614 spectra as containing ibuprofen metabolites (China *N* = 5; Japan *N* = 0; UK = 15 and USA =181). Visual examination of these spectra with CC > 0.5 showed all urinary ^1^H NMR spectra did contain ibuprofen metabolites with the exception of one spectrum with CC = 0.638 that was difficult to confirm without further spectroscopic analysis. Thus the false-positive rate for COMPASS approach is 0.5% at a CC of 0.5. Using a lenient threshold of a CC of 0.5, the COMPASS approach under-detected eight spectra with no false positive, whereas OPLS-DA method under-detected 35 spectra, thus the performance of COMPASS was deemed to be superior to the OPLS-DA method.

We further illustrate this pipeline using a combination of the major acetaminophen metabolites as the reference pattern (Supplementary Fig. S8A). A clear separation of samples with and without major acetaminophen metabolites can be identified using a CC cut-off point of 0.75 (Supplementary Fig. 8B, C). For acetaminophen, we found the COMPASS approach slightly outperformed the OPLS-DA method: *N* = 359 (COMPASS) versus *N* = 324 (OPLS-DA), ROC curve AUC = 0.99 (Supplementary Fig. S8D). There were 56 samples that showed a discrepancy between the two methods wherein COMPASS identified these spectra to contain major acetaminophen metabolites (11 spectra were deemed high confidence based on a CC > 0.85 and the remaining 45 were deemed intermediate confidence with CC between 0.75 and 0.85) but where the OPLS-DA method failed to detect (Supplementary Fig. S8E). Visual inspection of these spectra showed that these spectra all contained resonances from acetaminophen metabolites (results verified by two independent and experienced NMR spectroscopists). In contrast, 19 samples were not identified by the COMPASS approach but were identified by the OPLS-DA method (Supplementary Fig. S8F). Visual examination of these 19 samples showed four spectra with a CC < 0.75 that did not contain visibly identifiable acetaminophen metabolites. Thus, the COMPASS approach under-detected a total of 15 (4.0%) of the spectra containing acetaminophen when compared to the supervised method with no false-positive results for the identification acetaminophen metabolites in the spectra, thus COMPASS outperformed the OPLS-DA method.

**Fig. 4.**
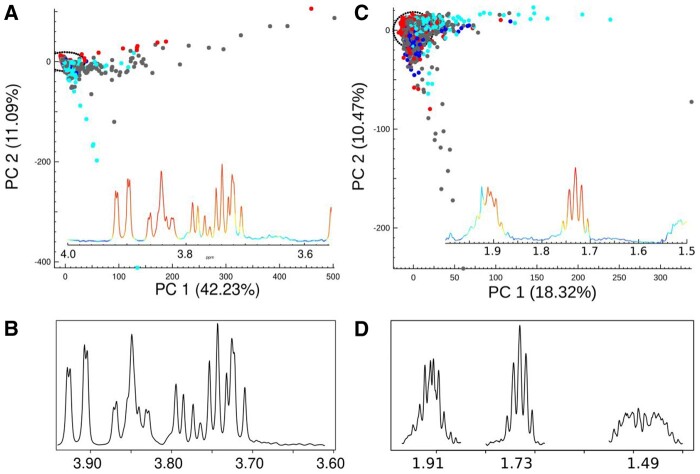
(**A**) Scores plot for PC 1 and 2, (**B**) loadings plot of the multi-block-PCA for region 3.5–4.0 ppm with PC 1 dominated by glucose metabolites, (**C**) scores plot for PC 1 and 2 and (**D**) loadings plot of the multi-block-PCA for region 1.5–2.0 ppm with PC 1 dominated by lysine metabolites. All PCA scores plots are colour coded according to country: China (red), Japan (turquoise), UK (blue) and USA (grey)

In addition to the detection of major sources of variance in the INTERMAP dataset (glucose, ibuprofen and acetaminophen), the multi-block-PCA approach enabled extraction of finer detail across the whole spectral region, independent of peak intensity. We illustrate this concept using population characteristics such as latent diseases (as illustrated with the presence of glycosuria and high levels of lysine in the urine, potentially reflecting diabetes and disorders of amino acid metabolism respectively; Figure 4A–D, Supplementary Materials S9 and S10); dietary habits [e.g. consumption of citrus fruits, alcohol and artificial sweeteners represented by proline betaine (Heinzmann *et al.*, 2010), ethanol (Teague *et al.*, 2004) and erythritol (Regnat *et al.*, 2018), respectively; Supplementary Materials S11–S13] and genetic factors [e.g. high urinary excretion of beta-aminoisobutyric acid (Yanai *et al.*, 1969), Supplementary Material S14]. Supplementary Material Files S9–S14 provide exemplars of the typical reports that can be obtained from COMPASS. Each report combines illustrations of the reference spectra for the metabolites of interest identified using STOCSY to determine the distribution of CC, together with randomly selected spectra from the high, intermediate and low confidence categories and a table of the calculated population statistics for each metabolite. The proportion of the population specimens manifesting spectral patterns relating to the aforementioned conditions, stratified by country, is provided in Table 1. For example, we detected 447 individuals of which 49.7% (*N* = 222) from Japan; 37.3% (*N* = 167) from the USA; 7.2% (*N* = 32) from China and 5.8% (*N* = 26) from the UK were identified to contain urinary levels of lysine above the set CC threshold (>0.6). We also observed that a higher proportion of Asian participants (50.2% in China and 40.9% in Japan) excreted detected levels of beta aminoisobutyric acid in their urine than those observed for the Caucasian population (21.8% in the UK and 25.6% in USA).

**Table 1.** Population statistics for various features identified by COMPASS approach

Feature	China	Japan	UK	USA	CC threshold
	*N* = 826 (%)	*N* = 1138 (%)	*N* = 496 (%)	*N* = 2154 (%)	
Ibuprofen	4 (0.5)	0	12 (2.4)	159 (7.4)	0.70
Ibuprofen	5 (0.6)	0	15 (3.0)	181(8.4)	0.50
Acetaminophen	36 (4.4)	24 (2.1)	65 (13.1)	234 (10.9)	0.75
Glucose	21 (2.5)	38 (3.3)	7 (1.4)	117 (5.4)	0.50
Lysine	32 (3.9)	222 (19.5)	26 (5.2)	167 (7.8)	0.60
Proline betaine	138 (16.7)	328 (28.8)	176 (35.5)	793 (36.8)	0.85
Ethanol	144 (17.4)	405 (35.6)	85 (17.1)	268 (12.4)	0.85
Erythritol	5 (0.6)	24 (2.1)	0	0	0.78
Beta isobutyric acid	415 (50.2)	465 (40.9)	108 (21.8)	552 (25.6)	0.90

## 4 Discussion

The COMPASS workflow provides a means of obtaining a rapid overview of the proportion of individuals within a population that excrete specific metabolites. The major advantage of the COMPASS approach is that it uses PCA to initially model data, which is an unsupervised method and allows agnostic exploration of the data, unlike discriminant analyses methods which use classification knowledge to obtain maximum differentiation between two or more classes and are thereby susceptible to overfitting. The COMPASS approach therefore allows identification of multiple features of interest that can be extracted interactively within the COMPASS approach without requiring pre-selection of a training set. This delivers additional biological knowledge on the dataset. Since the ‘PCA blocking’ approach investigates the dataset on a much finer level of detail compared to the ‘default’ whole dataset approach, phenomena such as latent disease, genetic disorders and consumption of drugs or specific foods are more easily detected by interpretation of the calculated sub-models, whilst the risk of misinterpretations is reduced since the model is no longer skewed by one or two high intensity variables in many cases. In contrast, the ‘default’ approach, in which the whole spectrum is normalized to the median spectrum, will always result in a model that is biased towards high intensity signals. Here, the COMPASS strategy allows a more efficient mapping of class-related patterns (in this case country), as well as detection of biomarkers that, differentiate the urinary metabolomes of the four countries measured, each with differing risks and prevalence of cardiovascular risk factors.

The incorporation of a traffic light display system based on the CC threshold enables the user to focus on the visual inspection of spectra from ambiguous cases (i.e. amber) rather than reviewing the entire dataset. The approach offers an efficient means for explaining deviation in anomalous samples and for identification of sub-populations or inherent structure within the data. The distribution of the CC across the dataset relating to selected metabolites, offers the user some guidance on the appropriate CC threshold for defining the presence and absence of a specific reference pattern. The user can further optimize the threshold, as this can be done interactively and iteratively with the COMPASS tools. Typically, we found the CC to be remarkably accurate in picking relevant reference patterns unless there was interference with other metabolites, which perturbed the pattern. However, the use of multiple features from a metabolite enabled a more robust CC value for the pattern, which is consequently less affected by peak overlap for any given signal, to be defined (as shown in the case of e.g. glucose and ibuprofen where the CC threshold can be set to as low as 0.5). Nonetheless, even for metabolites such as ethanol (which is illustrated based on a single triplet corresponding to the –CH_3_ resonance at 1.20 ppm) and proline betaine (which relies on two singlets at 3.11 and 3.30 ppm), the COMPASS approach remained effective in identifying spectra containing these features, although in these scenarios the CC threshold is set to a relatively conservative CC at >0.85 to limit the number of false negatives. The user may, if they choose, also define the ethanol pattern using both the –CH_3_ resonance at 1.20 ppm and the –CH_2_ quartet at 3.65 ppm. We assessed both and found that the use of the –CH_3_ resonance alone was superior in this case at the CC the threshold of 0.85 and this is mostly due to extensive peak overlapped for the –CH_2_ resonance.

The COMPASS approach is versatile and enables the user to define the size of each PCA block using either a uniform or non-uniform PCA block sizes. For the INTERMAP dataset, we used a uniform block size of 0.5 ppm for simplicity. Generally, we found that adjustment of either block size or start/end point for the PCA block to ensure metabolic features is not split between two PCA blocks (e.g. varying the block size to include the full multiplets of lysine at 1.47 ppm ( γ-CH_2_) and at 1.91 ppm ( β-CH_2_) within a single block) did not affect the overall results (data not shown). The low impact of a peak being ‘split’ between blocks in this case is most likely due to the fact that the data calculation of the reference pattern reverts back to using all the spectral data points for STOCSY rather than using the single block region. We also assessed the ability of the method to extract more metabolic features by reducing the PCA block size to half. We found PCA with smaller block sizes generally identified similar features as the larger block size at 0.5 ppm. However, for certain PCA blocks, additional key features may be identified e.g. for the multi-block PCA for 1–1.25 ppm, a singlet at 1.15 ppm (2-methyl-erythritol) which was predominately observed in the participants from China (results not shown) and likely reflects consumption of leafy vegetables (Smith *et al.*, 2007). Although in the current example, the size of the block had minimal impact when reducing from 0.5 to 0.25, we recommend that the selection of block size should be data driven since it is somewhat arbitrary. In selecting the block size, the user should consider the balance between subdividing spectral regions into enough blocks to minimize the effect of high variance, spectral resonances in particular regions dominating the model and creating blocks that are so small that multiple signals can be split or move between blocks. There is no reason all blocks should be of equal size and for regions that contain mainly noise with only a few signals then it would be logical to increase the block size. Another consideration at this point would be whether there is merit in applying different scaling to particular blocks or normalising within block. The optimal parameters will be dependent on the dataset and what the metabolic properties of that dataset contain, which is why the user interface is necessary rather than trying to achieve a fully automated pipeline.

The application of the COMPASS approach should have real-world benefit for clinicians including public health specialists and epidemiologists in augmenting rapid population metabolic profiling that could support public health campaigns both nationally and internationally. Here, we have demonstrated the feasibility of rapidly identifying disease patterns. For example, trends for glycosuria ranging from 2.5% in China to 5.4% in the USA which likely related to undiagnosed type 2 diabetes or renal disease. We also found the frequencies of 'high' beta-aminoisobutyric acid excretors are higher in China (50.2%) and Japan (40.9%) than the UK (21.8%) and USA (25.6%). This observation is consistent with literature, which suggests high excretion of beta-aminoisobutyric acid (>79.4 mg/mg creatinine) is an autosomal recessive trait more common in Chinese and Japanese population with a prevalence of 35–40% of the population considered to be ‘high’ excretors of beta-aminoisobutyric acid compared to around 10% in Caucasian populations (Yanai et al., 1969). We detected 447 individuals that showed urinary excretion of relatively high levels of lysine. The self-reported lysine intake per day, estimated based on consumption of both animal and vegetable protein intake including those from cereal protein, pulse-soy protein and amino acid supplementation, was found to be the highest for USA (6.23 g/day) and the lowest for China (3.85 g/day) whilst Japan (5.55 g/day) and the UK (5.71 g/day) were similar. This finding was comparable to those observed by Pellett and Ghosh (Pellett and Ghosh, 2004). Visual examination of these spectra showed that some of the urinary NMR spectra from Japan excreted high urinary levels of lysine and this may be indicative of latent diseases such as cystinuria, or generalized amino aciduria. As we did not include the quantification of metabolites within the COMPASS pipeline, and therefore are unable to provide the concentration of the metabolite. However, future work will include developing a strategy for robust quantification of the metabolites of interest to enable improved differentiation of different disease patterns of interest.

Although we illustrated our approach using NMR data, the method is generic and may be applicable to other large complex dataset such as MS and DNA microarray datasets or multi-modal datasets. Due to the cost effectiveness and high throughput nature of spectroscopic techniques in phenotyping large number of biological samples with high reproducibility, there have been increasing number of studies involving large-scale population cohort studies. A key benefit of the COMPASS approach is thus its capacity for generating rapid semi-automated population statistics, which can be used for multiple purposes. For example: to identify latent diseases, which may show higher prevalence than expected (e.g. cystinuria urinary excretion of lysine in Japan); to confirm disease prevalence as per literature reports (e.g. beta-aminoisobutyric acid in China); and to detect undiagnosed disease patterns (e.g. glycosuria relating to latent type 2 diabetes). In addition, dietary (e.g. proline betaine and alcohol) and drug intake (e.g. ibuprofen and acetaminophen) habits as well as patterns of use of specific foods (e.g. erythritol, which was a commonly used low-calorie sugar substitute in Japan in the 1990s (Regnat *et al.*, 2018) but not available in the UK or US at the time) can also be observed at the population and sub-population level. The ultimate goal is to use this approach to ‘screen’ the population or to define heterogeneity within a population according to disease prevalence or risk based on factors including but not limited to food/drug/supplement intake.

## 5 Conclusion

The COMPASS approach can be used to improve the analysis of large and complex datasets generated within the biomedical sciences. As the current trend in analytical instrumentation is leaning towards collection of more and more variables for more samples, informatics methods need to improve in order not to ‘drown’ in the complexity and size of the data. The concept of division of large datasets into blocks of fewer variables is not novel by any means but the COMPASS approach presented here combines the use of multiple-blocked data with statistical spectroscopy to enhance recovery and quality of metabolic information. However, the main benefit of the application we present here is the capacity for generating rapid semi-automated population statistics.

## Funding

The INTERMAP Study was supported by grants R01-HL50490, R01-HL65461, R01-HL84228, and R01-HL135486 from the National Heart, Lung, and Blood Institute, National Institutes of Health (Bethesda, Maryland, USA) and by the Ministry of Education, Culture, Sports, Science, and Technology of Japan (Grant-in-Aid for Scientific Research [A], No. 090357003 and No. 17H01553), and the UK (a project grant from the West Midlands National Health Service Research and Development, and grant R2019EPH from the Chest, Heart and Stroke Association ). R.L.L. and E.H. are supported by the Department of Jobs, Tourism, Science and Innovation, Government of Western Australian through the Premier’s Science Fellowship Program.

*Conflict of Interest*: none declared.

## Supplementary Material

btaa649_Supplementary_Data

## References

[btaa649-B1] Cloarec O. et al (2005) Statistical total correlation spectroscopy: an exploratory approach for latent biomarker identification from metabolic 1H NMR data sets. Anal. Chem., 77, 1282–1289. 15732908 10.1021/ac048630x

[btaa649-B2] Dieterle F. et al (2006) Probabilistic quotient normalization as robust method to account for dilution of complex biological mixtures. Application in 1H NMR metabonomics. Anal. Chem., 78, 4281–4290. 16808434 10.1021/ac051632c

[btaa649-B3] Elliott P. et al (2015) Urinary metabolic signatures of human adiposity. Sci. Transl. Med., 7, 285–262. 10.1126/scitranslmed.aaa5680 PMC6598200 25925681

[btaa649-B4] Heinzmann S.S. et al (2010) Metabolic profiling strategy for discovery of nutritional biomarkers: proline betaine as a marker of citrus consumption. Am. J. Clin. Nutr., 92, 436–443. 20573794 10.3945/ajcn.2010.29672 PMC2904656

[btaa649-B5] Holmes E. et al (2008) Human metabolic phenotype diversity and its association with diet and blood pressure. Nature, 453, 396–400. 18425110 10.1038/nature06882 PMC6556779

[btaa649-B6] Jackson J.E. (1991) A User's Guide to Principal Components. John Wiley & Sons, Danvers, MA.

[btaa649-B26] Kimhofer T. (2020) MetaboMate: All you need for NMR-based metabolic profiling with R!, https://github.com/tkimhofer/MetaboMate.

[btaa649-B7] Li J.V. et al (2011a) Metabolic surgery profoundly influences gut microbial-host metabolic cross-talk. Gut, 60, 1214–1223. 21572120 10.1136/gut.2010.234708 PMC3677150

[btaa649-B8] Li J.V. et al (2011b) Experimental bariatric surgery in rats generates a cytotoxic chemical environment in the gut contents. Front. Microbiol., 2, 183. 21949514 10.3389/fmicb.2011.00183 PMC3171674

[btaa649-B9] Loo R.L. et al (2012) A comparison of self-reported analgesic use and detection of urinary ibuprofen and acetaminophen metabolites by means of metabonomics: the INTERMAP Study. Am. J. Epidemiol., 175, 348–358. 22223708 10.1093/aje/kwr292 PMC3271812

[btaa649-B10] Loo R.L. et al (2009) Metabolic profiling and population screening of analgesic usage in nuclear magnetic resonance spectroscopy-based large-scale epidemiologic studies. Anal. Chem., 81, 5119–5129. 19489597 10.1021/ac900567e PMC2726443

[btaa649-B11] McKay R.T. (2011) How the 1D-NOESY suppresses solvent signal in metabonomics NMR spectroscopy: an examination of the pulse sequence components and evolution. Concepts Magn. Reson. A, 38A, 197–220.

[btaa649-B12] Nicholson J.K. et al (1995) 750 MHz 1H and 1H-13C NMR spectroscopy of human blood plasma. Anal. Chem., 67, 793–811. 7762816 10.1021/ac00101a004

[btaa649-B13] Pellett P.L., Ghosh S. (2004) Lysine fortification: past, present, and future. Food Nutr. Bull., 25, 107–113. 15214255 10.1177/156482650402500201

[btaa649-B14] Regnat K. et al (2018) Erythritol as sweetener-wherefrom and whereto? Appl. Microbiol. Biotechnol., 102, 587–595. 29196787 10.1007/s00253-017-8654-1 PMC5756564

[btaa649-B15] Smith L.M. et al (2007) Statistical correlation and projection methods for improved information recovery from diffusion-edited NMR spectra of biological samples. Anal Chem., 79, 5682-5689. 10.1021/ac0703754 17585837

[btaa649-B16] Stamler J. et al (2003) INTERMAP: background, aims, design, methods, and descriptive statistics (nondietary). J. Hum. Hypertens., 17, 591–608. 13679950 10.1038/sj.jhh.1001603 PMC6660162

[btaa649-B17] Teague C. et al (2004) Ethyl glucoside in human urine following dietary exposure: detection by 1H NMR spectroscopy as a result of metabonomic screening of humans. Analyst, 129, 259–264. 14978530 10.1039/b314316n PMC6556765

[btaa649-B18] Tennenhaus M. (1998) PLS regression methods. J. Chemo Metrics, 211–228.

[btaa649-B19] Westerhuis J. et al (1998) Analysis of multiblock and hierarchical PCA and PLS models. J. Chemom., 12, 301–321.

[btaa649-B20] Wist J. (2019) HastaLaVista, a web-based user interface for NMR-based untargeted metabolic profiling analysis in biomedical sciences: towards a new publication standard. J. Cheminform., 11, 10.1186/s13321-019-0399-7 PMC6896291 33430999

[btaa649-B21] Wold S. et al (2002) New and old trends in chemometrics. How to deal with the increasing data volumes in R&D&P (research, development and production)—with examples from pharmaceutical research and process modeling. J. Chemom., 16, 377–386.

[btaa649-B22] Wold S. et al (1987) Principal component analysis. Chemom. Intell. Lab. Syst., 2, 37–52.

[btaa649-B23] Wold S. et al (2001a) PLS-regression: a basic tool of chemometrics. Chemom. Intell. Lab. Syst., 58, 109–130.

[btaa649-B24] Wold S. et al (2001b) Some recent developments in PLS modeling. Chemom. Intell. Lab. Syst., 58, 131–150.

[btaa649-B25] Yanai J. et al (1969) Genetic study of beta-aminoisobutyric acid excretion by Japanese. Am. J. Hum. Genet., 21, 115–132. 5814159 PMC1706433

