# Supplementary material for: Strategy for improved characterization of human metabolic phenotypes using a COmbined Multi-block Principal components Analysis with Statistical Spectroscopy (COMPASS)
Source: Bioinformatics. 2020 Jul 21;36(21):5229–36. doi: 10.1093/bioinformatics/btaa649 (PMC7850059; doi:10.1093/bioinformatics/btaa649)
Supplement: btaa649_Supplementary_Data [file btaa649_supplementary_data.zip › Supp 1_COMPASSdescription.docx]

**Supplementary Material 1: Description of the data processing pipeline**

Two R scripts and four JSON files are required for the COMPASS and provided as supplementary documents within this manuscript: multiblocking.R (Supplementary Material 2), MBCC-metaboliteX.Rmd (Supplementary Material 3), modelExplorer.view.json (version 1.0 and 1.1, Supplementary Material 4) and crossCorrelationExplorer.view.json (version 0.1 and 0.1, Supplementary Material 5). The modelExplorer.view.json 1.1 and crossCorrelationExplorer.view.json 0.1 are more suited for file sizes >200 Mb (see Supplementary Table 1. Current and updated versions of these files are available on the repository <https://github.com/cheminfo/COMPASS>. The multiblocking.R file enables a user to define a set of spectral regions, computes a PCA (using github.com/kimsche/MetaboMate) for each corresponding spectral region and prepares the results for visualization into a web-browser (tested only on Chrome). The interactive visualization in the browser is enabled by the *hastaLaVista* package^1^ available from <https://github.com/jwist/hastaLaVista> and both the JSON files provided herein defines the visualization layout.

The MBCC-metaboliteX.Rmd R script enables identification of reference patterns corresponding to molecules of interest and produce a report in pdf or docx format. In addition it offers the possibility to visualize the results in the browser with the use of hastaLaVista R-package and the dedicated crossCorrelationExplorer.view.json file.

**Supplementary Table 1:** A description of the input and output data for each of the R script and JSON files are shown below:

| **Filename** | **Input** | **Output** |
| --- | --- | --- |
| multiblocking.R | A matrix with data and its associated metadata | A JSON file with corresponding multiblock of PCA models for each selected portion of spectrum and a link to the webpage for interactive visualization of the results |
| MBCC-metaboliteX.Rmd | A matrix with data, a reference pattern (defined using STOCSY) and information about categories or group (obtained from metadata matrix for presenting the results) | Display number of features founds for each category or group |
| modelExplorer_1_0.view.json | This file is not intended to be edited directly by the user for input of data but it is necessary for hastaLaVista package to enable interactive data visualization | Display the results of the multiblocking.R script. Users can use this json file to output the data when the produced data.json is smaller than 200Mb |
| modelExplorer_1_1.view.json | This file is not intended to be edited directly by the user for input of data but it is necessary for hastaLaVista package to enable interactive data visualization | Display the results of the multiblocking.R script. Users can use this json file to output the data when the produced data.json is larger than 200Mb |
| crossCorrelationExplorer_0_0.view.json | This file is not intended to be edited directly by the user for input of data but it is necessary for hastaLaVista package to enable interactive data visualization | It provides the layout to explore the results of the cross-correlation analysis. This version works for large datasets that are too large to be directly loaded into the browser. URLs of the spectra data must provided in the R labbook script. |
| crossCorrelationExplorer_0_1.view.json | This file is not intended to be edited directly by the user for input of data but it is necessary for hastaLaVista package to enable interactive data visualization | It provides the layout to explore the results of the cross-correlation analysis. This version works small dataset that can be loaded directly in the browser. |

**Installation of R-packages**

For installation MetaboMate and hastaLaVista R-packages, see <https://github.com/kimsche/MetaboMate> and <https://github.com/jwist/hastaLaVista>, respectively.

We have embedded JavaScript library within hastaLaVista R packages. R compiler flags this as a warning during installation. However, this should not cause any problem in using the hastaLaVista. We have included an animal dataset (<https://github.com/cheminfo/COMPASS>) from a surgically-induced weight loss (Supplementary material 6 for study descriptions) in order to enable the users to explore the capability of the software. We have used this dataset here for demonstration purposes only.

**Using COMPASS data analysis interface**

After installation of these packages, place all the supplementary materials 2 -5 into the R environment (or download the latest version directly from COMPASS repository <https://github.com/cheminfo/COMPASS>).

The *multiblocking.R* script will output a webpage link which can be viewed in the default browser (only tested for Chrome) as shown in Supplementary Figure 1A. Within the multiblocking.R script, users can input the data region for each PCA block (in lines 25 – 37, and it is currently set as 0.5ppm for each PCA block).


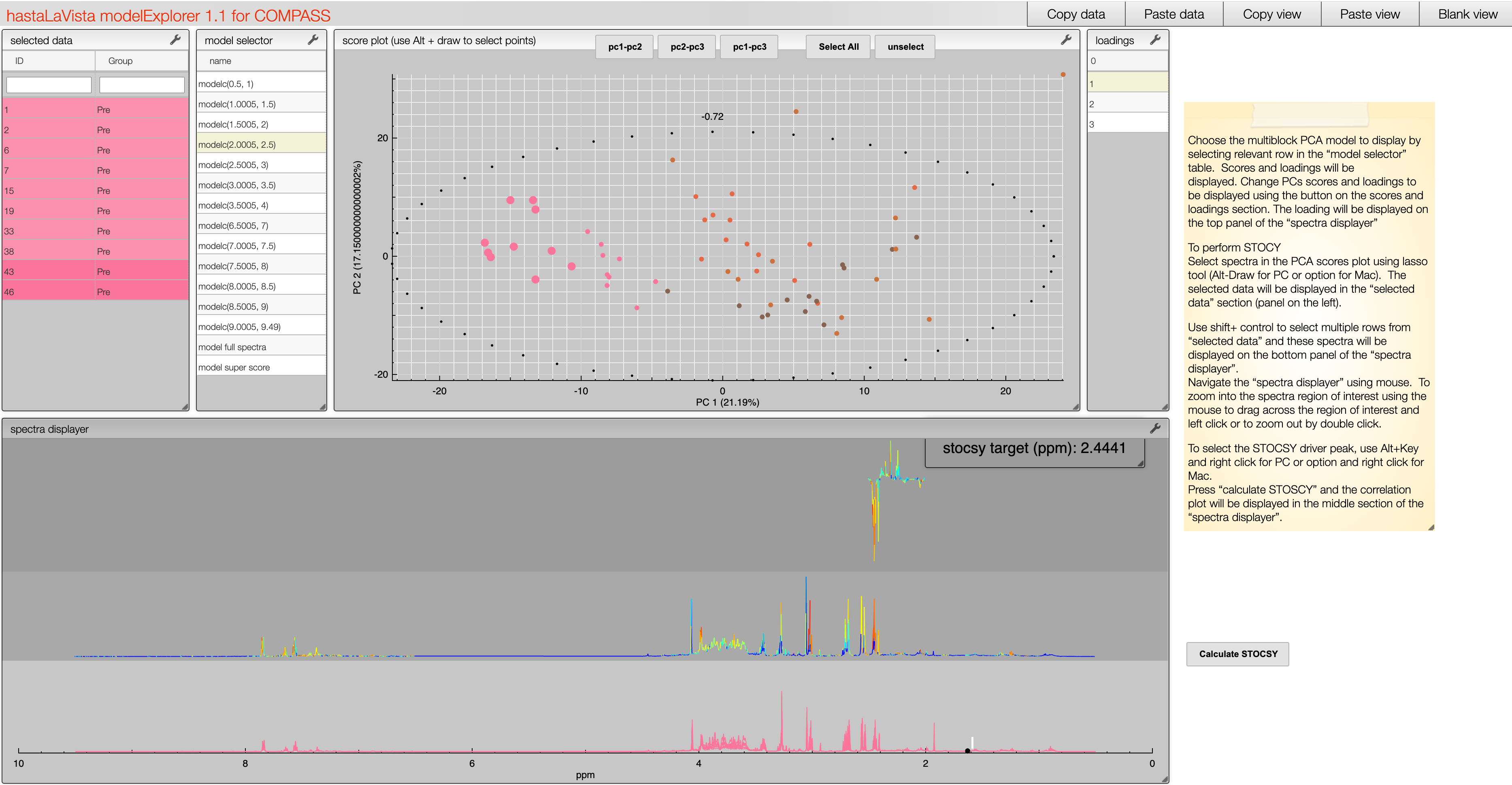


**Supplementary Figure 1A.** Screenshot of the modelExplorer view launched by multiblocking.R. Such a webpage visualization is known as vista in Spanish (view in English). The text box in yellow on the right describes the functionality within the modelExplorer view. Within the modelExplorer environment, users can select relevant samples to calculate STOCSY. The STOCSY correlation plot shows resonances related to the STOCSY “driver” peak within the whole spectra region rather than on the selected ranges of the multiblock-PCA. This enables visualization of the correlations and therefore aid the identification of the molecules of interest.

The metaboliteX.Rmd. function (Supplementary material 3) will generate the distribution of the cross-correlation (Supplementary Figure 1B), output the estimated statistics of the dataset in docx format (using “knit” function on the menu), and output a webpage link which can be viewed interactively in the default browser (Supplementary Figure 1C).

**
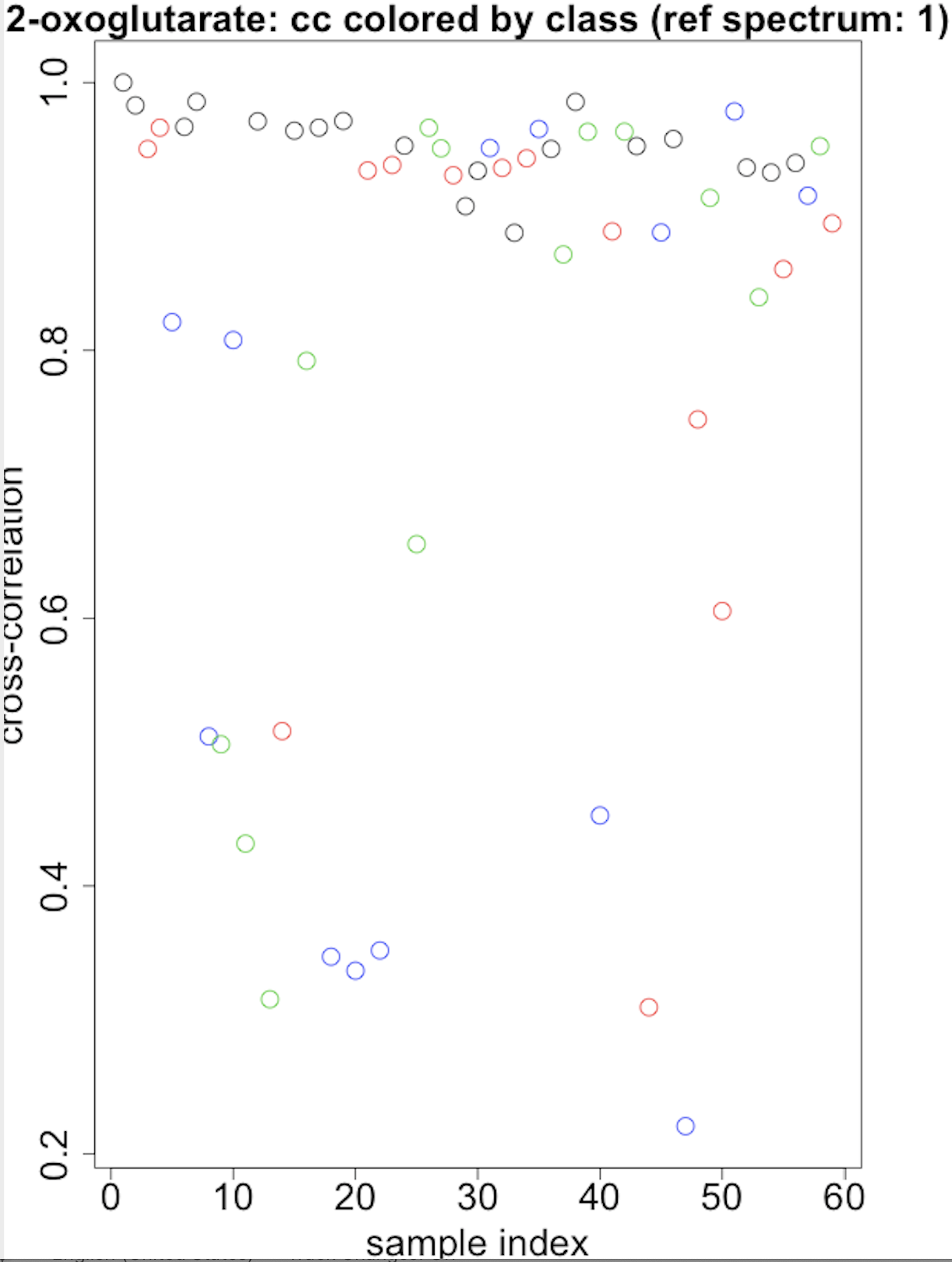
**

**Supplementary Figure 1B:** The distribution of the cross-correlation for 2-oxoglutarate at 2.44ppm and color coded to class (here refers to different time points for the bariatric dataset). The percentage and number of spectra showing 2-oxoglutarate metabolite based on the pattern is shown in the statistics table (as shown below).

## 2-oxoglutarate statistics

### Percentage of samples with 2-oxoglutarate by Class

##

## Pre W2 W6 W8

## 100.0 71.4 53.8 38.5

### Total number of samples with 2-oxoglutarate by Class

##

## Pre W2 W6 W8

## 19 10 7 5

### Total number of samples in each Class

##

## Pre W2 W6 W8

## 19 14 13 13

### Total number of samples with 2-oxoglutarate

## [1] 41

The *crossCorrExplorer_0_1.view.json* file from the hastaLaVista package enables an interactive exploration and visual validation of the results from metaboliteX.Rmd (Supplementary Figure 1C).


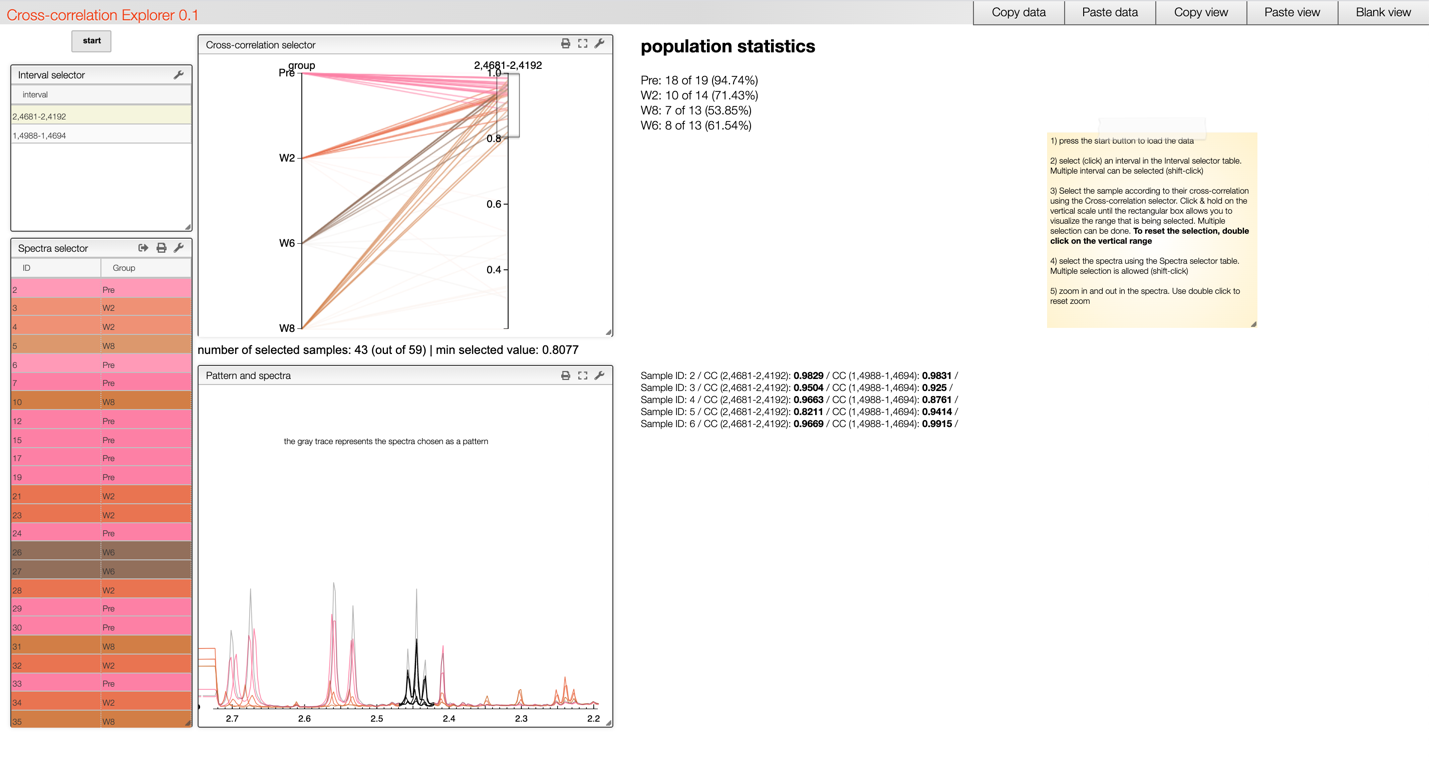


**Supplementary Figure 1C.** The cross-correlation explorer tool was incorporated as part of the *hastaLaVista* R package. The yellow text box provides a description on how to navigate this tool.

**References:**

1. Wist J HastaLaVista, a web-based user interface for NMR-based untargeted metabolic profiling analysis in biomedical sciences: towards a new publication standard. J Cheminform 2019; 11 (75).

2. Cloarec O, Dumas ME, Trygg J, Craig A, Barton RH, Lindon JC *et al* Evaluation of the orthogonal projection on latent structure model limitations caused by chemical shift variability and improved visualization of biomarker changes in 1H NMR spectroscopic metabonomic studies. Anal Chem 2005; 77: 517-526.

3. Cloarec O, Dumas ME, Craig A, Barton RH, Trygg J, Hudson J *et al* Statistical total correlation spectroscopy: an exploratory approach for latent biomarker identification from metabolic 1H NMR data sets. Anal Chem 2005; 77: 1282-1289.
