# Supplementary material for: Strategy for improved characterization of human metabolic phenotypes using a COmbined Multi-block Principal components Analysis with Statistical Spectroscopy (COMPASS)
Source: Bioinformatics. 2020 Jul 21;36(21):5229–36. doi: 10.1093/bioinformatics/btaa649 (PMC7850059; doi:10.1093/bioinformatics/btaa649)
Supplement: btaa649_Supplementary_Data [file btaa649_supplementary_data.zip › Supp 10_Lysine.pdf]

Supplementary Material 10: Typical output for lysine using COMPASS approach

Supplementary Figure 10A: Robust reference patterns of lysine at 1.4 – 1.55ppm, 1.67 - 1.80 ppm and 1.85-1.97ppm as identified using STOCSY

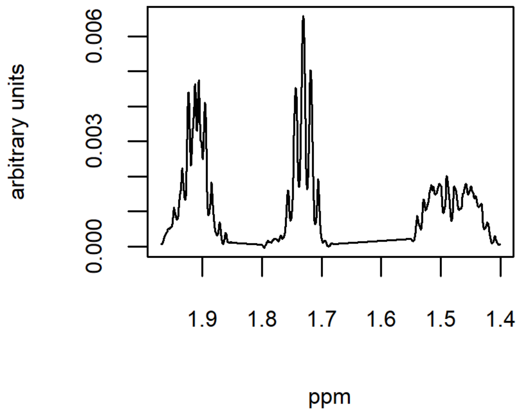

Supplementary Figure 10B: Distribution of cross-correlation using robust reference patterns of lysine as shown in Supplementary Figure 10A color coded to countries: China (red), Japan (turquoise), UK (blue), and USA (grey).

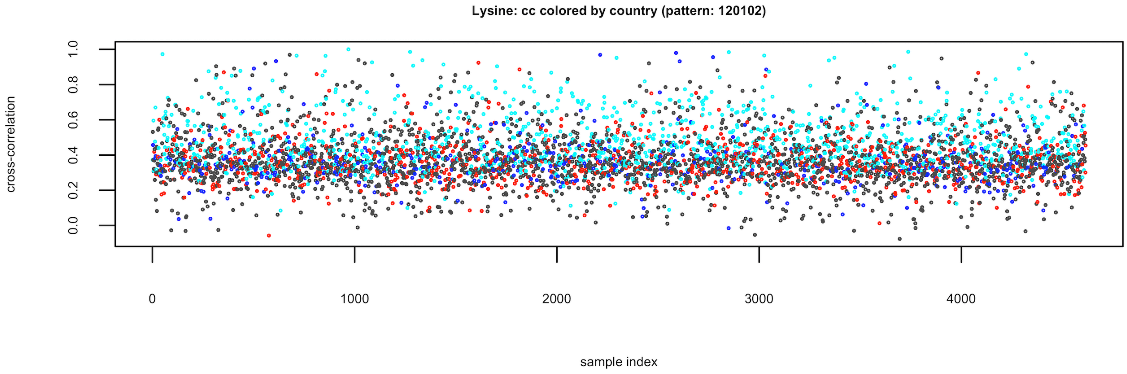

Supplementary Figure 10C: NMR spectra in the dataset showing lysine pattern with high cross correlation threshold (CC) value > 0.8(in green), intermediate CC between 0.6 to 0.8 (in amber) and low threshold showing no feature at CC < 0.6 (in red). We have presented 6 randomly selected spectra in each category. Note, users may choose to output more spectra within the COMPASS framework.

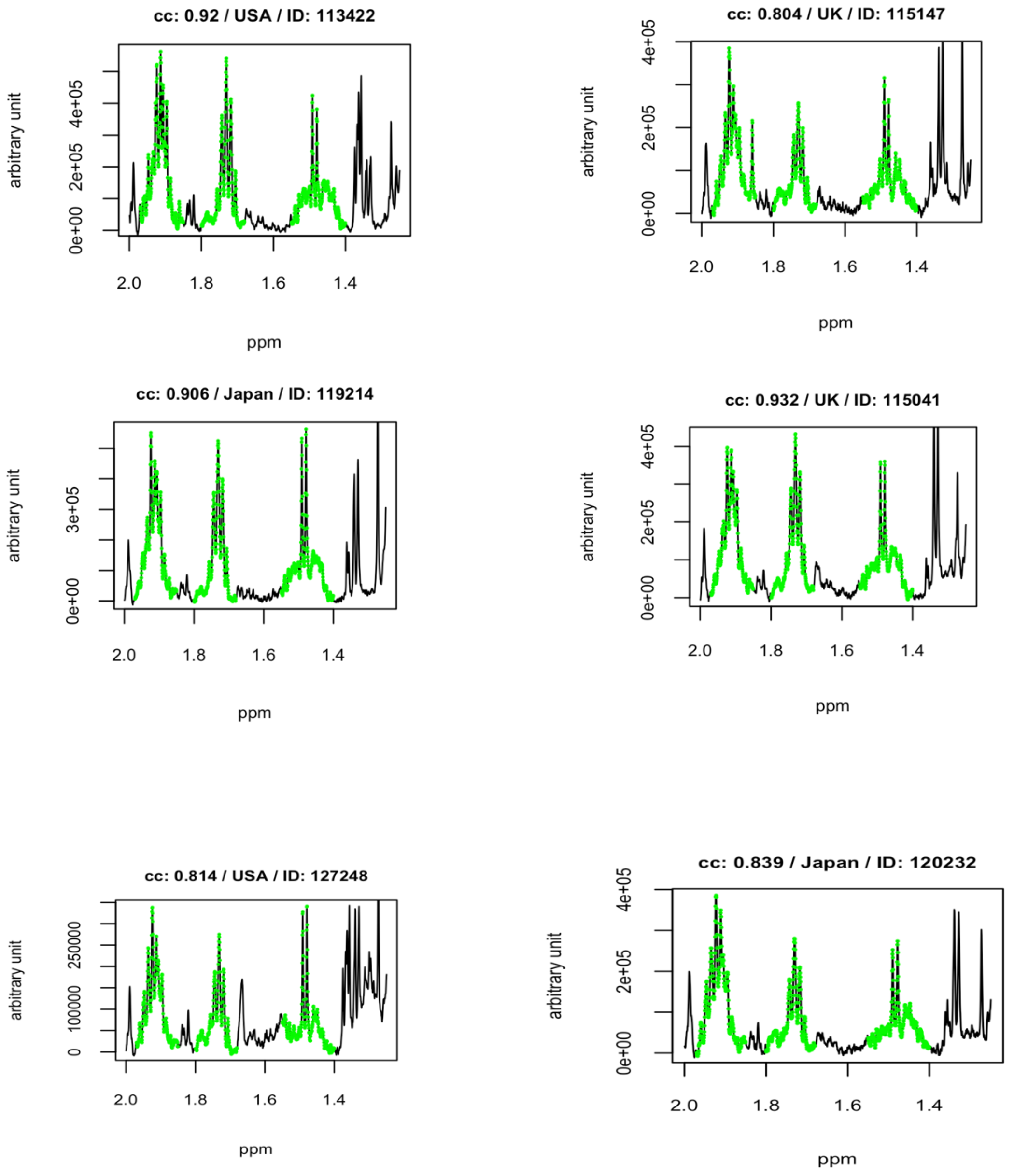

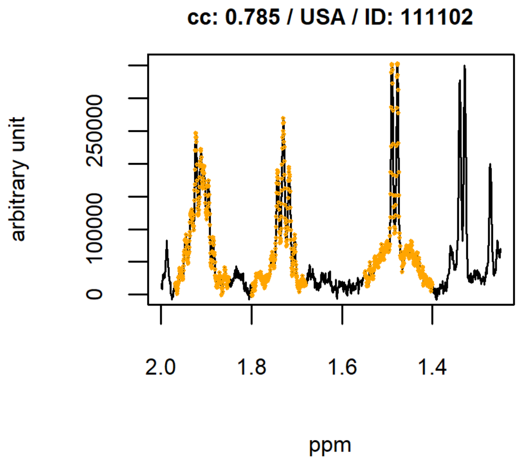
cc: 0.785 / USA / ID: 111102
arbitrary unit
0
100000
250000
2.0
1.8
1.6
1.4
ppm

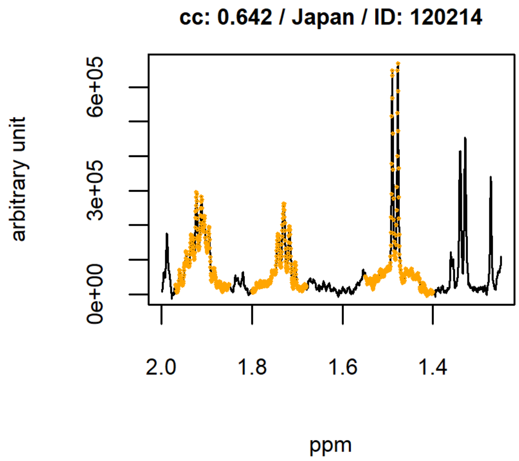
cc: 0.642 / Japan / ID: 120214
arbitrary unit
0e+00
3e+05
6e+05
2.0
1.8
1.6
1.4
ppm

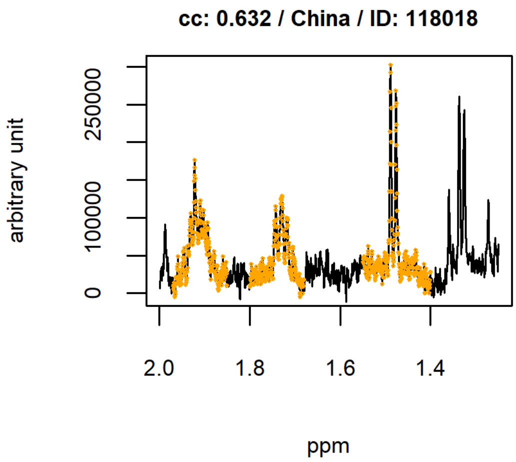
cc: 0.632 / China / ID: 118018
arbitrary unit
0
100000
250000
2.0
1.8
1.6
1.4
ppm

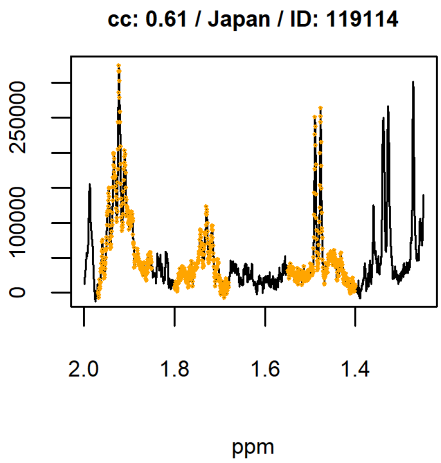
cc: 0.61 / Japan / ID: 119114
0
100000
250000
2.0
1.8
1.6
1.4
ppm

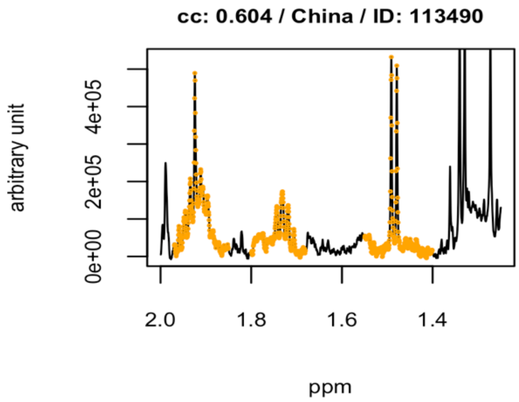
cc: 0.604 / China / ID: 113490
arbitrary unit
0e+00
2e+05
4e+05
2.0
1.8
1.6
1.4
ppm

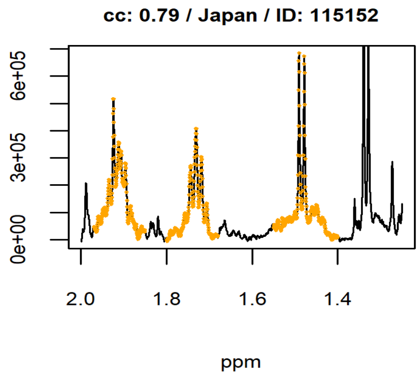
cc: 0.79 / Japan / ID: 115152
0e+00
3e+05
6e+05
2.0
1.8
1.6
1.4
ppm

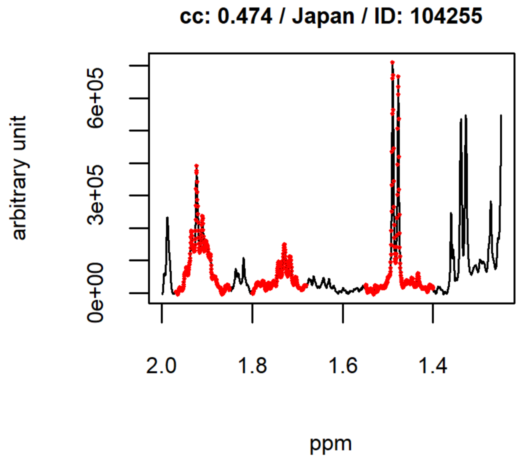
cc: 0.474 / Japan / ID: 104255
arbitrary unit
0e+00
3e+05
6e+05
2.0
1.8
1.6
1.4
ppm

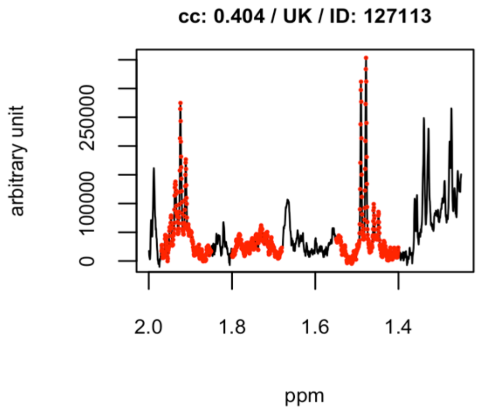
cc: 0.404 / UK / ID: 127113
arbitrary unit
0
100000
250000
2.0
1.8
1.6
1.4
ppm

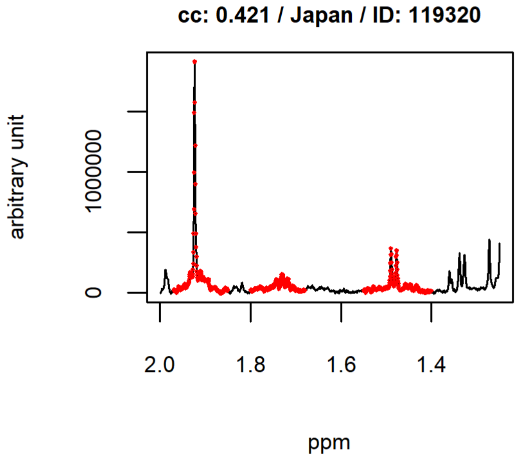
cc: 0.421 / Japan / ID: 119320
arbitrary unit
0
1000000
2.0
1.8
1.6
1.4
ppm

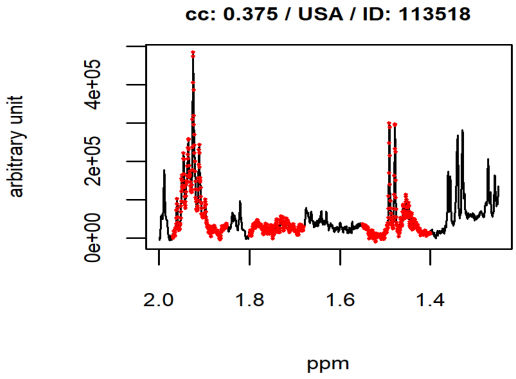
cc: 0.375 / USA / ID: 113518
arbitrary unit
0e+00
2e+05
4e+05
2.0
1.8
1.6
1.4
ppm

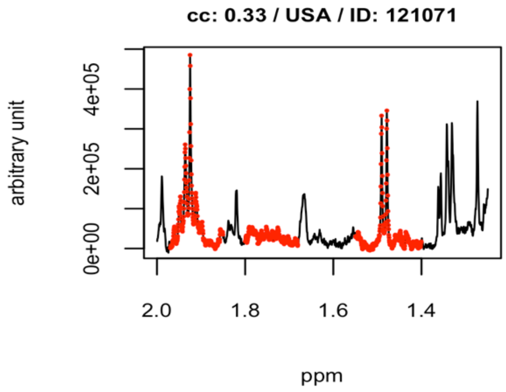
cc: 0.33 / USA / ID: 121071
arbitrary unit
0e+00
2e+05
4e+05
2.0
1.8
1.6
1.4
ppm

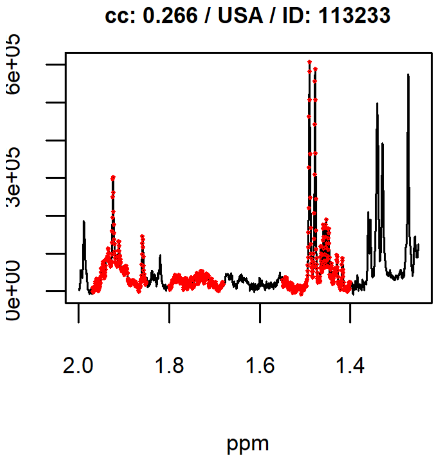
cc: 0.266 / USA / ID: 113233
0e+00
3e+05
6e+05
2.0
1.8
1.6
1.4
ppm

Supplementary Table 10: Population statistics for lysine using COMPASS approach

Percentage of samples with lysine in the urine and by country

```
##
## China Japan    UK   USA
##  3.9     19.5     5.2    7.8
```

Total number of samples with proline betaine in the urine and by country

```
##
## China Japan    UK   USA
##  32      222      26     167
```

Total number of samples with proline betaine in the urine

```
## [1] 447
```
