# Supplementary material for: Strategy for improved characterization of human metabolic phenotypes using a COmbined Multi-block Principal components Analysis with Statistical Spectroscopy (COMPASS)
Source: Bioinformatics. 2020 Jul 21;36(21):5229–36. doi: 10.1093/bioinformatics/btaa649 (PMC7850059; doi:10.1093/bioinformatics/btaa649)
Supplement: btaa649_Supplementary_Data [file btaa649_supplementary_data.zip › Supp 11_ProlineBetaine.pdf]

Supplementary Material 11: Typical output for Proline Betaine using COMPASS approach

Supplementary Figure 11A: Robust reference patterns of proline betaine at 3.1-3.12ppm and 3.29 – 3.31ppm as identified using STOCSY

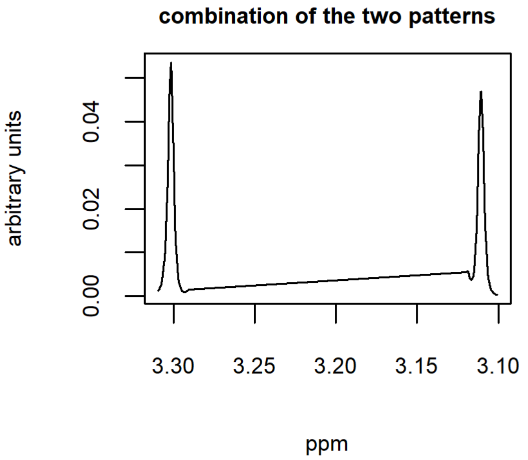

Supplementary Figure 11B: Distribution of cross-correlation using robust reference pattern of proline betaine as shown in Supplementary Figure 11A color coded to countries: China (red), Japan (turquoise), UK (blue), and USA (grey).

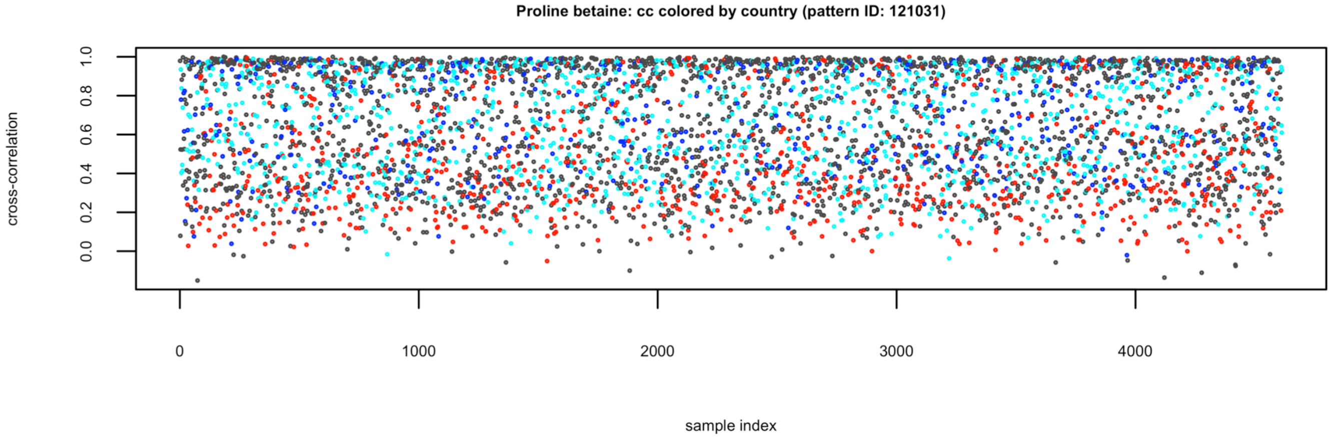

Supplementary Figure 11C: NMR spectra in the dataset showing proline betaine pattern with high cross correlation threshold (CC) value > 0.90 (in green), intermediate CC between 0.85 to 0.90 (in amber) and low threshold showing no feature at CC < 0.85 (in red). We have presented 6 randomly selected spectra in each category. Note, users may choose to output more spectra within the COMPASS framework.

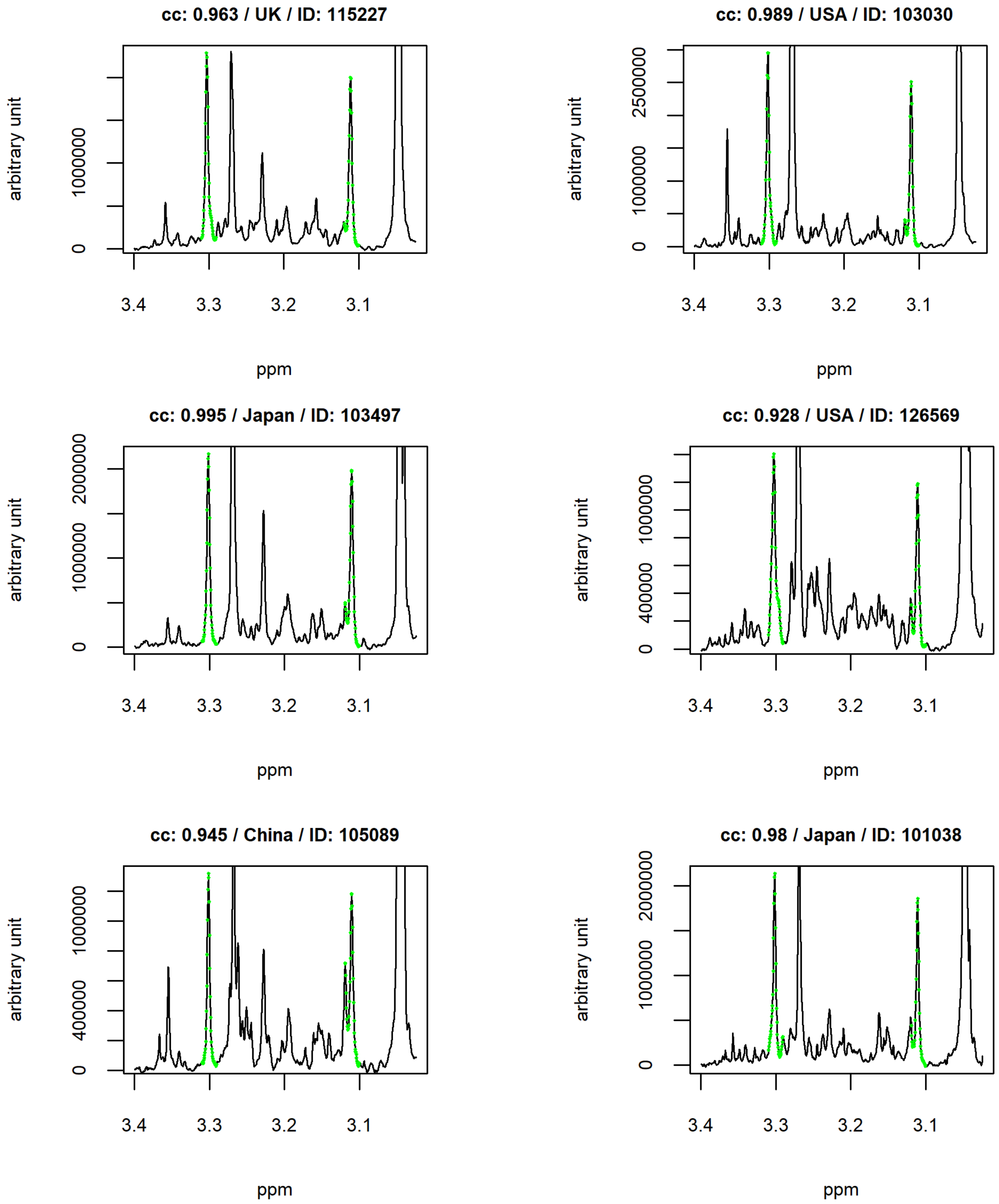

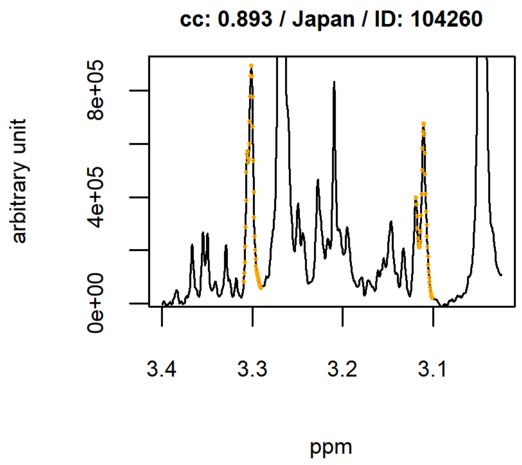
cc: 0.893 / Japan / ID: 104260
arbitrary unit
0e+00
4e+05
8e+05
3.4
3.3
3.2
3.1
ppm

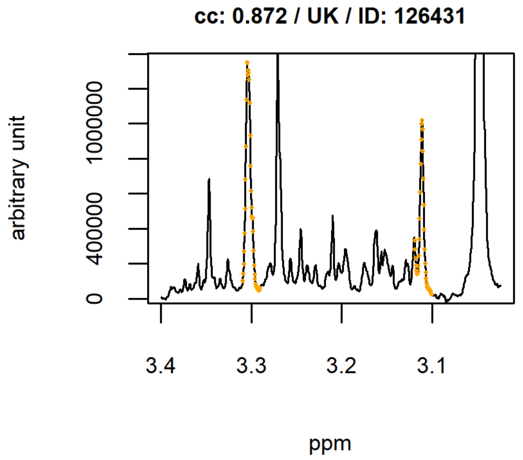
cc: 0.872 / UK / ID: 126431
arbitrary unit
0
400000
1000000
3.4
3.3
3.2
3.1
ppm

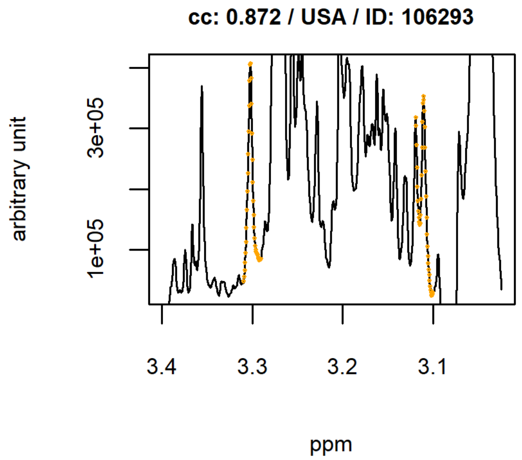
cc: 0.872 / USA / ID: 106293
arbitrary unit
1e+05
3e+05
3.4
3.3
3.2
3.1
ppm

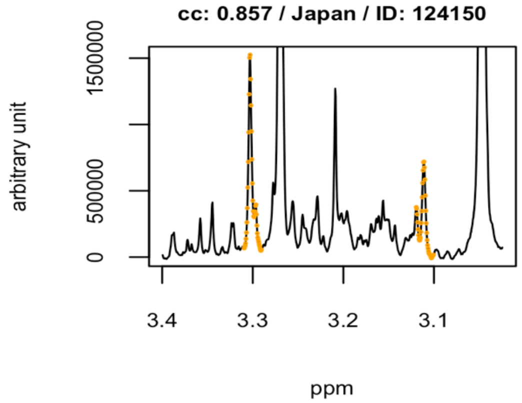
cc: 0.857 / Japan / ID: 124150
arbitrary unit
0
500000
1500000
3.4
3.3
3.2
3.1
ppm

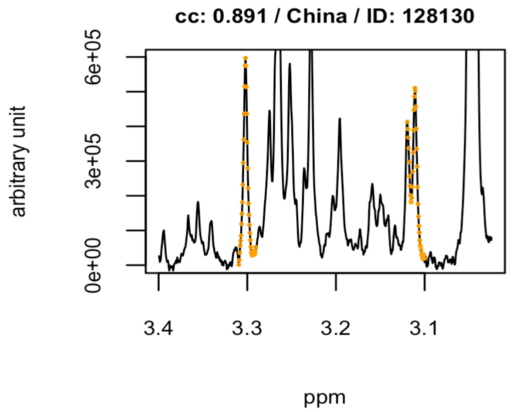
cc: 0.891 / China / ID: 128130
arbitrary unit
0e+00
3e+05
6e+05
3.4
3.3
3.2
3.1
ppm

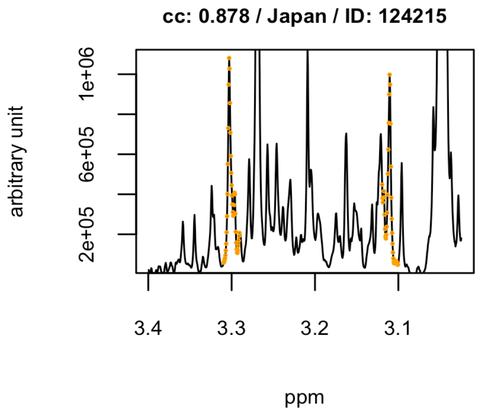
cc: 0.878 / Japan / ID: 124215
arbitrary unit
2e+05
6e+05
1e+06
3.4
3.3
3.2
3.1
ppm

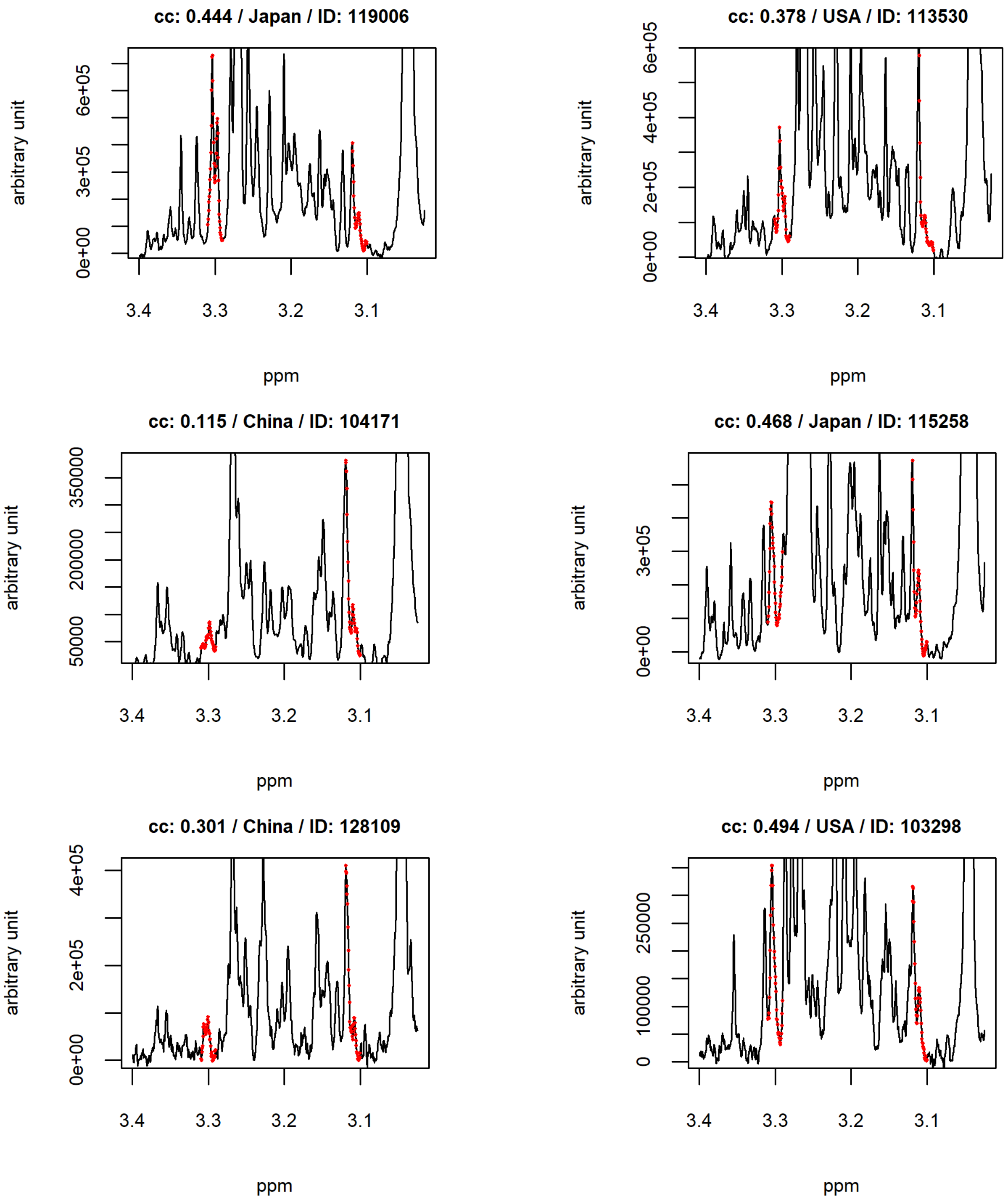
cc: 0.444 / Japan / ID: 119006
arbitrary unit
0e+00
3e+05
6e+05
3.4
3.3
3.2
3.1
ppm
cc: 0.378 / USA / ID: 113530
arbitrary unit
0e+00
2e+05
4e+05
6e+05
3.4
3.3
3.2
3.1
ppm
cc: 0.115 / China / ID: 104171
arbitrary unit
50000
200000
350000
3.4
3.3
3.2
3.1
ppm
cc: 0.468 / Japan / ID: 115258
arbitrary unit
0e+00
3e+05
3.4
3.3
3.2
3.1
ppm
cc: 0.301 / China / ID: 128109
arbitrary unit
0e+00
2e+05
4e+05
3.4
3.3
3.2
3.1
ppm
cc: 0.494 / USA / ID: 103298
arbitrary unit
0
100000
250000
3.4
3.3
3.2
3.1
ppm

Supplementary Table 11: Population statistics for proline betaine using COMPASS approach

Percentage of samples with proline betaine in the urine and by country

```
##
## China Japan    UK   USA
##  16.7  28.8  35.5  36.8
```

Total number of samples with proline betaine in the urine and by country

```
##
## China Japan    UK   USA
##   138   328   176   793
```

Total number of samples with proline betaine in the urine

```
## [1] 1435
```
