# Supplementary material for: Strategy for improved characterization of human metabolic phenotypes using a COmbined Multi-block Principal components Analysis with Statistical Spectroscopy (COMPASS)
Source: Bioinformatics. 2020 Jul 21;36(21):5229–36. doi: 10.1093/bioinformatics/btaa649 (PMC7850059; doi:10.1093/bioinformatics/btaa649)
Supplement: btaa649_Supplementary_Data [file btaa649_supplementary_data.zip › Supp 12_Ethanol.pdf]

Supplementary Material 12: Typical output for COMPASS approach as illustrated using ethanol

Supplementary Figure 12A: Robust reference pattern of ethanol as identified using STOCSY.

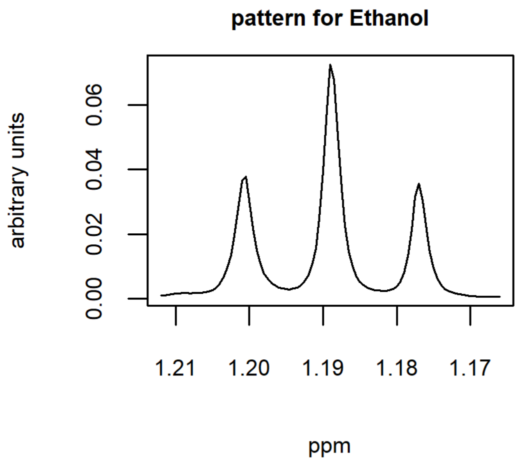

Supplementary Figure 12B: Distribution of cross-correlation using robust reference pattern of ethanol as shown in Supplementary Figure 12A and color coded to countries: China (red), Japan (turquoise), UK (blue), and USA (grey).

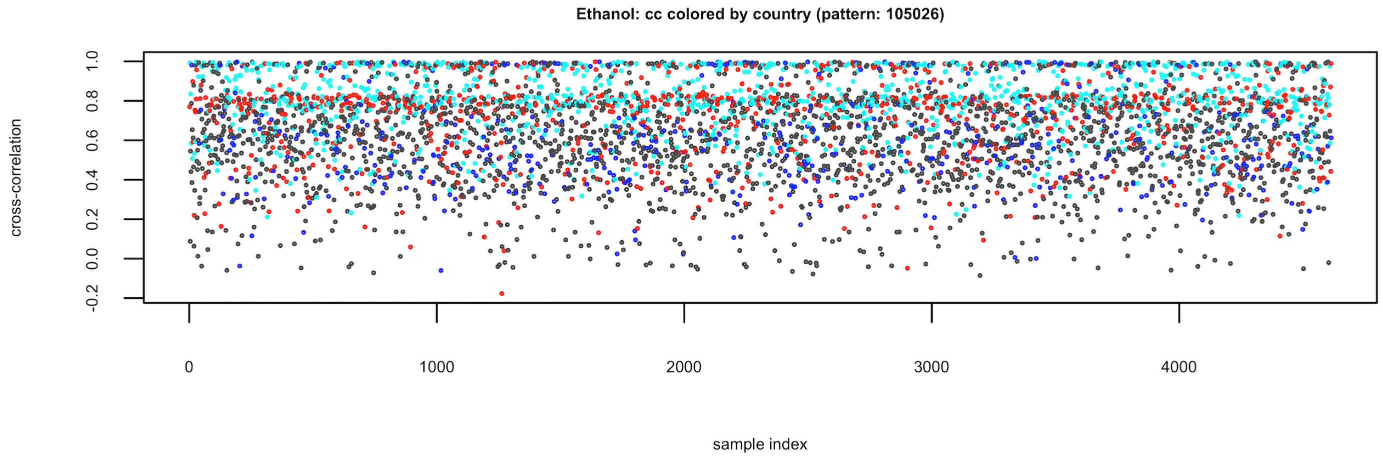

Supplementary Figure 12C: NMR spectra in the dataset showing ethanol pattern with high cross correlation threshold (CC) value > 0.9 (in green), intermediate CC between 0.85 to 0.9 (in amber) and low threshold showing no feature at CC < 0.85 (in red). We have presented 6 randomly selected spectra in each category. Note, users may choose to output more spectra within the COMPASS framework.

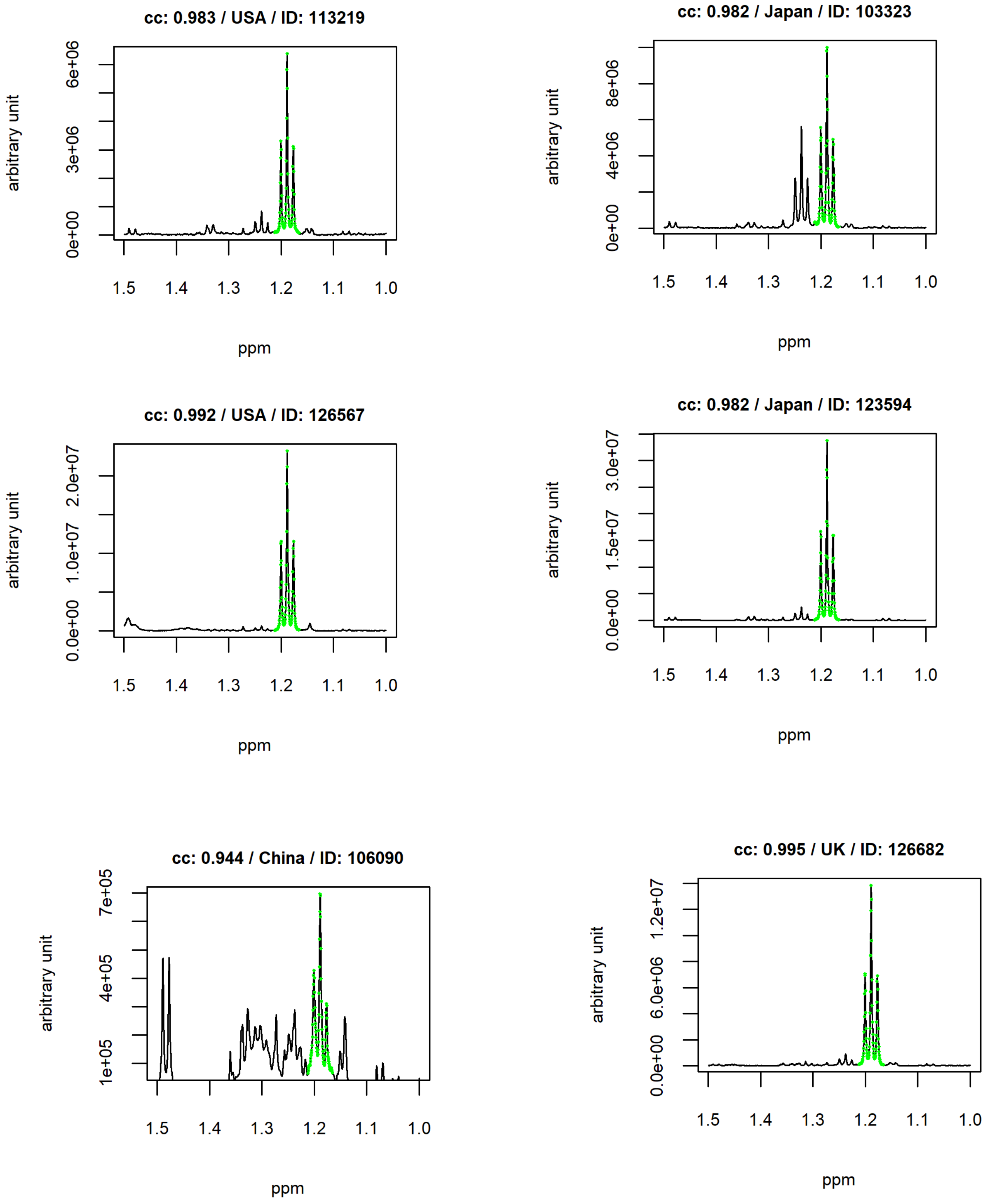

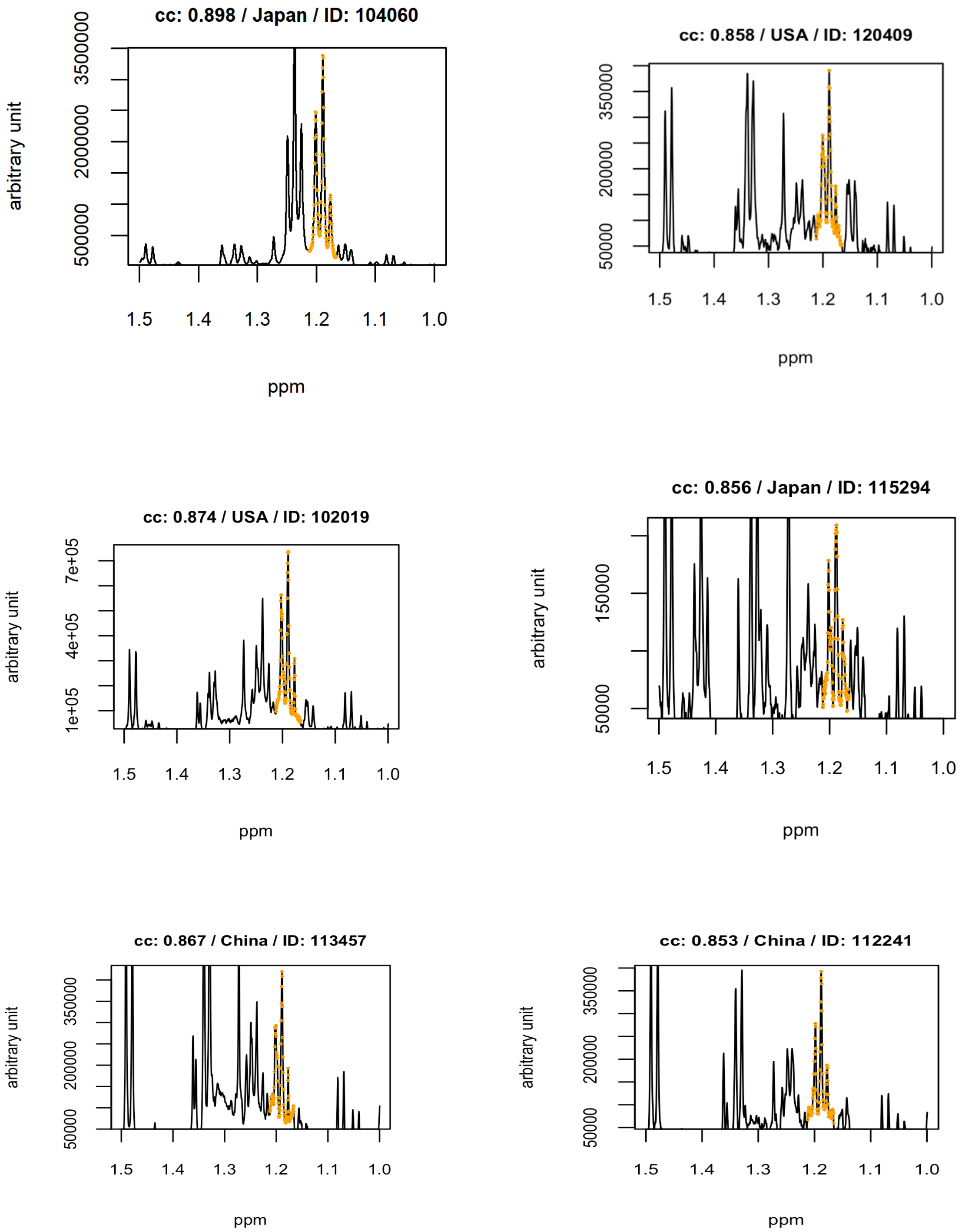
cc: 0.898 / Japan / ID: 104060
arbitrary unit
500000
2000000
3500000
1.5
1.4
1.3
1.2
1.1
1.0
ppm
cc: 0.858 / USA / ID: 120409
50000
200000
350000
1.5
1.4
1.3
1.2
1.1
1.0
ppm
cc: 0.874 / USA / ID: 102019
arbitrary unit
1e+05
4e+05
7e+05
1.5
1.4
1.3
1.2
1.1
1.0
ppm
cc: 0.856 / Japan / ID: 115294
arbitrary unit
50000
150000
1.5
1.4
1.3
1.2
1.1
1.0
ppm
cc: 0.867 / China / ID: 113457
arbitrary unit
50000
200000
350000
1.5
1.4
1.3
1.2
1.1
1.0
ppm
cc: 0.853 / China / ID: 112241
arbitrary unit
50000
200000
350000
1.5
1.4
1.3
1.2
1.1
1.0
ppm

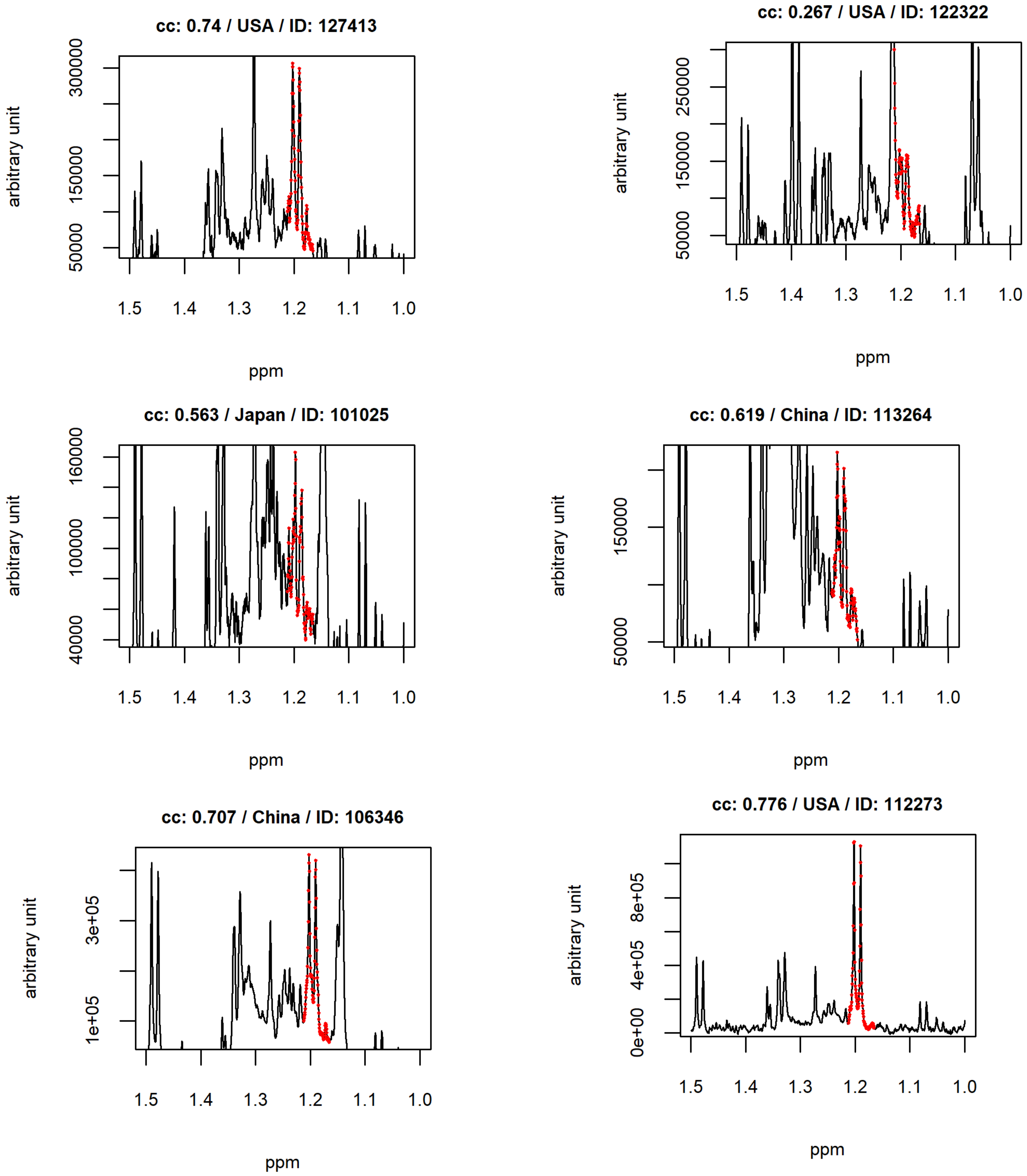
cc: 0.74 / USA / ID: 127413
cc: 0.267 / USA / ID: 122322
cc: 0.563 / Japan / ID: 101025
cc: 0.619 / China / ID: 113264
cc: 0.707 / China / ID: 106346
cc: 0.776 / USA / ID: 112273
arbitrary unit
ppm
1.5
1.4
1.3
1.2
1.1
1.0
50000
150000
300000
250000
40000
100000
160000
1e+05
3e+05
0e+00
4e+05
8e+05

Supplementary Table 12: Population statistics for ethanol using COMPASS approach

Percentage of samples with ethanol metabolite in the urine and by country

```
##
## China Japan    UK   USA
##  17.4    35.6      17.1  12.4
```

Total number of samples with ethanol metabolite in the urine and by country

```
##
## China Japan    UK   USA
##   144   405        85   268
```

Total number of samples with ethanol metabolite in the urine

```
## [1] 902
```
