# Supplementary material for: Strategy for improved characterization of human metabolic phenotypes using a COmbined Multi-block Principal components Analysis with Statistical Spectroscopy (COMPASS)
Source: Bioinformatics. 2020 Jul 21;36(21):5229–36. doi: 10.1093/bioinformatics/btaa649 (PMC7850059; doi:10.1093/bioinformatics/btaa649)
Supplement: btaa649_Supplementary_Data [file btaa649_supplementary_data.zip › Supp 13_Erythritol.pdf]

Supplementary Material 13: Typical output for COMPASS approach as illustrated using erythritol

Supplementary Figure 13A: Robust reference pattern of erythritol as identified using STOCSY

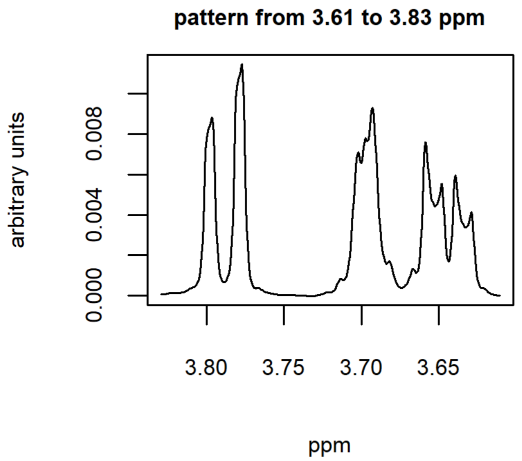

Supplementary Figure 13B: Distribution of cross-correlation using robust reference pattern of erythritol as shown in Supplementary Figure 13A and color coded to countries: China (red), Japan (turquoise), UK (blue), and USA (grey).

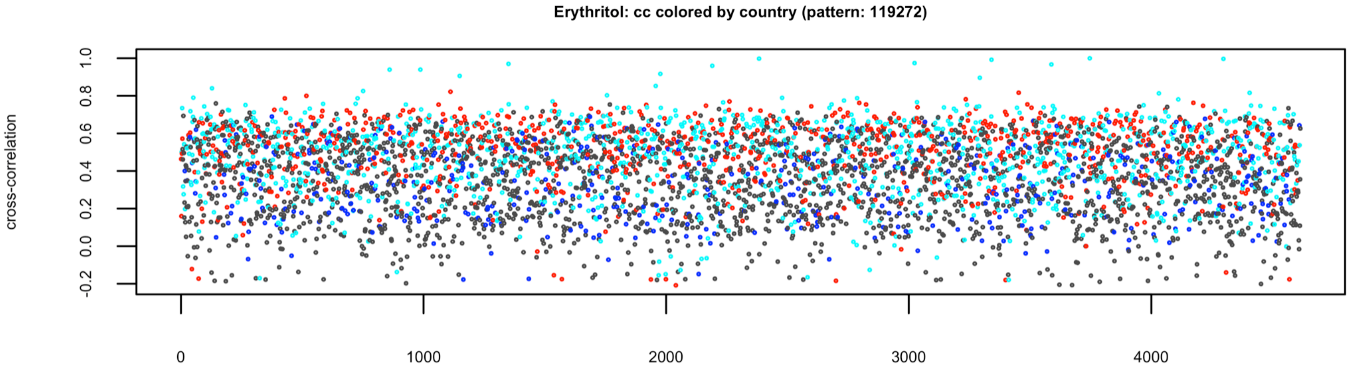

Supplementary Figure 13C: NMR spectra in the dataset showing erythritol pattern with high cross correlation threshold (CC) value > 0.9 (in green), intermediate CC between 0.78 to 0.9 (in amber) and low threshold showing no feature at CC < 0.78 (in red). We have presented 6 randomly selected spectra in each category. Note, users may choose to output more spectra within the COMPASS framework.

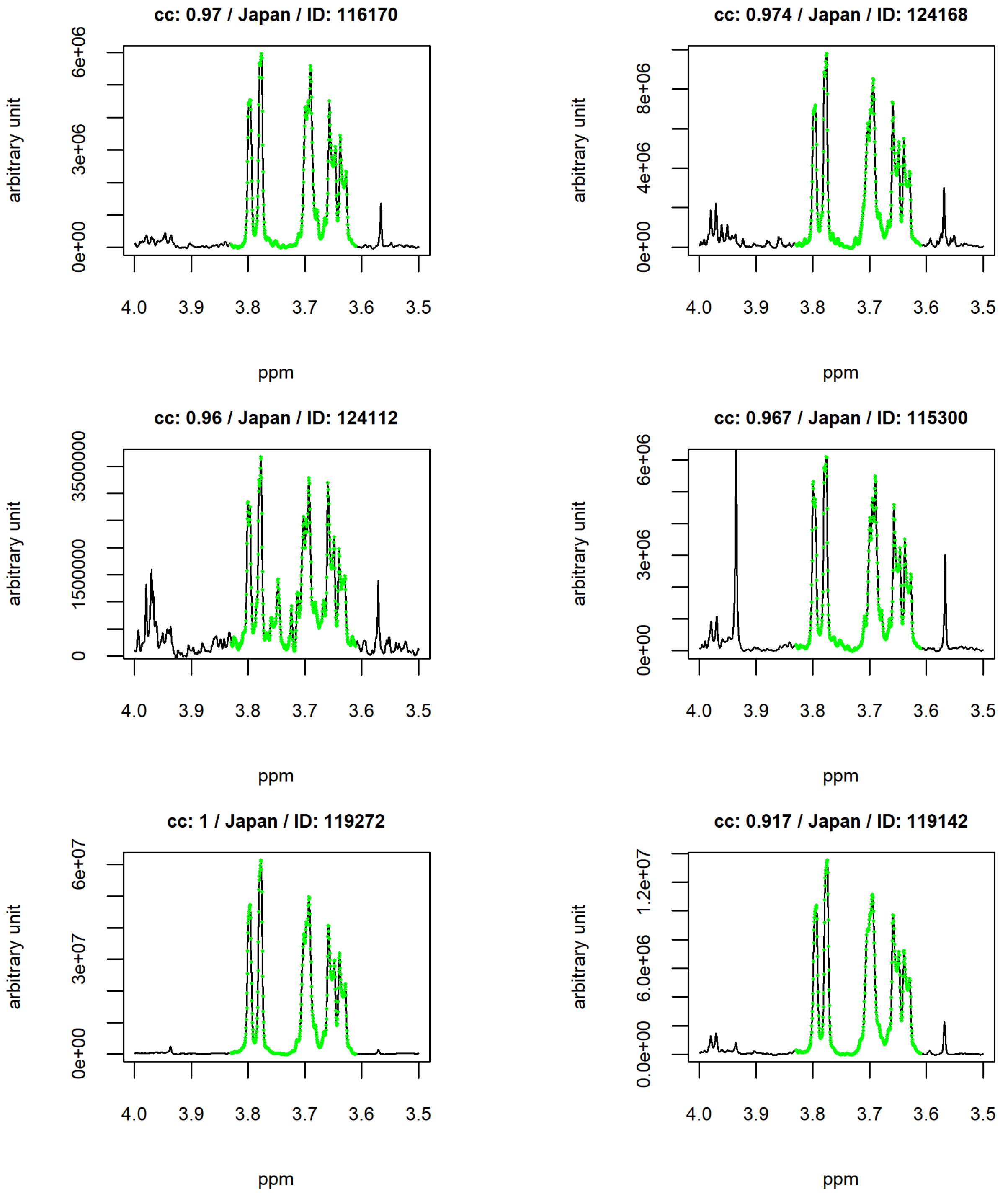

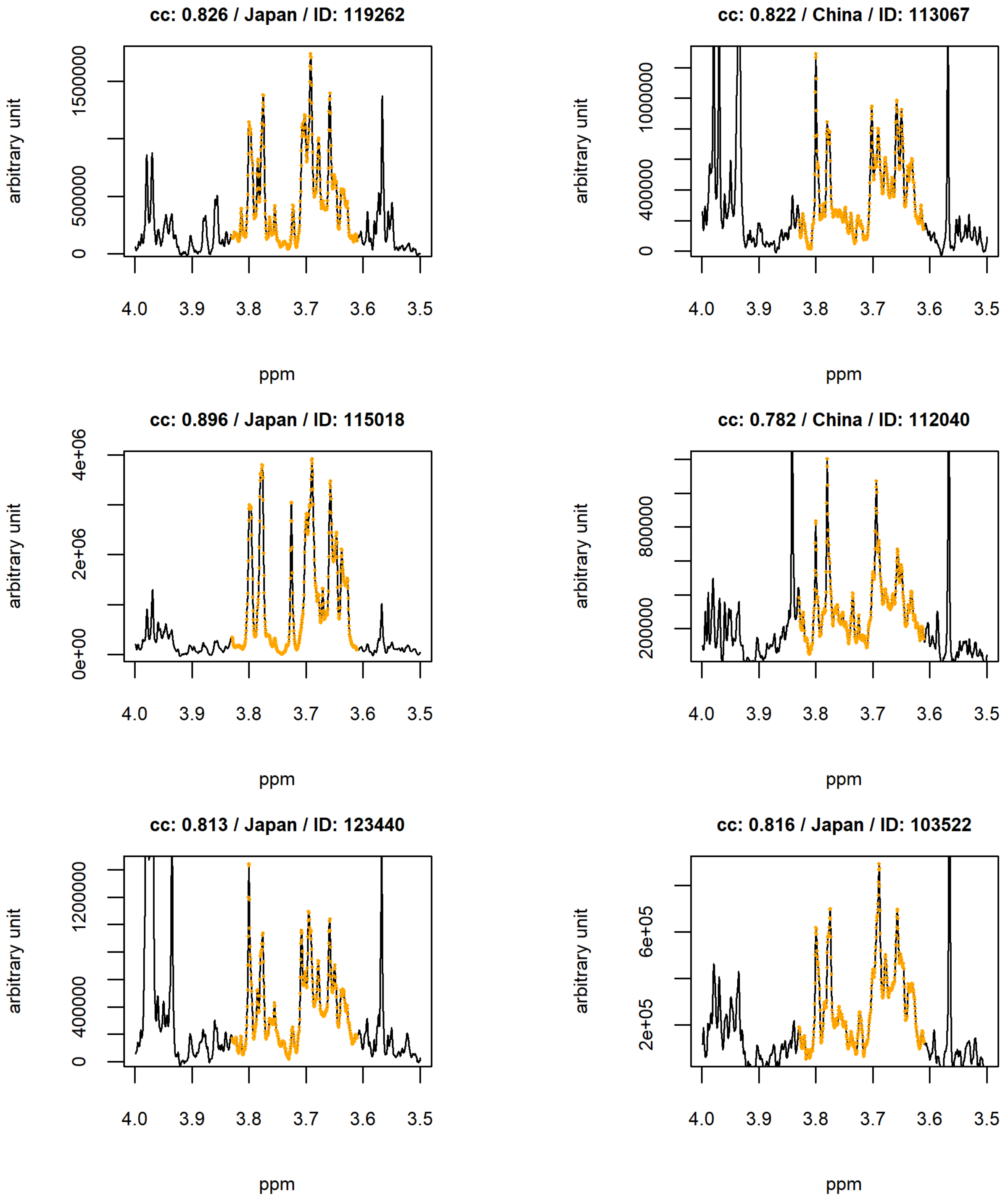
cc: 0.826 / Japan / ID: 119262
cc: 0.822 / China / ID: 113067
cc: 0.896 / Japan / ID: 115018
cc: 0.782 / China / ID: 112040
cc: 0.813 / Japan / ID: 123440
cc: 0.816 / Japan / ID: 103522
arbitrary unit
ppm
4.0 3.9 3.8 3.7 3.6 3.5
0 500000 1500000
0 400000 1000000
0e+00 2e+06 4e+06
200000 800000
0 400000 1200000
2e+05 6e+05

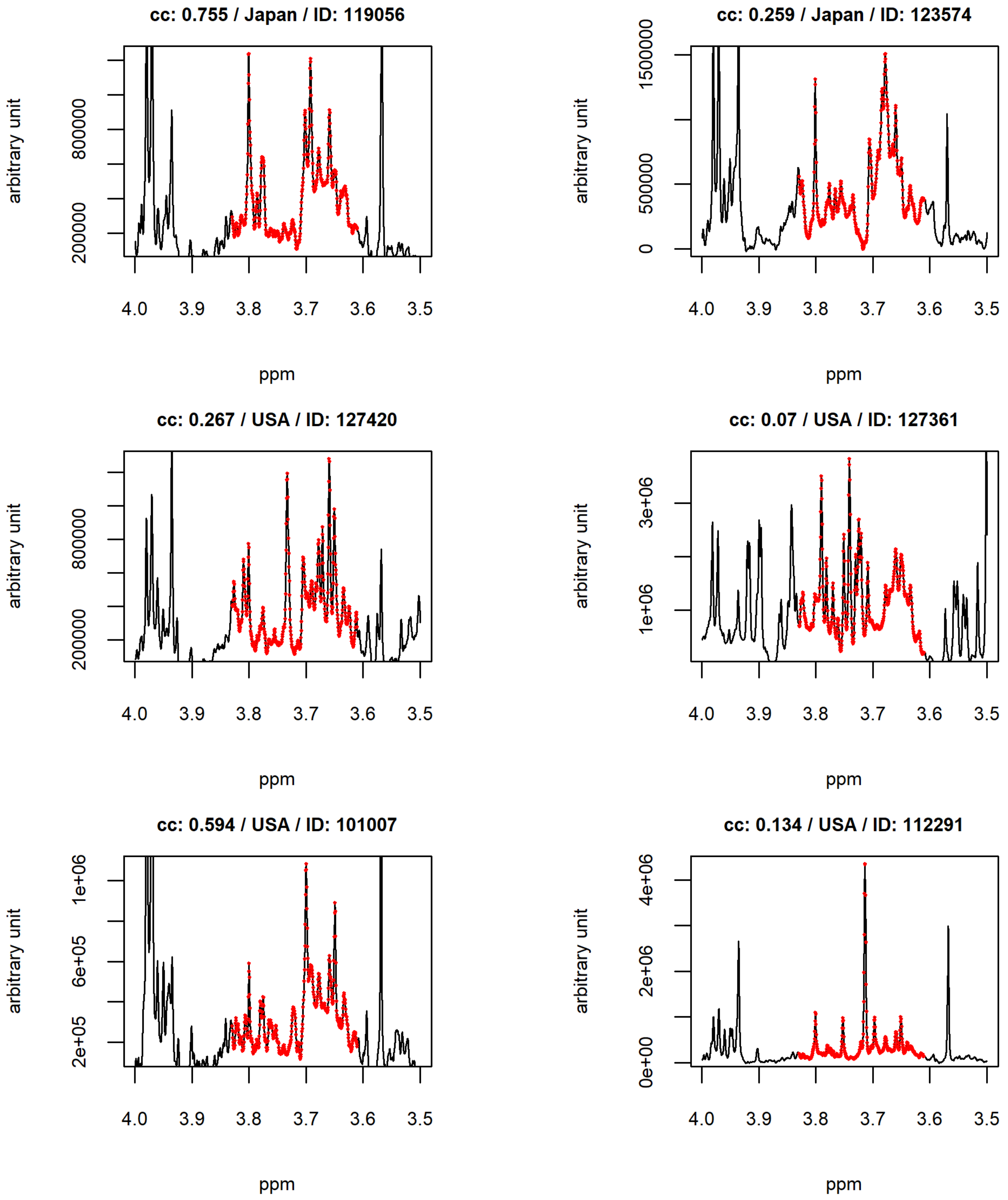
cc: 0.755 / Japan / ID: 119056
cc: 0.259 / Japan / ID: 123574
cc: 0.267 / USA / ID: 127420
cc: 0.07 / USA / ID: 127361
cc: 0.594 / USA / ID: 101007
cc: 0.134 / USA / ID: 112291
arbitrary unit
ppm
4.0
3.9
3.8
3.7
3.6
3.5
200000
800000
0
500000
1500000
1e+06
3e+06
2e+05
6e+05
1e+06
0e+00
2e+06
4e+06

Supplementary Table 13: Population statistics for erythritol using COMPASS approach

Percentage of samples with erythritol metabolite in the urine and by country

```
##
## China Japan    UK   USA
##   0.6   2.1   0.0   0.0
```

Total number of samples with erythritol metabolite in the urine and by country

```
##
## China Japan    UK   USA
##     5    24     0     0
```

Total number of samples with erythritol metabolite in the urine

```
## [1] 29
```
