# Supplementary material for: Strategy for improved characterization of human metabolic phenotypes using a COmbined Multi-block Principal components Analysis with Statistical Spectroscopy (COMPASS)
Source: Bioinformatics. 2020 Jul 21;36(21):5229–36. doi: 10.1093/bioinformatics/btaa649 (PMC7850059; doi:10.1093/bioinformatics/btaa649)
Supplement: btaa649_Supplementary_Data [file btaa649_supplementary_data.zip › Supp 14_BAIB.pdf]

Supplementary Material 14: Typical output for COMPASS approach as illustrated using beta aminoisobutyric acid

Supplementary Figure 14A: Robust reference pattern of beta aminoisobutyric acid as identified using STOCSY.

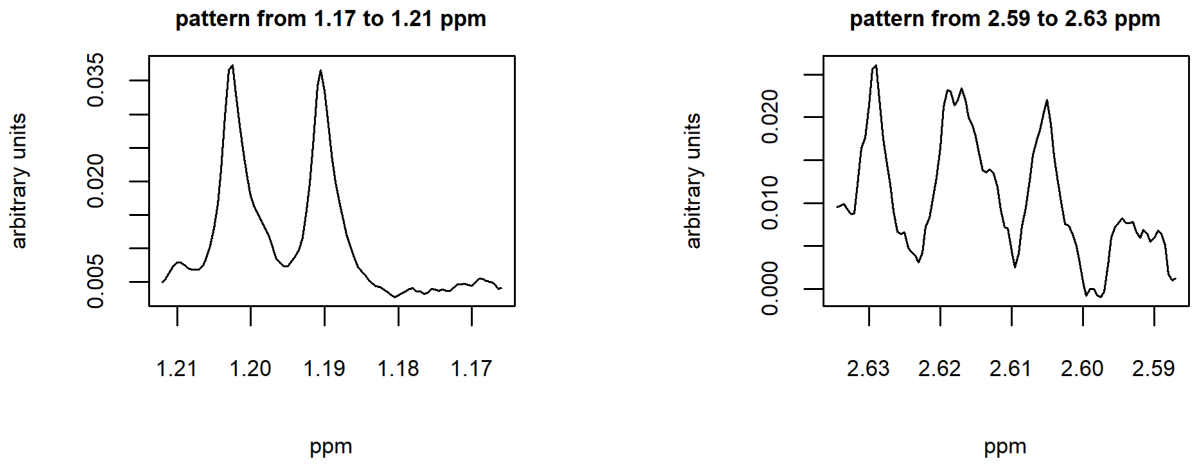

Supplementary Figure 14B: Distribution of cross-correlation using robust reference pattern of beta aminoisobutyric acid as shown in Supplementary Figure 14A and color coded to countries: China (red), Japan (turquoise), UK (blue), and USA (grey).

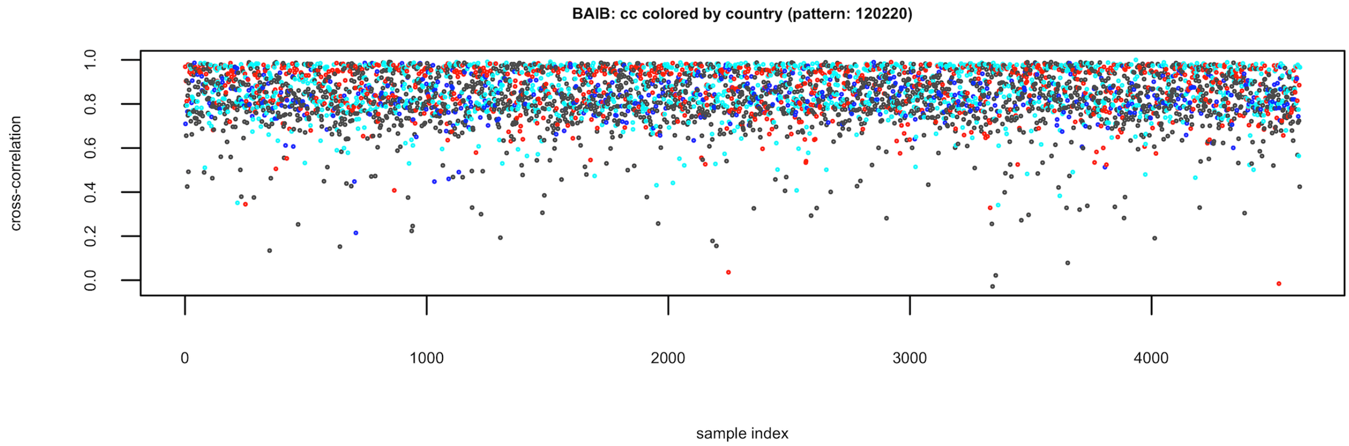

Supplementary Figure 14C: NMR spectra in the dataset showing beta aminoisobutyric acid pattern at 1.17 -1.21 ppm and 2.59 -2.63 ppm with high cross correlation threshold (CC) value > 0.95 (in green), intermediate CC between 0.9 to 0.95 (in amber) and low threshold showing no feature at CC < 0.95 (in red). We have presented 6 randomly selected spectra in each category. Note, users may choose to output more spectra within the COMPASS framework.

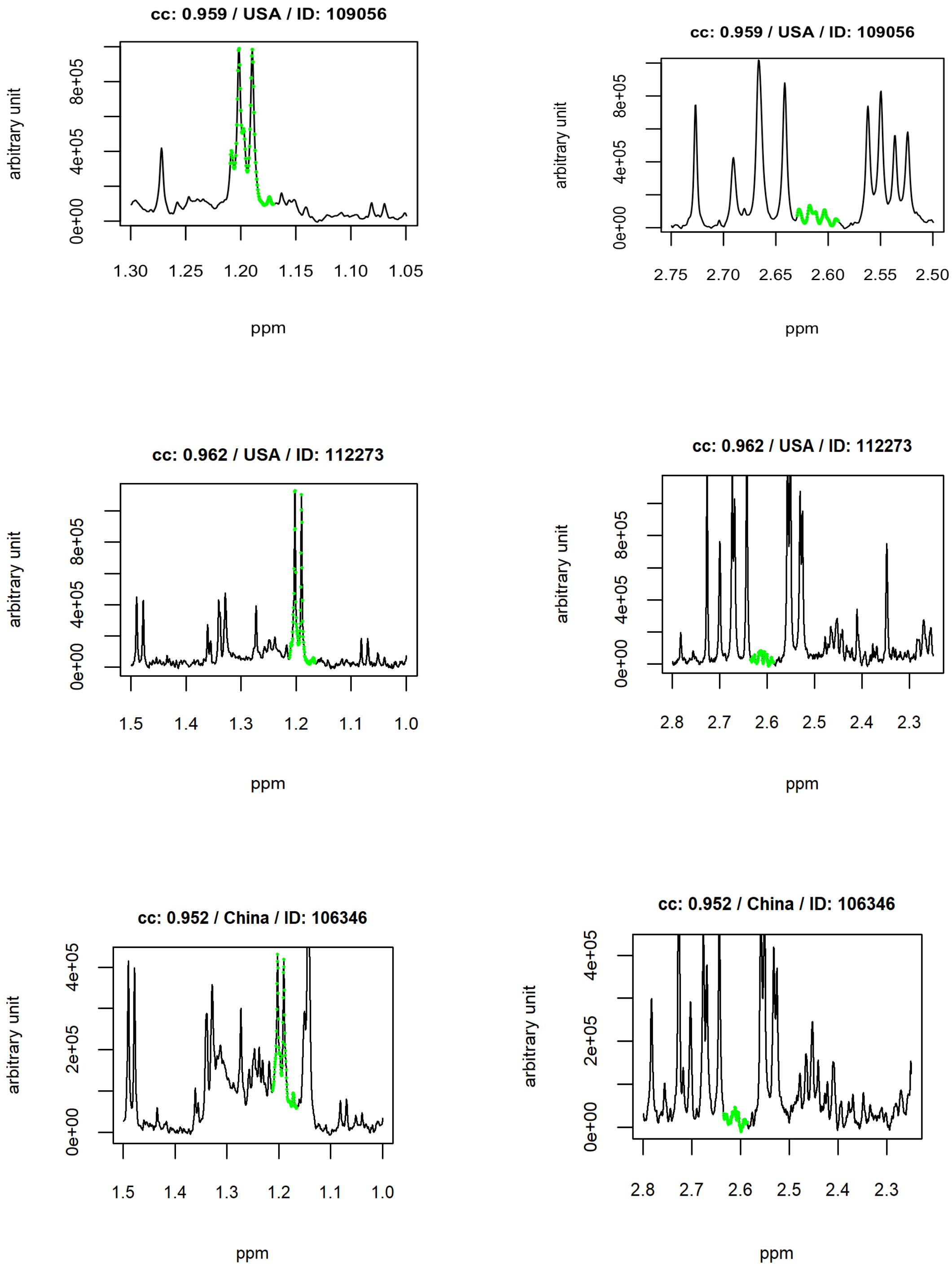

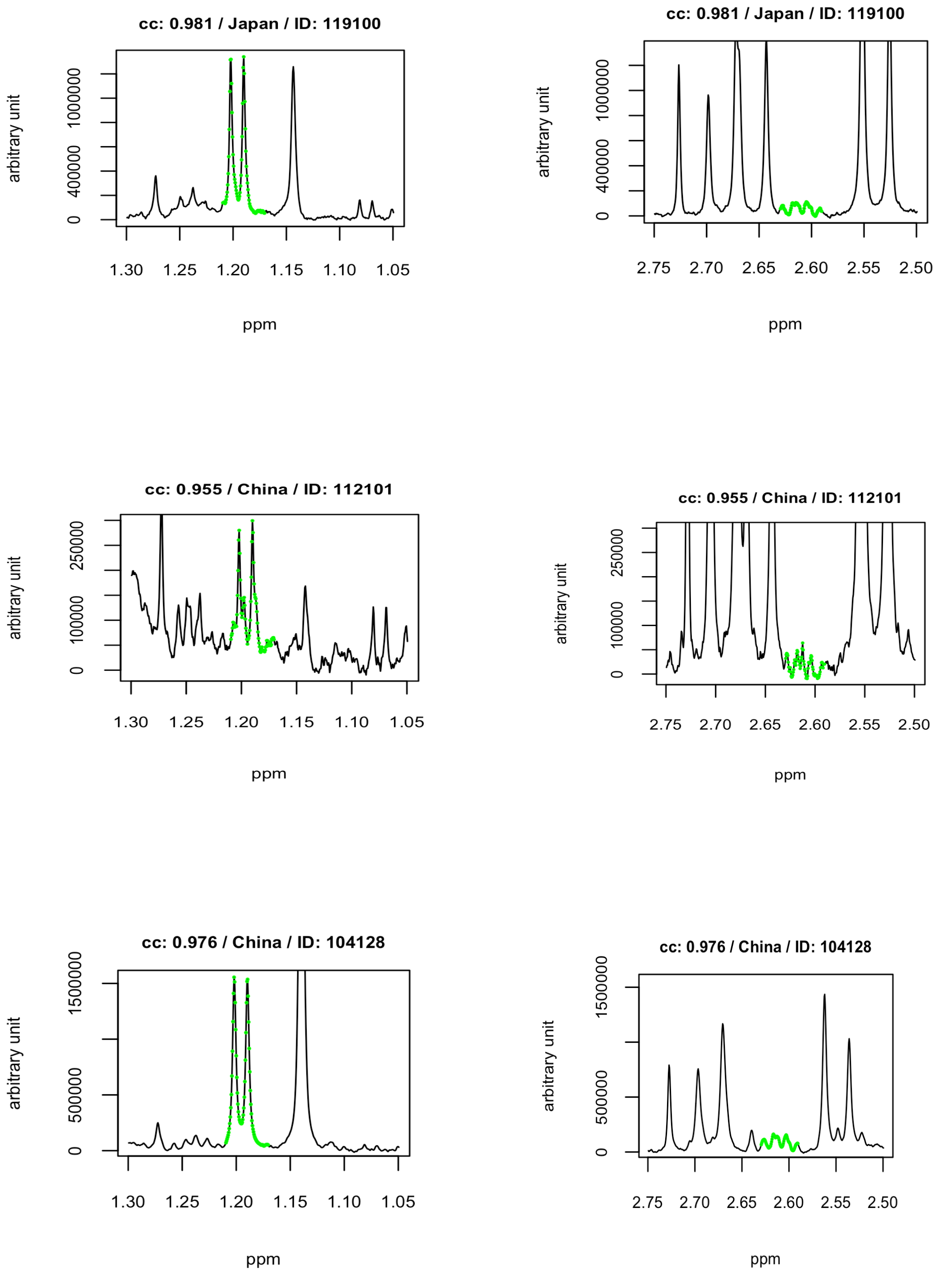
cc: 0.981 / Japan / ID: 119100
arbitrary unit
0
400000
1000000
1.30 1.25 1.20 1.15 1.10 1.05
ppm
cc: 0.981 / Japan / ID: 119100
arbitrary unit
0
400000
1000000
2.75 2.70 2.65 2.60 2.55 2.50
ppm
cc: 0.955 / China / ID: 112101
arbitrary unit
0
100000
250000
1.30 1.25 1.20 1.15 1.10 1.05
ppm
cc: 0.955 / China / ID: 112101
arbitrary unit
0
100000
250000
2.75 2.70 2.65 2.60 2.55 2.50
ppm
cc: 0.976 / China / ID: 104128
arbitrary unit
0
500000
1500000
1.30 1.25 1.20 1.15 1.10 1.05
ppm
cc: 0.976 / China / ID: 104128
arbitrary unit
0
500000
1500000
2.75 2.70 2.65 2.60 2.55 2.50
ppm

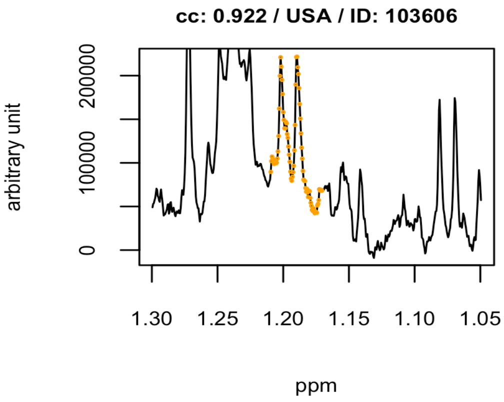
cc: 0.922 / USA / ID: 103606
arbitrary unit
0
100000
200000
1.30
1.25
1.20
1.15
1.10
1.05
ppm

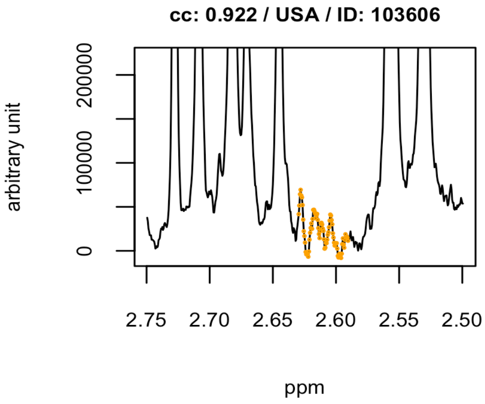
cc: 0.922 / USA / ID: 103606
arbitrary unit
0
100000
200000
2.75
2.70
2.65
2.60
2.55
2.50
ppm

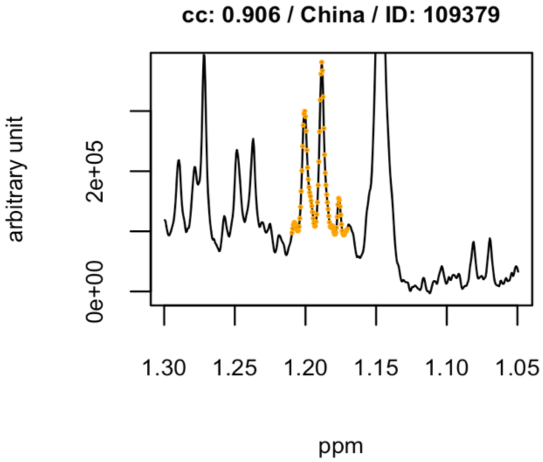
cc: 0.906 / China / ID: 109379
arbitrary unit
0e+00
2e+05
1.30
1.25
1.20
1.15
1.10
1.05
ppm

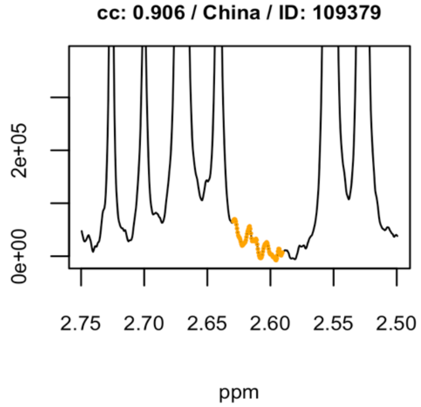
cc: 0.906 / China / ID: 109379
0e+00
2e+05
2.75
2.70
2.65
2.60
2.55
2.50
ppm

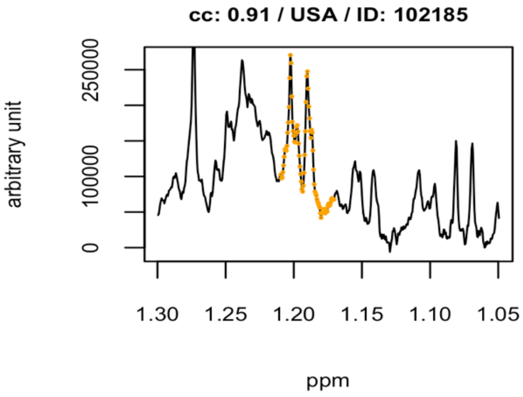
cc: 0.91 / USA / ID: 102185
arbitrary unit
0
100000
250000
1.30
1.25
1.20
1.15
1.10
1.05
ppm

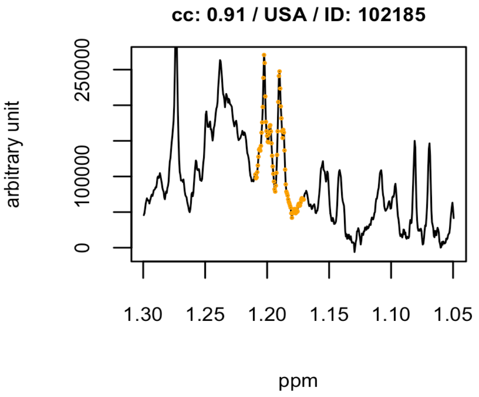
cc: 0.91 / USA / ID: 102185
arbitrary unit
0
100000
250000
1.30
1.25
1.20
1.15
1.10
1.05
ppm

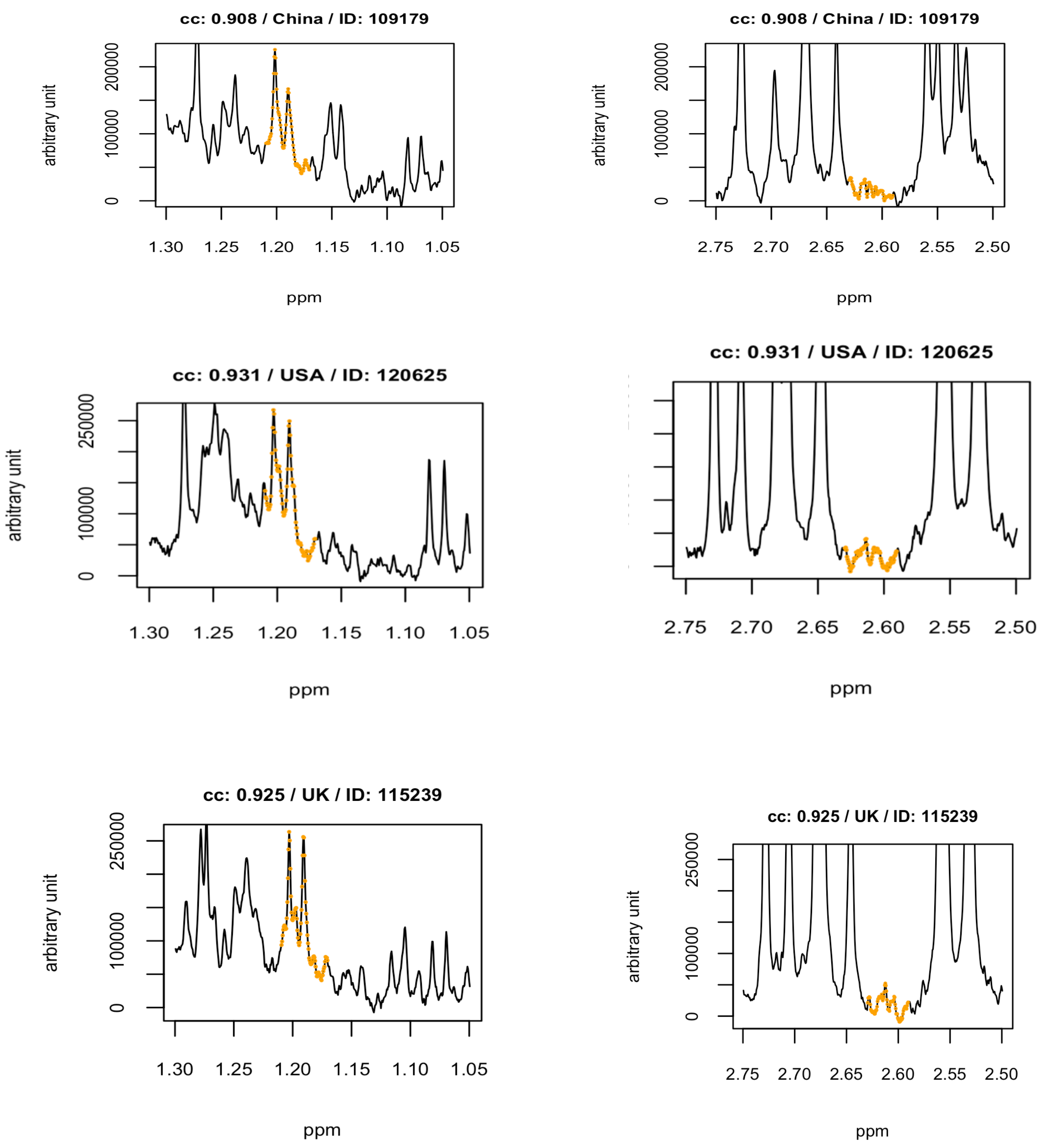
cc: 0.908 / China / ID: 109179
arbitrary unit
0
100000
200000
1.30 1.25 1.20 1.15 1.10 1.05
ppm
cc: 0.908 / China / ID: 109179
arbitrary unit
0
100000
200000
2.75 2.70 2.65 2.60 2.55 2.50
ppm
cc: 0.931 / USA / ID: 120625
arbitrary unit
0
100000
250000
1.30 1.25 1.20 1.15 1.10 1.05
ppm
cc: 0.931 / USA / ID: 120625
2.75 2.70 2.65 2.60 2.55 2.50
ppm
cc: 0.925 / UK / ID: 115239
arbitrary unit
0
100000
250000
1.30 1.25 1.20 1.15 1.10 1.05
ppm
cc: 0.925 / UK / ID: 115239
arbitrary unit
0
100000
250000
2.75 2.70 2.65 2.60 2.55 2.50
ppm

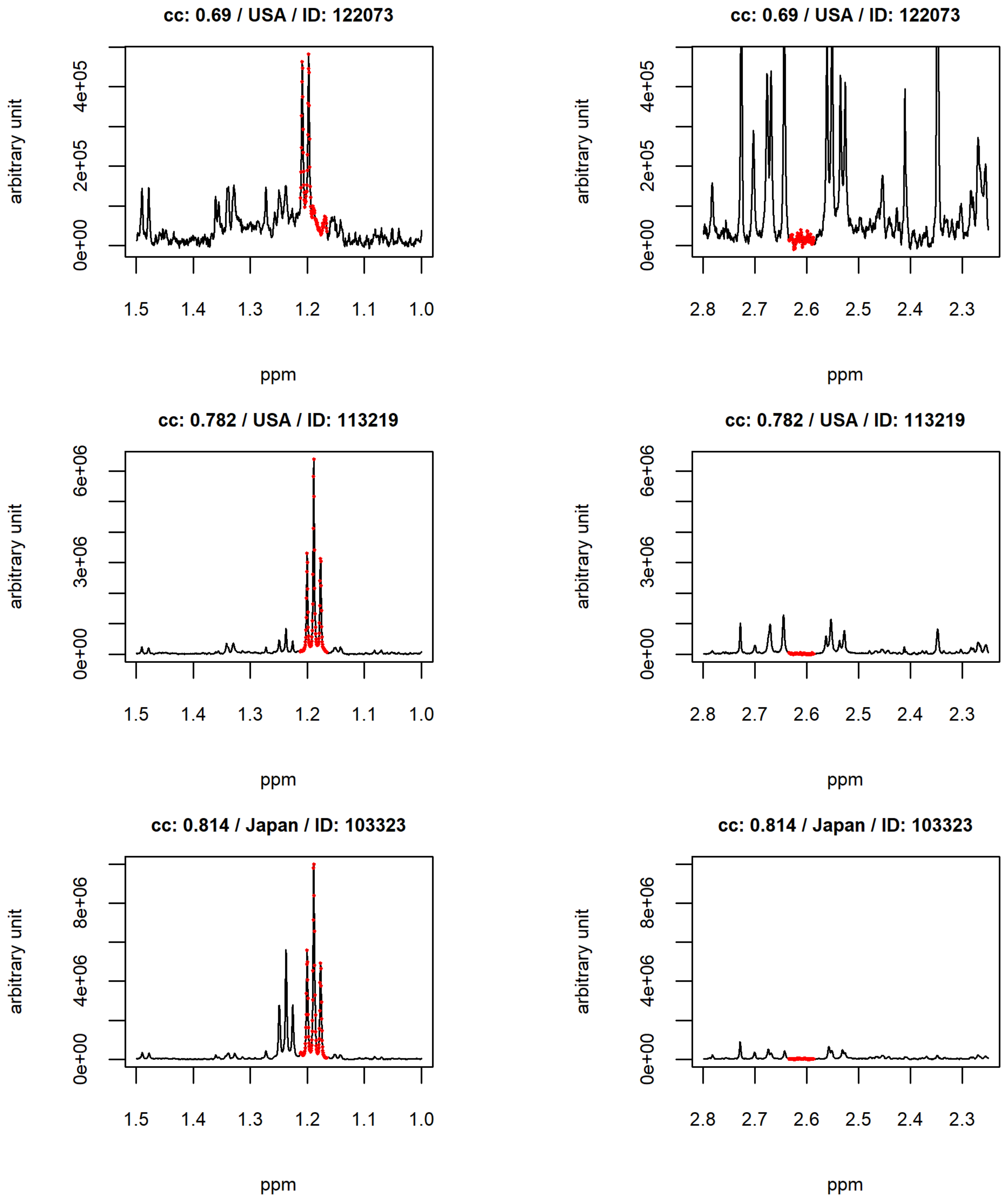
cc: 0.69 / USA / ID: 122073
arbitrary unit
0e+00
2e+05
4e+05
1.5
1.4
1.3
1.2
1.1
1.0
ppm
cc: 0.69 / USA / ID: 122073
arbitrary unit
0e+00
2e+05
4e+05
2.8
2.7
2.6
2.5
2.4
2.3
ppm
cc: 0.782 / USA / ID: 113219
arbitrary unit
0e+00
3e+06
6e+06
1.5
1.4
1.3
1.2
1.1
1.0
ppm
cc: 0.782 / USA / ID: 113219
arbitrary unit
0e+00
3e+06
6e+06
2.8
2.7
2.6
2.5
2.4
2.3
ppm
cc: 0.814 / Japan / ID: 103323
arbitrary unit
0e+00
4e+06
8e+06
1.5
1.4
1.3
1.2
1.1
1.0
ppm
cc: 0.814 / Japan / ID: 103323
arbitrary unit
0e+00
4e+06
8e+06
2.8
2.7
2.6
2.5
2.4
2.3
ppm

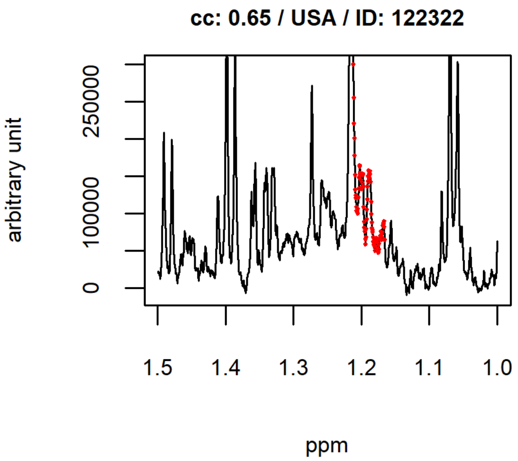
cc: 0.65 / USA / ID: 122322
arbitrary unit
0
100000
250000
1.5
1.4
1.3
1.2
1.1
1.0
ppm

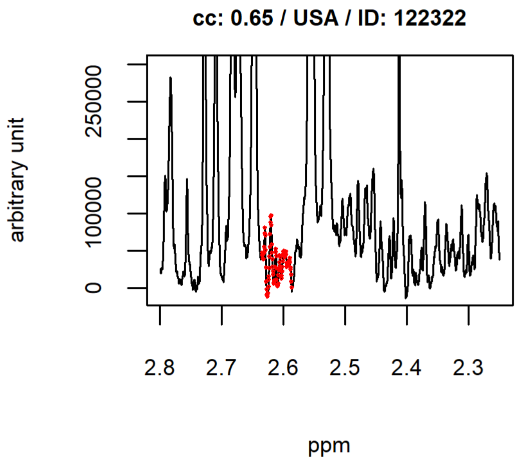
cc: 0.65 / USA / ID: 122322
arbitrary unit
0
100000
250000
2.8
2.7
2.6
2.5
2.4
2.3
ppm

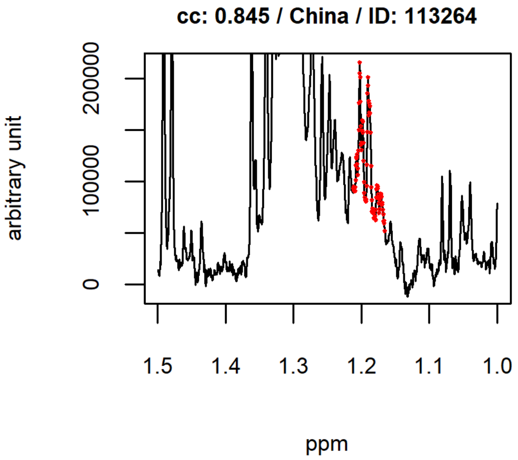
cc: 0.845 / China / ID: 113264
arbitrary unit
0
100000
200000
1.5
1.4
1.3
1.2
1.1
1.0
ppm

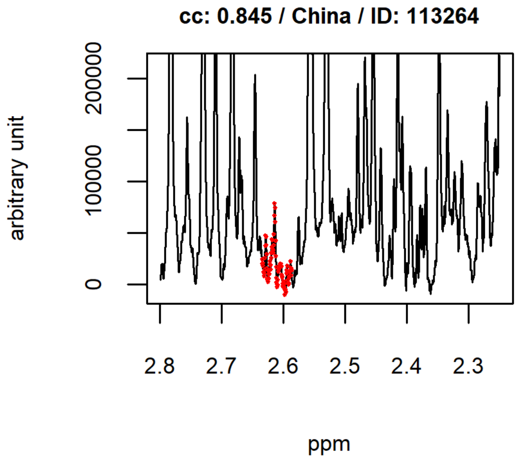
cc: 0.845 / China / ID: 113264
arbitrary unit
0
100000
200000
2.8
2.7
2.6
2.5
2.4
2.3
ppm

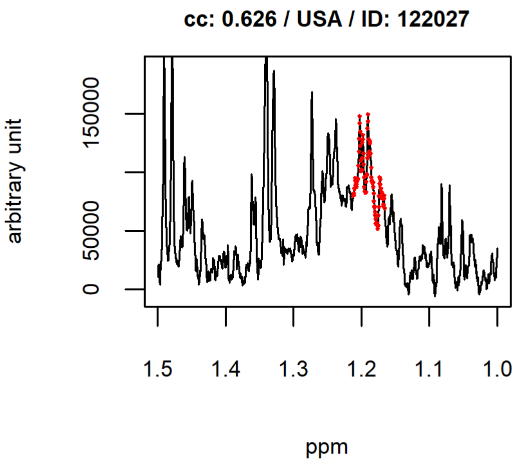
cc: 0.626 / USA / ID: 122027
arbitrary unit
0
50000
150000
1.5
1.4
1.3
1.2
1.1
1.0
ppm

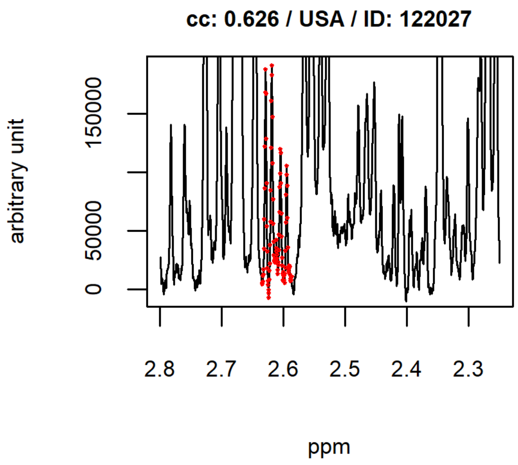
cc: 0.626 / USA / ID: 122027
arbitrary unit
0
50000
150000
2.8
2.7
2.6
2.5
2.4
2.3
ppm

Supplementary Table 14: Population statistics for beta aminoisobutyric acid using COMPASS approach

Percentage of samples with BAIB in the urine and by country

```
##
## China Japan    UK   USA
##  50.2   40.9      21.8  25.6
```

Total number of samples with BAIB in the urine and by country

```
##
## China Japan    UK   USA
##   415   465      108   552
```

Total number of samples with BAIB in the urine

```
## [1] 1540
```
