# Supplementary material for: Strategy for improved characterization of human metabolic phenotypes using a COmbined Multi-block Principal components Analysis with Statistical Spectroscopy (COMPASS)
Source: Bioinformatics. 2020 Jul 21;36(21):5229–36. doi: 10.1093/bioinformatics/btaa649 (PMC7850059; doi:10.1093/bioinformatics/btaa649)
Supplement: btaa649_Supplementary_Data [file btaa649_supplementary_data.zip › Supp 6_AnimalDatasetDescription.docx]

**Supplementary Material 6: Description of the animal surgical dataset**

This dataset involves 12 male Wistar rats (non-obese) that were individually housed under a 12 h/12 h light/dark cycle at a room temperature of 21 ± 2 °C^1, 2^. Water and standard chow were available ad libitum. All experiments were performed under a licence issued by the UK Home Office (PL 70-6669). Rats were acclimatised for 1 week and randomised to RYGB or sham operation (n=6 per group). Urine specimens were collected for 24 hours before surgical procedure (pre), and at 2 weeks (W2), 6 weeks (W6) and 8 weeks (W8) post operation. All specimens were d stored at −80° C

**^1^H NMR spectroscopic analysis:** Urine samples were thoroughly defrosted and vortexed for 15 s prior to mixing 400 μl of urine with 250 μl of 0.2 M phosphate buffer (pH=7.4) containing 20% deuterium oxide (D2O) for the magnetic field lock, 0.01% 3-(trimethylsilyl)-[2,2,3,3-^2^H_4_]-propionic acid sodium salt (TSP) for the spectral calibration and 3 mM sodium azide (Na_3_N) for avoiding bacterial contamination. The resulting mixture was centrifuged at 10,392 g for 10 min and 600 μl of supernatants was transferred into a NMR tube with a diameter of 5 mm for ^1^H NMR spectral acquisition using a Bruker 600 MHz spectrometer (Bruker; Rheinstetten, Germany) at the operating ^1^H frequency of 600.13 MHz with a temperature of 300 K. A standard NMR pulse sequence (recycle delay [RD]−90°- t_1_-90°- t_m_-90°-acquisition) was applied to acquire 1-dimensional (1-D) ^1^H NMR spectral data, where t_1_ was set to 3 μs and tm (mixing time) was set to 100 ms. The water peak suppression was achieved using selective irradiation during RD of 2 s and t_m_. A 90 degree pulse was adjusted to approximately 10 μs. A total of 128 scans were collected into 64 k data points with a spectral width of 20 ppm. ^1^H NMR spectra obtained from urine extracts were automatically phased, referenced and baseline-corrected using a in-house MATLAB script developed by Dr. T. Ebbels at Imperial College. The resulting NMR spectra (δ0-10) were imported to MATLAB software and digitized into 20 k data points with the resolution of 0.0005 using script developed in house (Dr. O. Cloarec). The following regions were removed: water peak region at δ 4.62-5.05; urea region at δ 5.47-6.24 and noise region at δ 0-0.10 and δ 9.90-10.00 prior to normalization.

1. Li JV, Ashrafian H, Bueter M, Kinross J, Sands C, le Roux CW *et al* Metabolic surgery profoundly influences gut microbial-host metabolic cross-talk. Gut 2011a; 60: 1214-1223.

2. Li JV, Reshat R, Wu Q, Ashrafian H, Bueter M, le Roux CW *et al* Experimental bariatric surgery in rats generates a cytotoxic chemical environment in the gut contents. Front Microbiol 2011b; 2: 183.
