# Supplementary material for: Strategy for improved characterization of human metabolic phenotypes using a COmbined Multi-block Principal components Analysis with Statistical Spectroscopy (COMPASS)
Source: Bioinformatics. 2020 Jul 21;36(21):5229–36. doi: 10.1093/bioinformatics/btaa649 (PMC7850059; doi:10.1093/bioinformatics/btaa649)
Supplement: btaa649_Supplementary_Data [file btaa649_supplementary_data.zip › Supp 7_Ibuprofen.pdf]

Supplementary Figure 7A: Visual inspection of 17 spectra that are deemed to contain ibuprofen metabolites based on a CC threshold of 0.70 but was not predicted as containing ibuprofen metabolites in previous publication by Loo *et al*. 2012. Visual inspection of these spectra, which are displayed using traffic light display system (green for CC >0.85 and amber for CC between 0.7 -0.85), showed all spectra contained ibuprofen metabolites resonances.

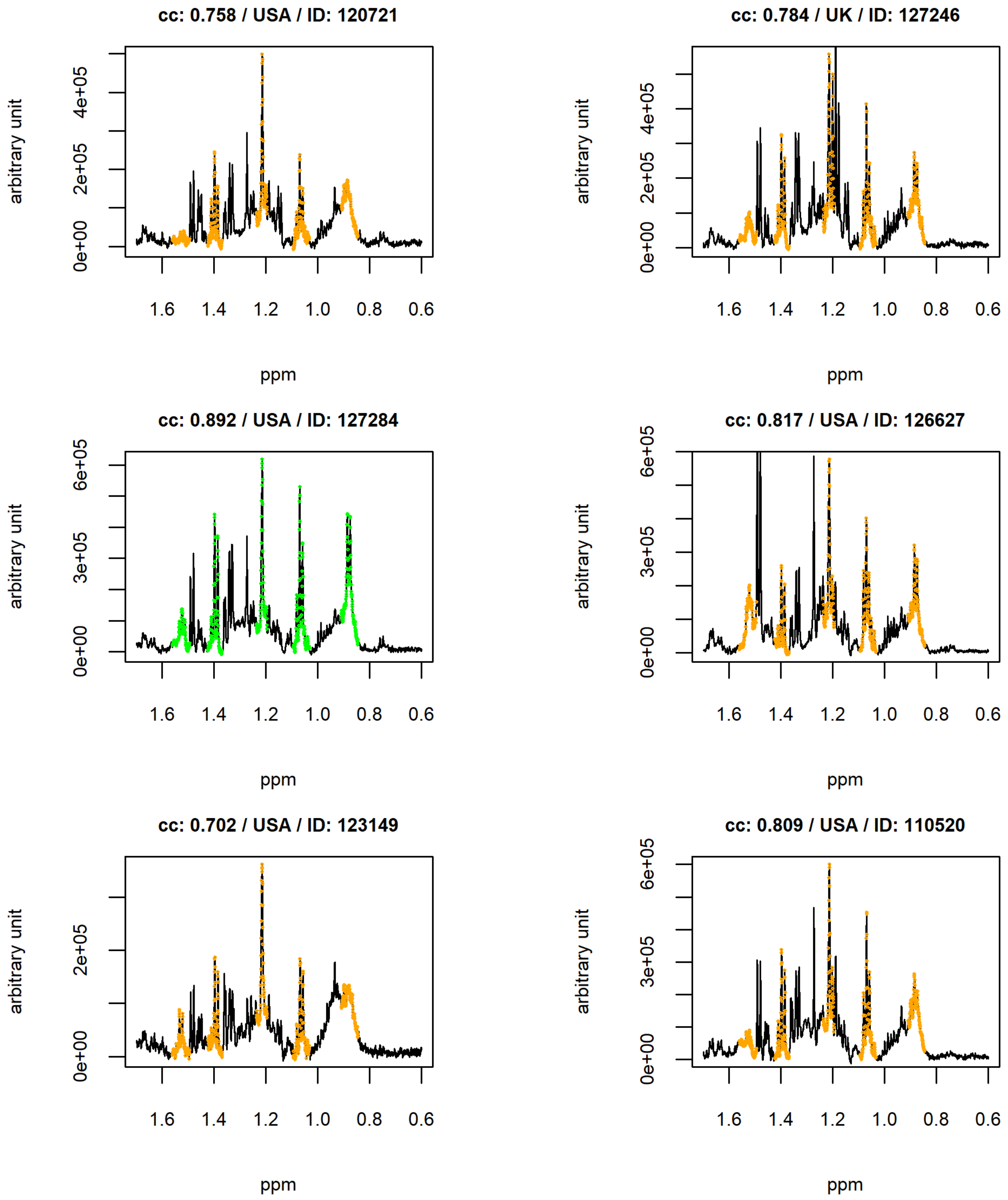

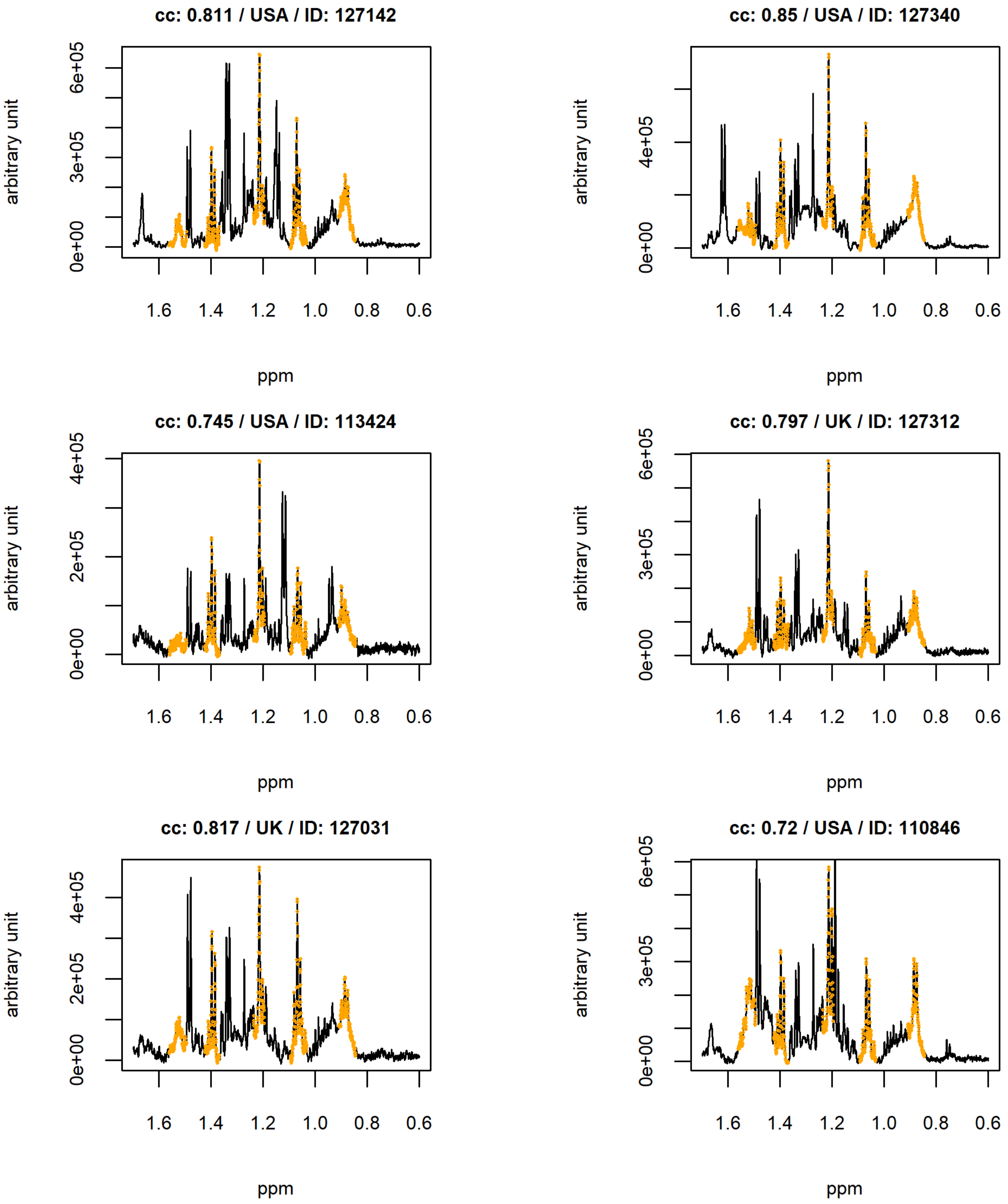

cc: 0.811 / USA / ID: 127142
cc: 0.85 / USA / ID: 127340
cc: 0.745 / USA / ID: 113424
cc: 0.797 / UK / ID: 127312
cc: 0.817 / UK / ID: 127031
cc: 0.72 / USA / ID: 110846
arbitrary unit
ppm
1.6 1.4 1.2 1.0 0.8 0.6
0e+00 3e+05 6e+05
0e+00 4e+05
0e+00 2e+05 4e+05

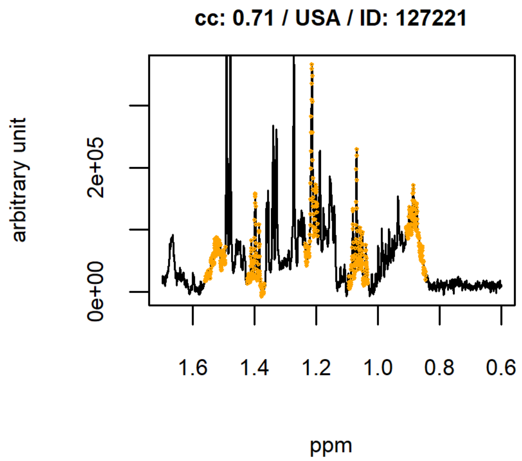
cc: 0.71 / USA / ID: 127221
arbitrary unit
0e+00
2e+05
1.6
1.4
1.2
1.0
0.8
0.6
ppm

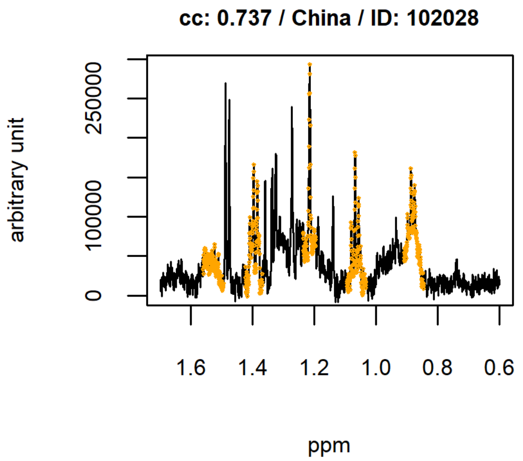
cc: 0.737 / China / ID: 102028
arbitrary unit
0
100000
250000
1.6
1.4
1.2
1.0
0.8
0.6
ppm

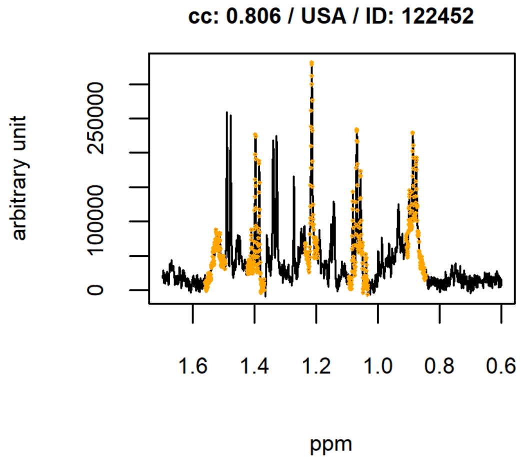
cc: 0.806 / USA / ID: 122452
arbitrary unit
0
100000
250000
1.6
1.4
1.2
1.0
0.8
0.6
ppm

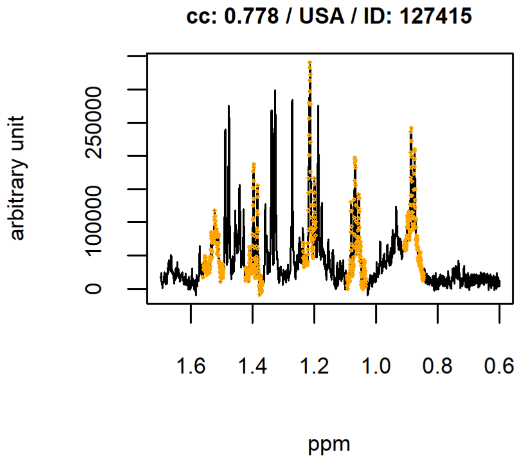
cc: 0.778 / USA / ID: 127415
arbitrary unit
0
100000
250000
1.6
1.4
1.2
1.0
0.8
0.6
ppm

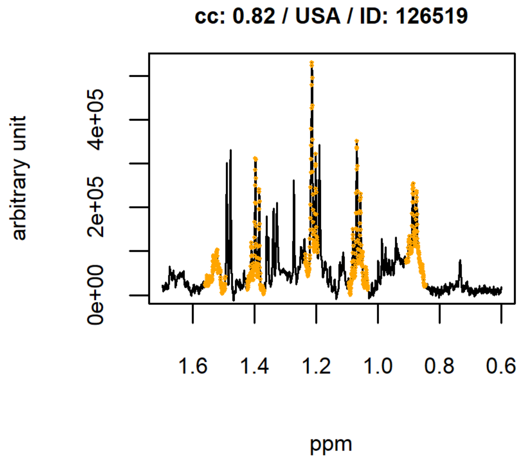
cc: 0.82 / USA / ID: 126519
arbitrary unit
0e+00
2e+05
4e+05
1.6
1.4
1.2
1.0
0.8
0.6
ppm

Supplementary Figure 7B: Visual inspection of 16 spectra that are deemed to contain ibuprofen metabolites based on previous publication by Loo *et al*. 2012 but were not considered to contain ibuprofen based on a CC threshold of 0.7. Visual inspection of these spectra showed ibuprofen metabolites resonances can be observed in all of these spectra except one spectrum with a CC of 0.063.

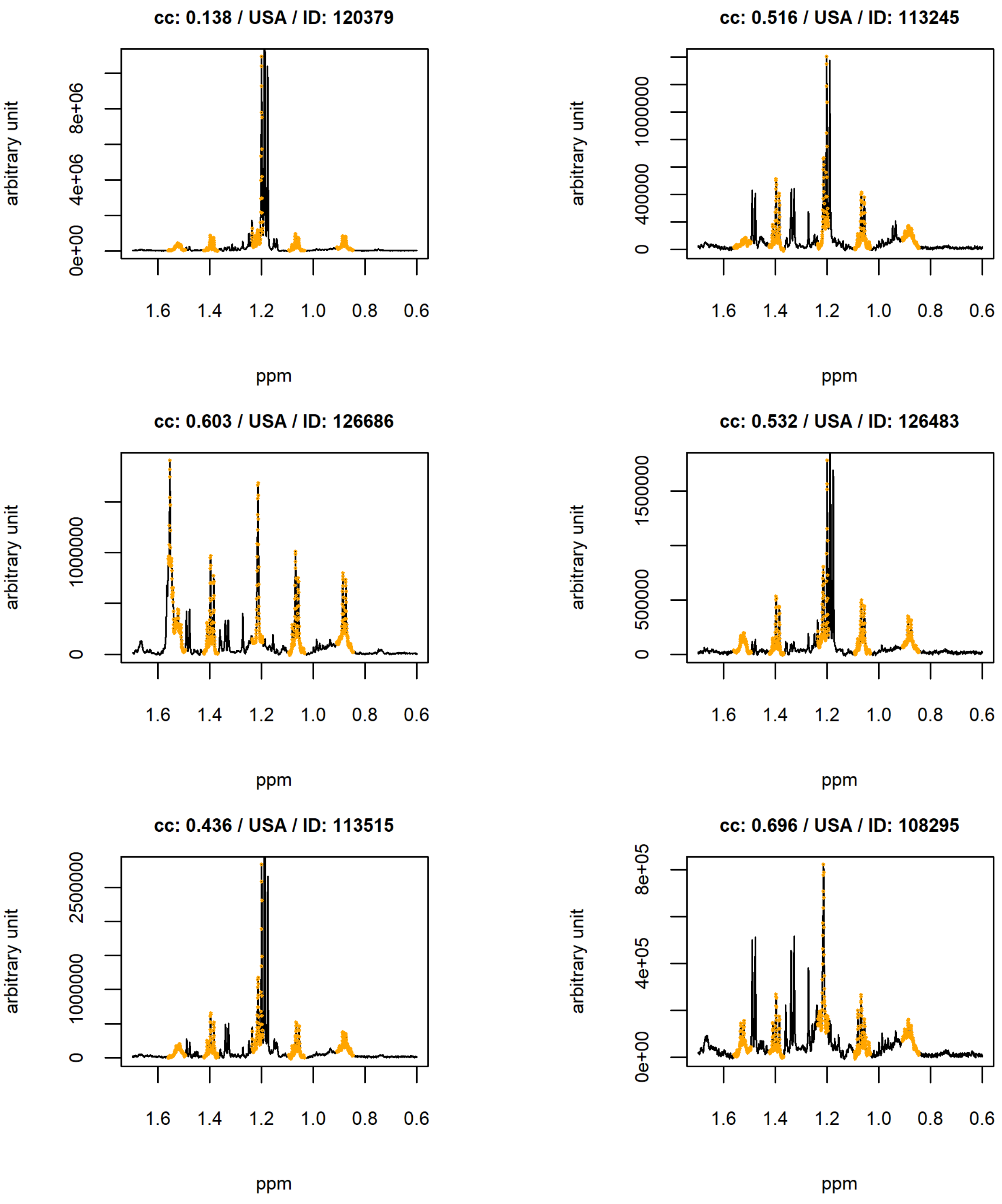

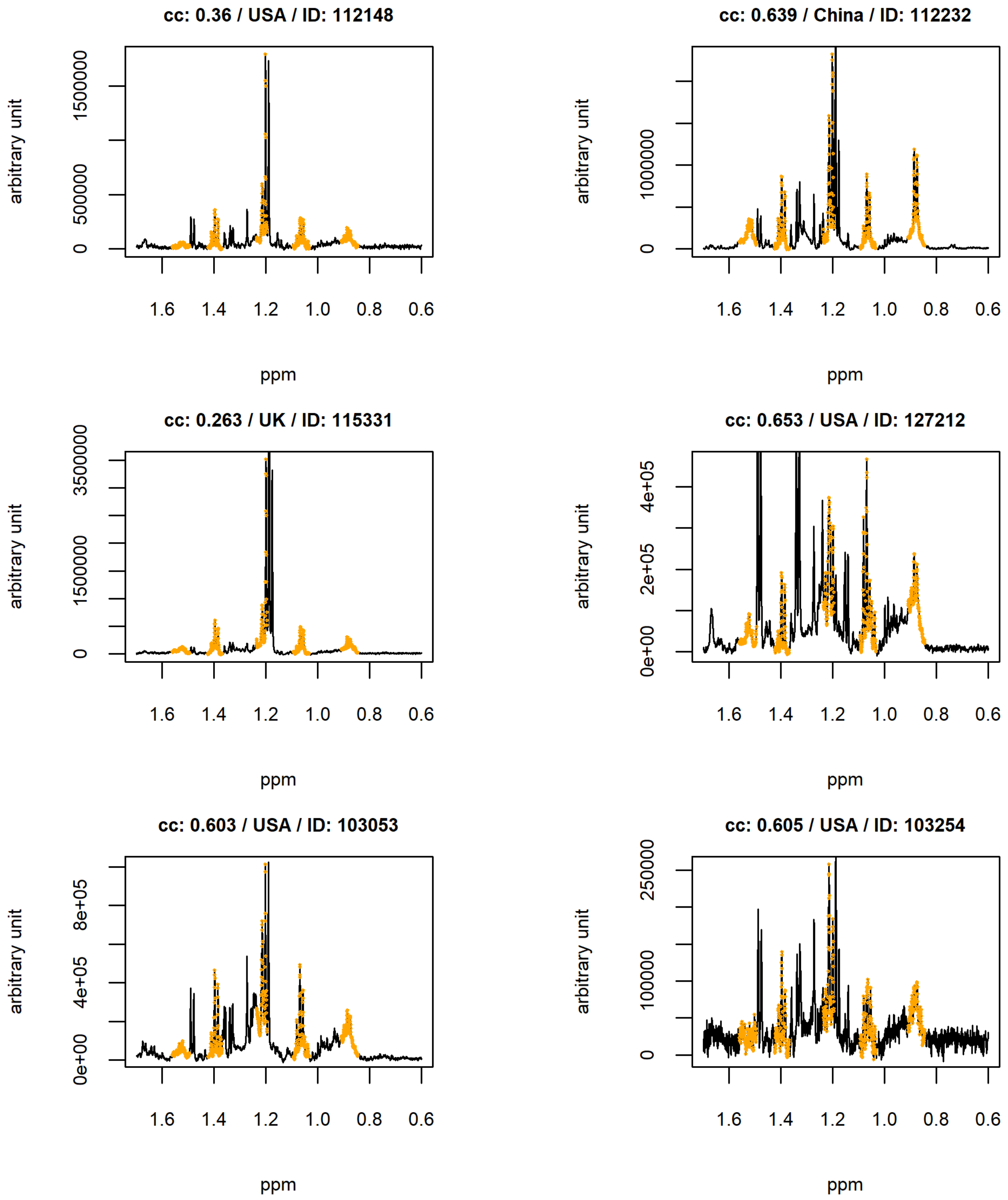
cc: 0.36 / USA / ID: 112148
cc: 0.639 / China / ID: 112232
cc: 0.263 / UK / ID: 115331
cc: 0.653 / USA / ID: 127212
cc: 0.603 / USA / ID: 103053
cc: 0.605 / USA / ID: 103254
arbitrary unit
ppm
0
500000
1500000
1000000
3500000
0e+00
2e+05
4e+05
8e+05
100000
250000
1.6
1.4
1.2
1.0
0.8
0.6

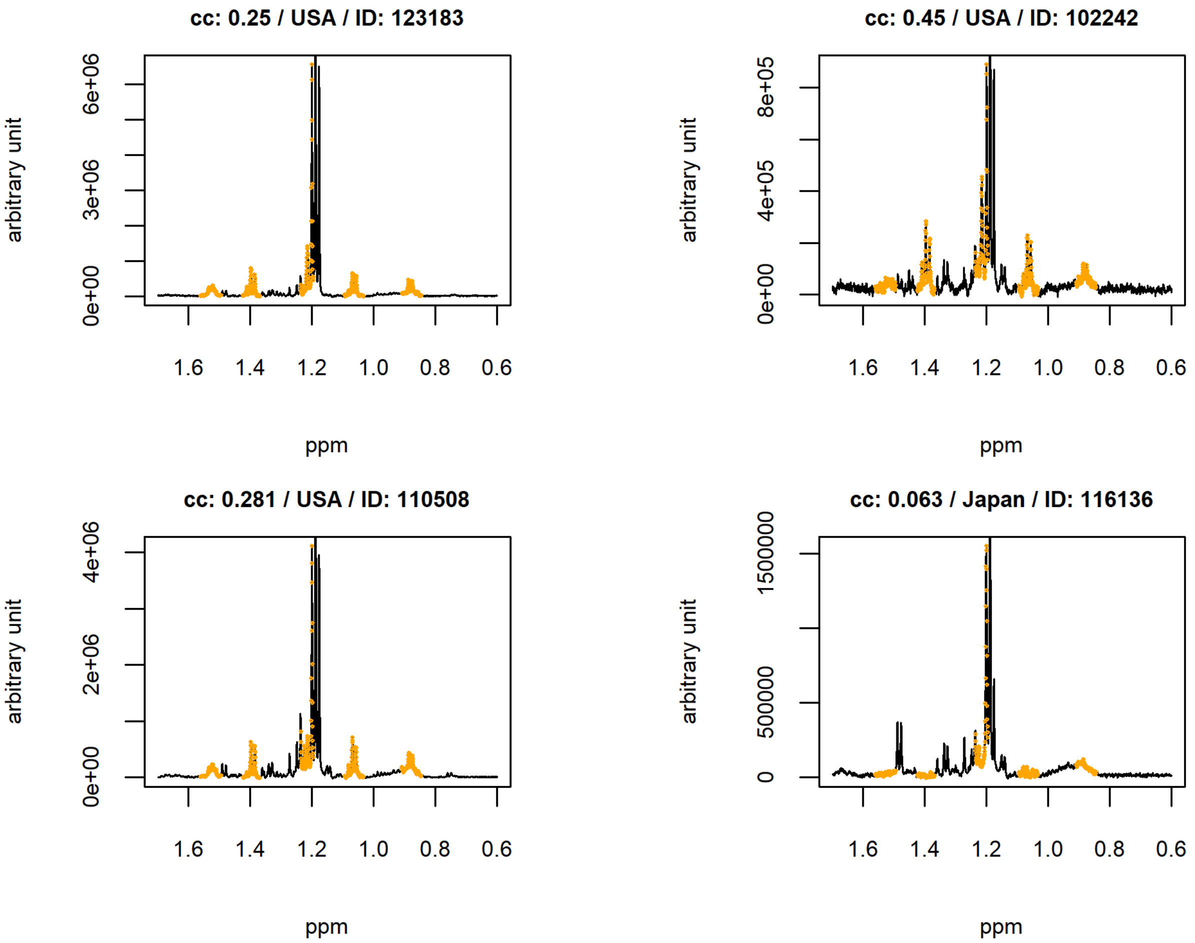
cc: 0.25 / USA / ID: 123183
arbitrary unit
0e+00
3e+06
6e+06
1.6
1.4
1.2
1.0
0.8
0.6
ppm
cc: 0.45 / USA / ID: 102242
arbitrary unit
0e+00
4e+05
8e+05
1.6
1.4
1.2
1.0
0.8
0.6
ppm
cc: 0.281 / USA / ID: 110508
arbitrary unit
0e+00
2e+06
4e+06
1.6
1.4
1.2
1.0
0.8
0.6
ppm
cc: 0.063 / Japan / ID: 116136
arbitrary unit
0
500000
1500000
1.6
1.4
1.2
1.0
0.8
0.6
ppm
