# Supplementary material for: Strategy for improved characterization of human metabolic phenotypes using a COmbined Multi-block Principal components Analysis with Statistical Spectroscopy (COMPASS)
Source: Bioinformatics. 2020 Jul 21;36(21):5229–36. doi: 10.1093/bioinformatics/btaa649 (PMC7850059; doi:10.1093/bioinformatics/btaa649)
Supplement: btaa649_Supplementary_Data [file btaa649_supplementary_data.zip › Supp 8_Acetaminophen.pdf]

Supplementary Material 8: Output of results for Acetaminophen based on COMPASS approach

Supplementary Figure 8A: A combination of major acetaminophen metabolites.

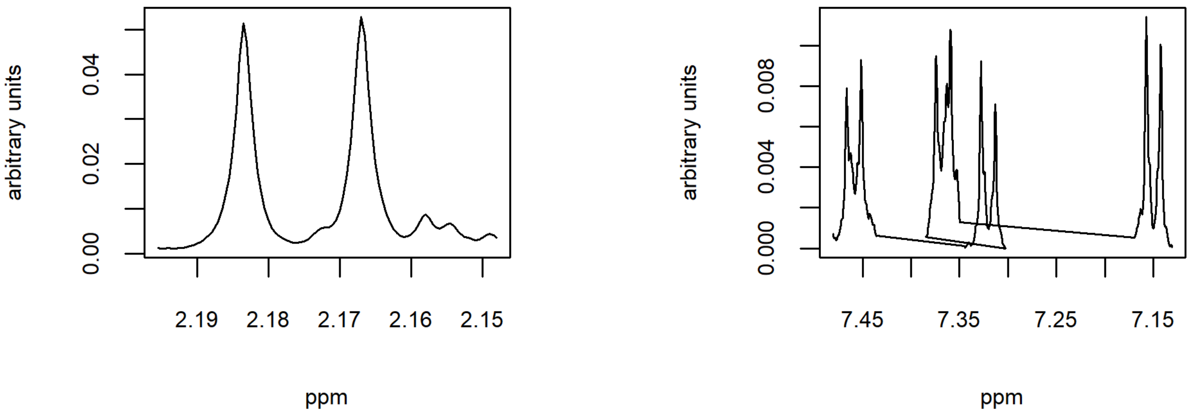

Supplementary Figure 8B: Distribution of cross-correlation using a combination of major acetaminophen metabolites as shown in Supplementary Figure 8A and color coded to countries: China (red), Japan (turquoise), UK (blue), and USA (grey).

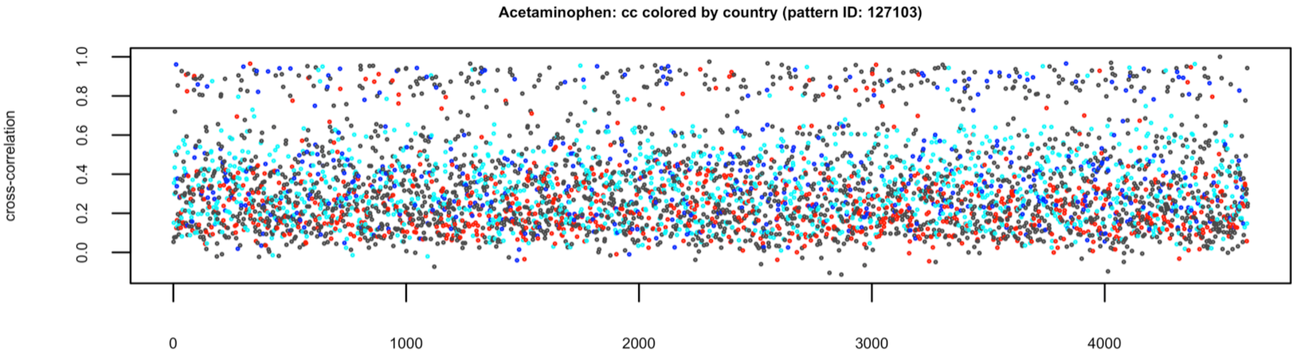

Supplementary Figure 8C: NMR spectra showing a combination of acetaminophen patterns (consist of 2.15 - 2.20, 7.13-7.16, 7.30-7.34, 7.34 -7.38 and 7.43 - 7.48ppm) as identified using STOCY. Spectra with high threshold of cross correlation (CC) value >0.85 (in green, top panel); intermediate confidence with CC between 0.75 to 0.85 (in amber, middle panel) and those considered with no features with cc < 0.75 (in red, bottom panel).

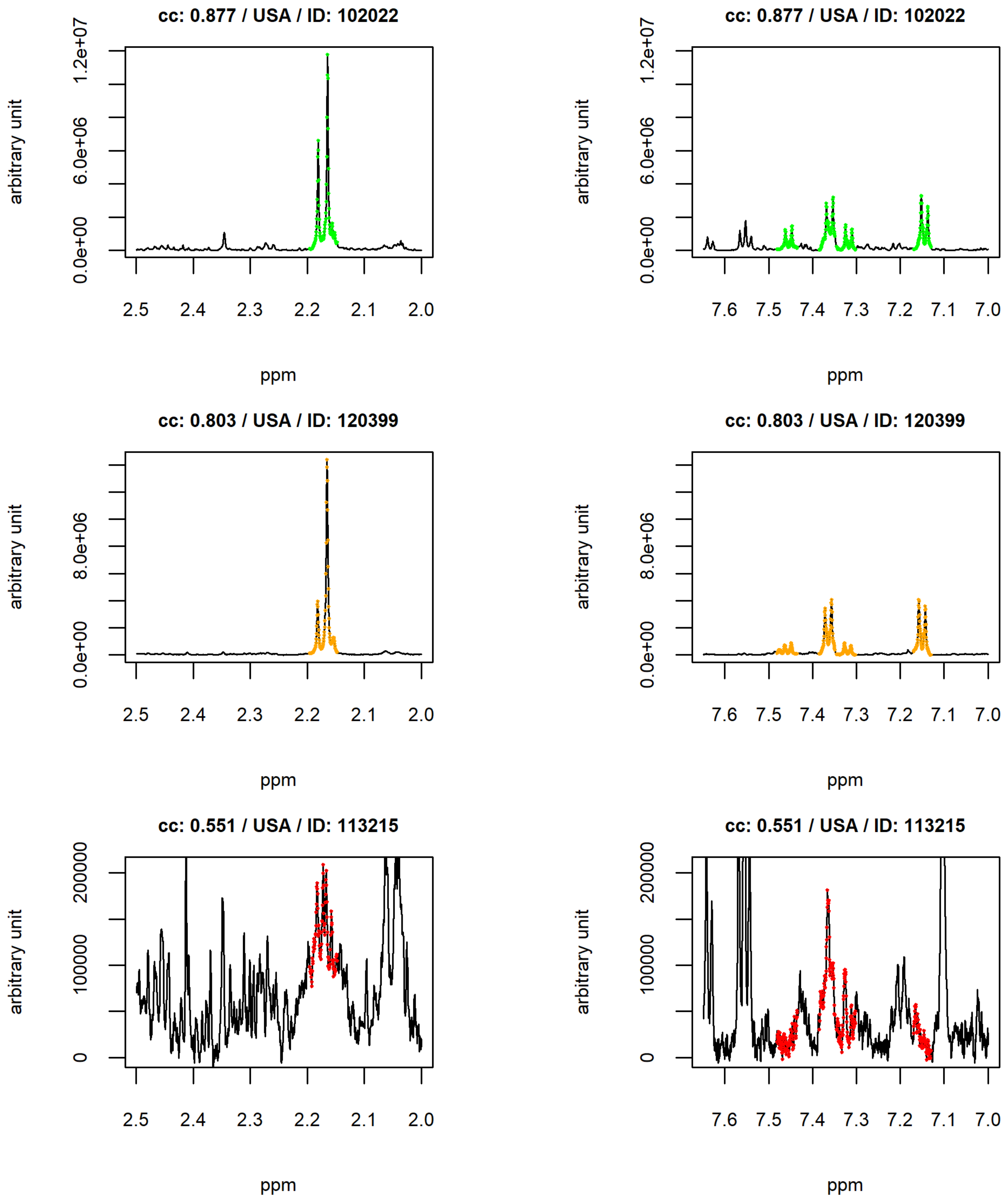

Supplementary Figure 8D: A ROC curve comparing between COMPASS and previous published work by Loo *et al* 2012.

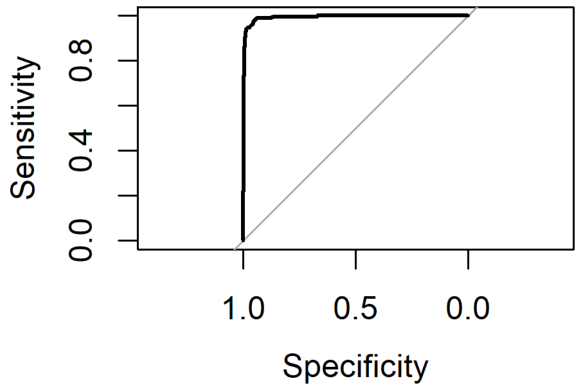

The AURC is: 0.9945232.

Supplementary Figure 8E: Visual inspection of 56 spectra that are deemed to contain acetaminophen metabolites based on CC threshold of 0.75 but was not predicted as containing acetaminophen metabolites in previous publication by Loo *et al*. 2012. Visual inspection of these spectra show acetaminophen metabolites can be observed in these spectra. Spectra are displayed using a traffic light system (green for high CC > 0.85 and amber for intermediate CC at between 07 – 0.85).

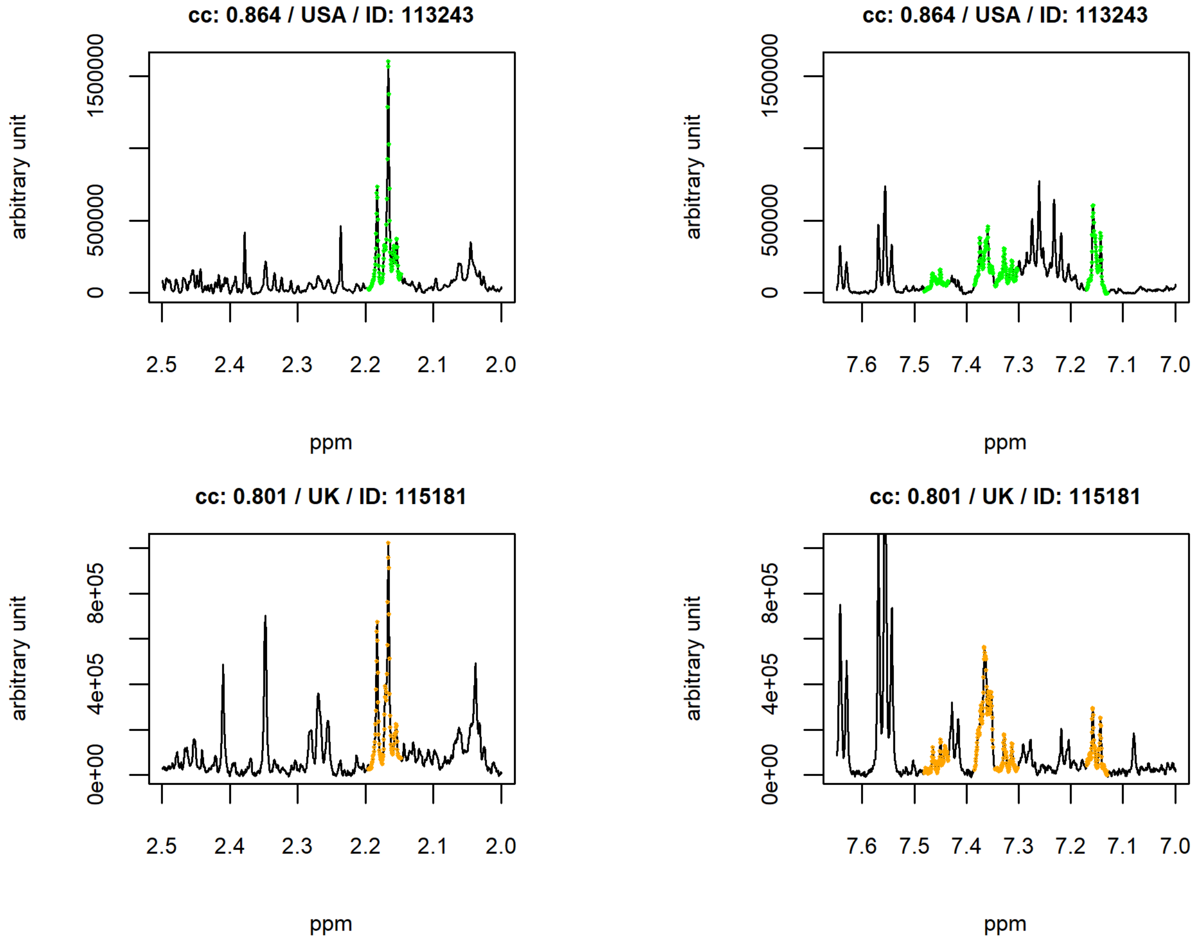

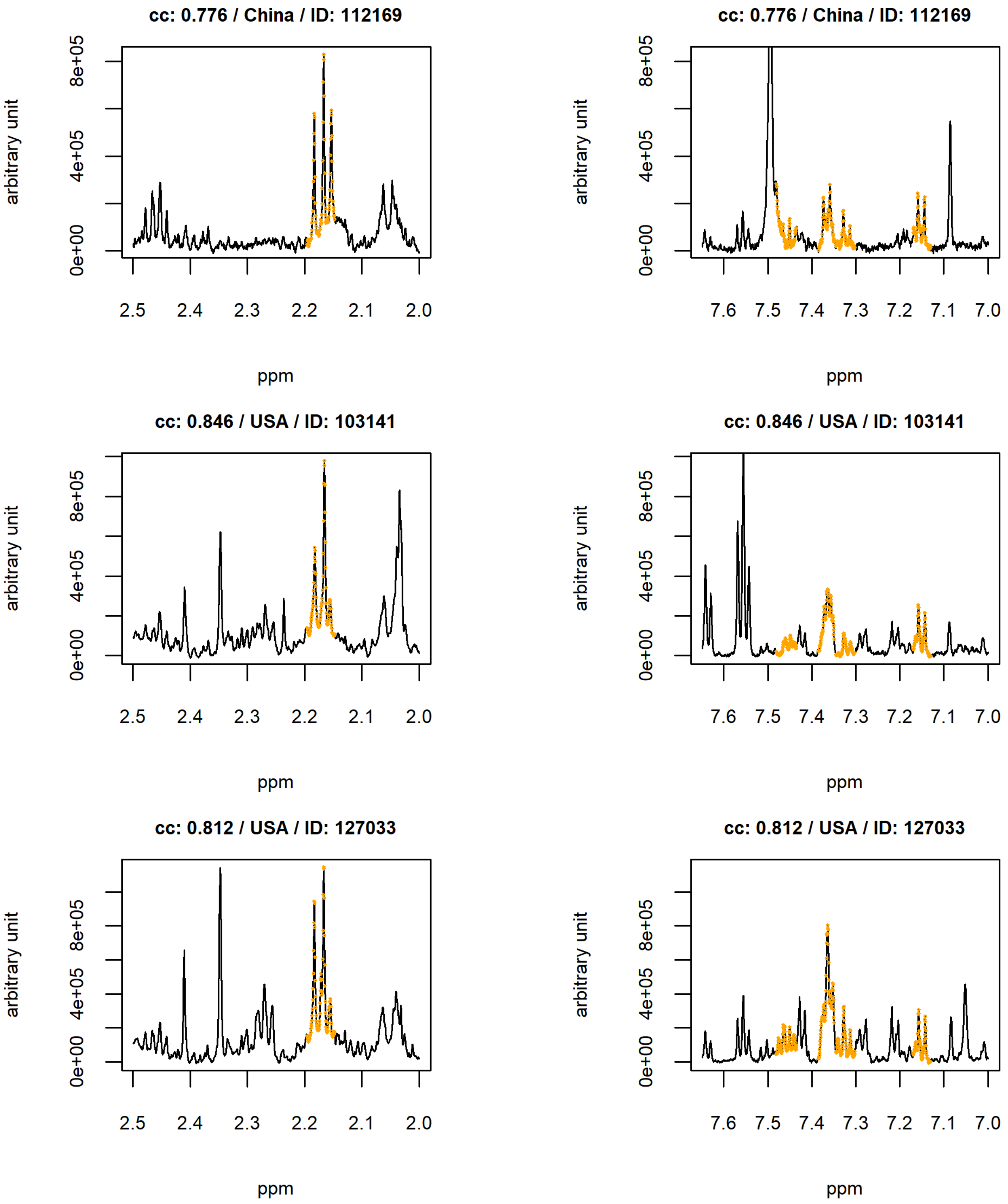
cc: 0.776 / China / ID: 112169
arbitrary unit
0e+00
4e+05
8e+05
2.5
2.4
2.3
2.2
2.1
2.0
ppm
cc: 0.776 / China / ID: 112169
arbitrary unit
0e+00
4e+05
8e+05
7.6
7.5
7.4
7.3
7.2
7.1
7.0
ppm
cc: 0.846 / USA / ID: 103141
arbitrary unit
0e+00
4e+05
8e+05
2.5
2.4
2.3
2.2
2.1
2.0
ppm
cc: 0.846 / USA / ID: 103141
arbitrary unit
0e+00
4e+05
8e+05
7.6
7.5
7.4
7.3
7.2
7.1
7.0
ppm
cc: 0.812 / USA / ID: 127033
arbitrary unit
0e+00
4e+05
8e+05
2.5
2.4
2.3
2.2
2.1
2.0
ppm
cc: 0.812 / USA / ID: 127033
arbitrary unit
0e+00
4e+05
8e+05
7.6
7.5
7.4
7.3
7.2
7.1
7.0
ppm

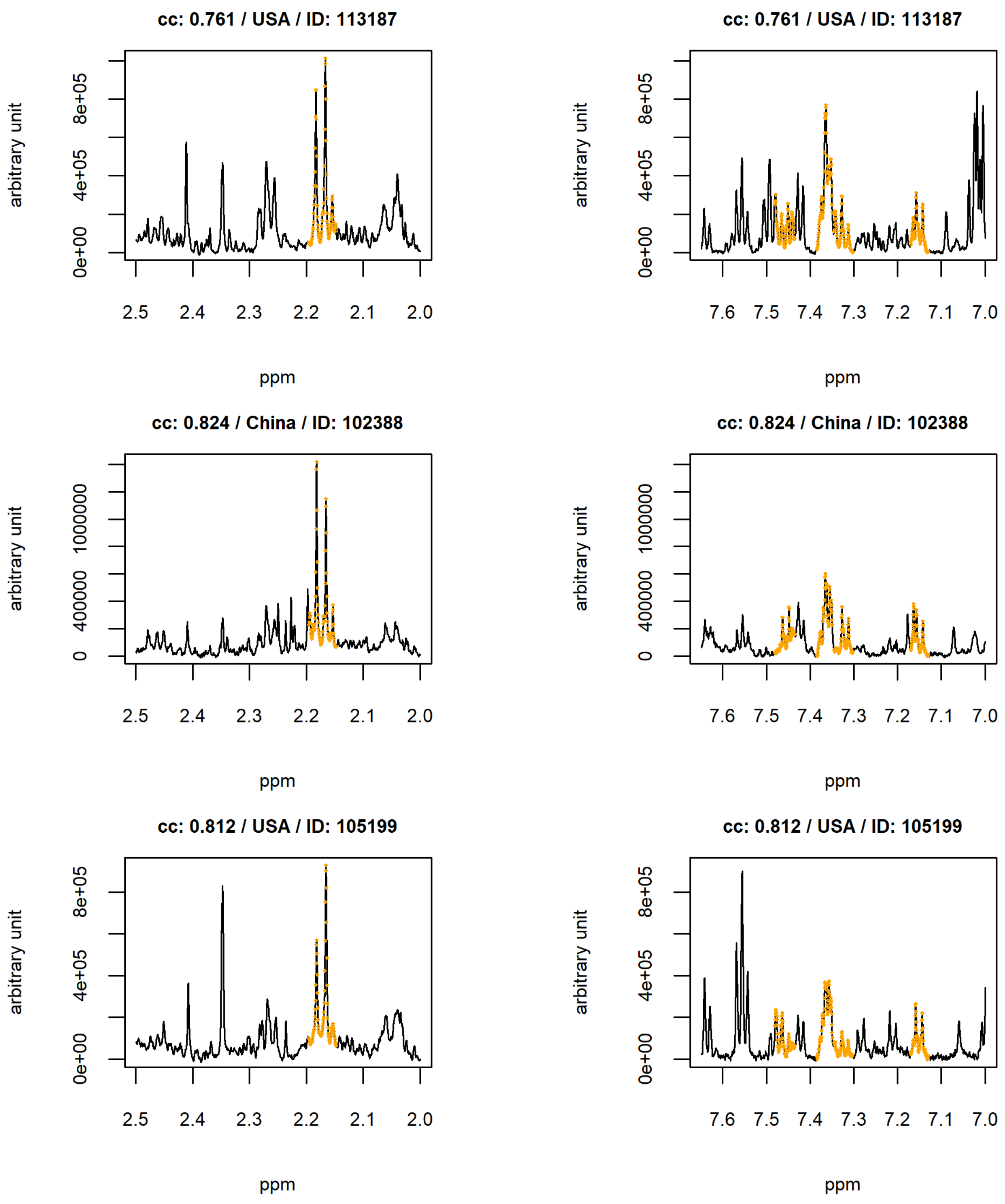
cc: 0.761 / USA / ID: 113187
arbitrary unit
0e+00
4e+05
8e+05
2.5
2.4
2.3
2.2
2.1
2.0
ppm
cc: 0.761 / USA / ID: 113187
arbitrary unit
0e+00
4e+05
8e+05
7.6
7.5
7.4
7.3
7.2
7.1
7.0
ppm
cc: 0.824 / China / ID: 102388
arbitrary unit
0
400000
1000000
2.5
2.4
2.3
2.2
2.1
2.0
ppm
cc: 0.824 / China / ID: 102388
arbitrary unit
0
400000
1000000
7.6
7.5
7.4
7.3
7.2
7.1
7.0
ppm
cc: 0.812 / USA / ID: 105199
arbitrary unit
0e+00
4e+05
8e+05
2.5
2.4
2.3
2.2
2.1
2.0
ppm
cc: 0.812 / USA / ID: 105199
arbitrary unit
0e+00
4e+05
8e+05
7.6
7.5
7.4
7.3
7.2
7.1
7.0
ppm

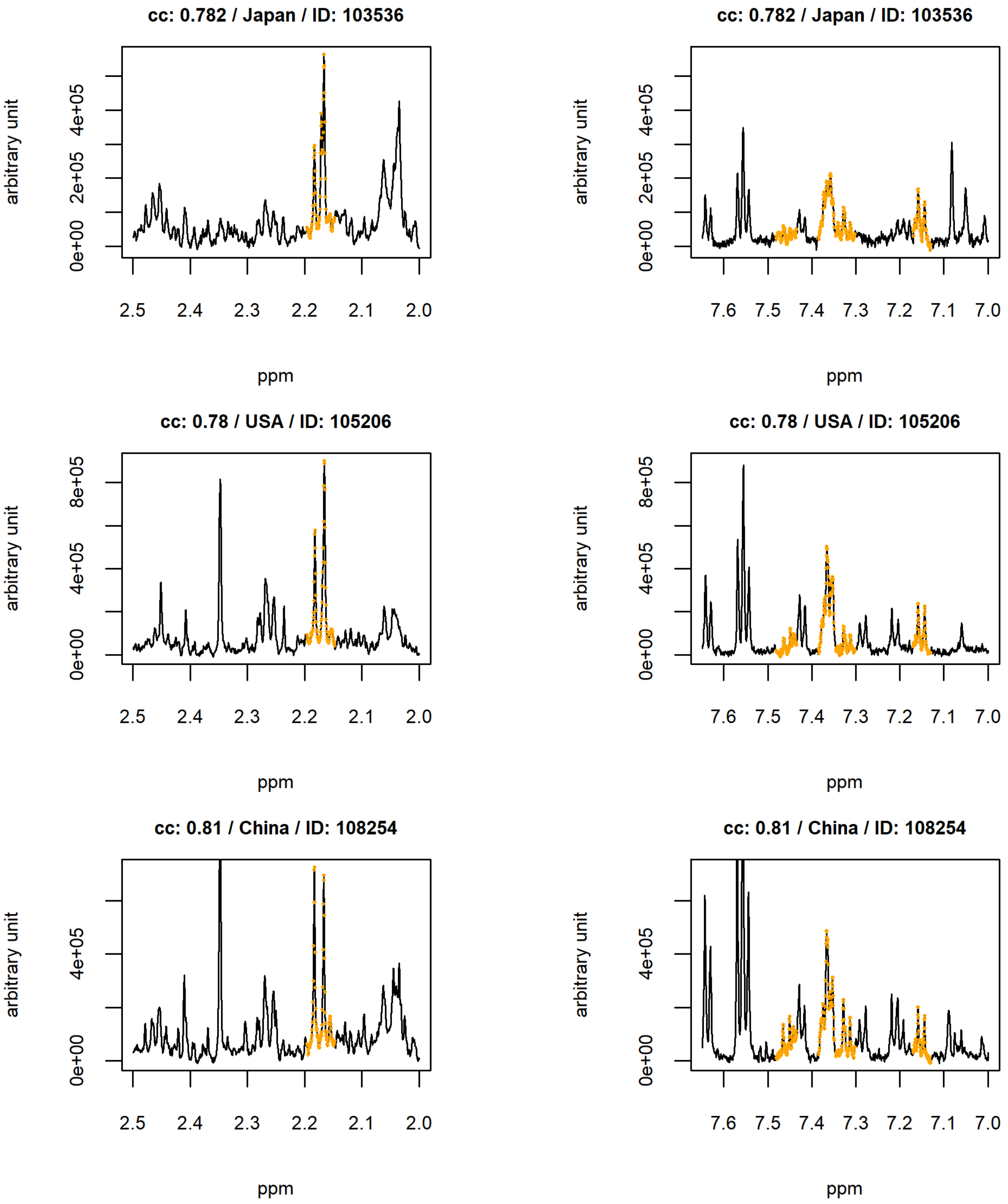

cc: 0.782 / Japan / ID: 103536
arbitrary unit
0e+00 2e+05 4e+05
2.5 2.4 2.3 2.2 2.1 2.0
ppm
cc: 0.782 / Japan / ID: 103536
arbitrary unit
0e+00 2e+05 4e+05
7.6 7.5 7.4 7.3 7.2 7.1 7.0
ppm
cc: 0.78 / USA / ID: 105206
arbitrary unit
0e+00 4e+05 8e+05
2.5 2.4 2.3 2.2 2.1 2.0
ppm
cc: 0.78 / USA / ID: 105206
arbitrary unit
0e+00 4e+05 8e+05
7.6 7.5 7.4 7.3 7.2 7.1 7.0
ppm
cc: 0.81 / China / ID: 108254
arbitrary unit
0e+00 4e+05
2.5 2.4 2.3 2.2 2.1 2.0
ppm
cc: 0.81 / China / ID: 108254
arbitrary unit
0e+00 4e+05
7.6 7.5 7.4 7.3 7.2 7.1 7.0
ppm

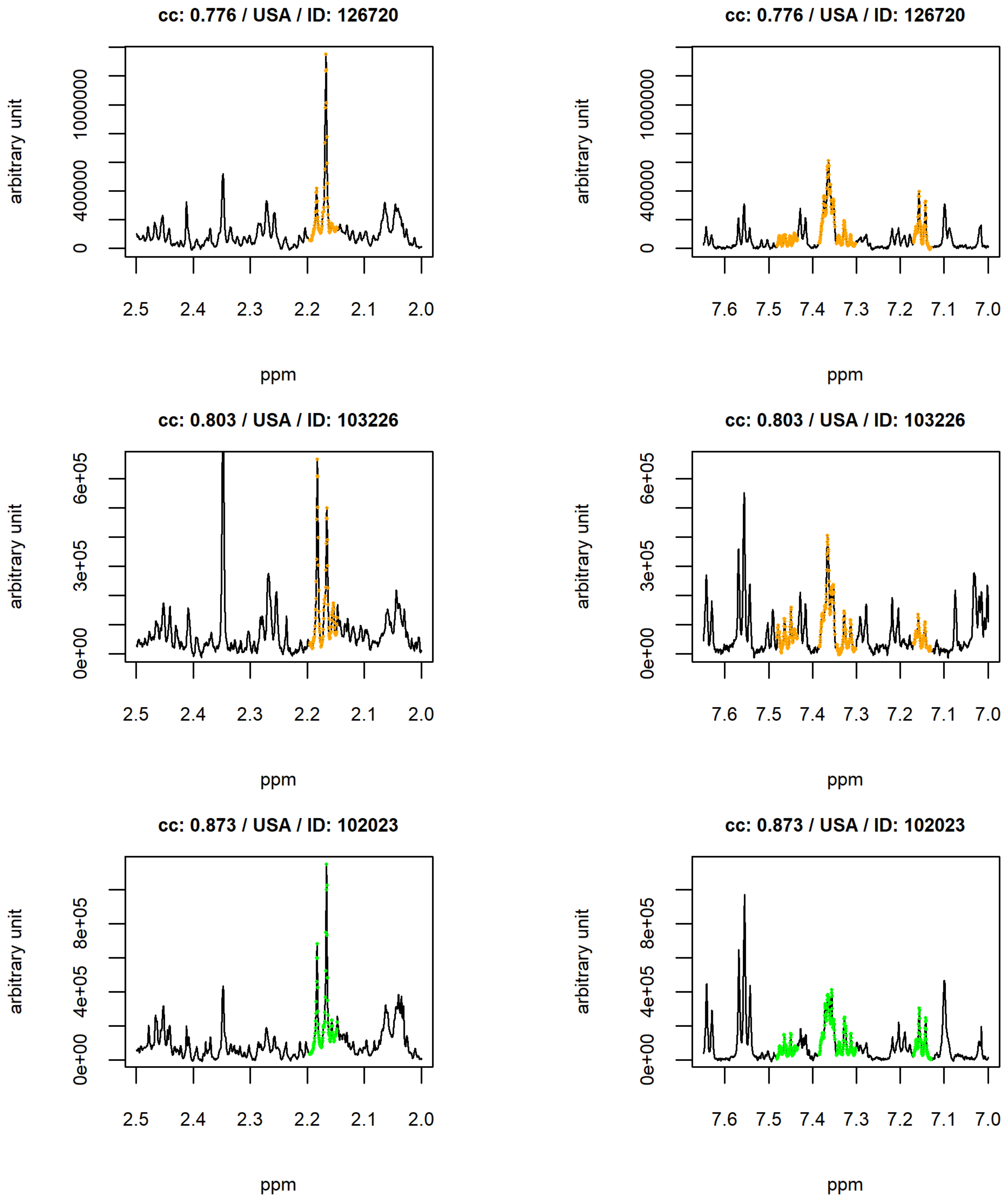

cc: 0.776 / USA / ID: 126720
arbitrary unit
0
400000
1000000
2.5
2.4
2.3
2.2
2.1
2.0
ppm
cc: 0.776 / USA / ID: 126720
arbitrary unit
0
400000
1000000
7.6
7.5
7.4
7.3
7.2
7.1
7.0
ppm
cc: 0.803 / USA / ID: 103226
arbitrary unit
0e+00
3e+05
6e+05
2.5
2.4
2.3
2.2
2.1
2.0
ppm
cc: 0.803 / USA / ID: 103226
arbitrary unit
0e+00
3e+05
6e+05
7.6
7.5
7.4
7.3
7.2
7.1
7.0
ppm
cc: 0.873 / USA / ID: 102023
arbitrary unit
0e+00
4e+05
8e+05
2.5
2.4
2.3
2.2
2.1
2.0
ppm
cc: 0.873 / USA / ID: 102023
arbitrary unit
0e+00
4e+05
8e+05
7.6
7.5
7.4
7.3
7.2
7.1
7.0
ppm

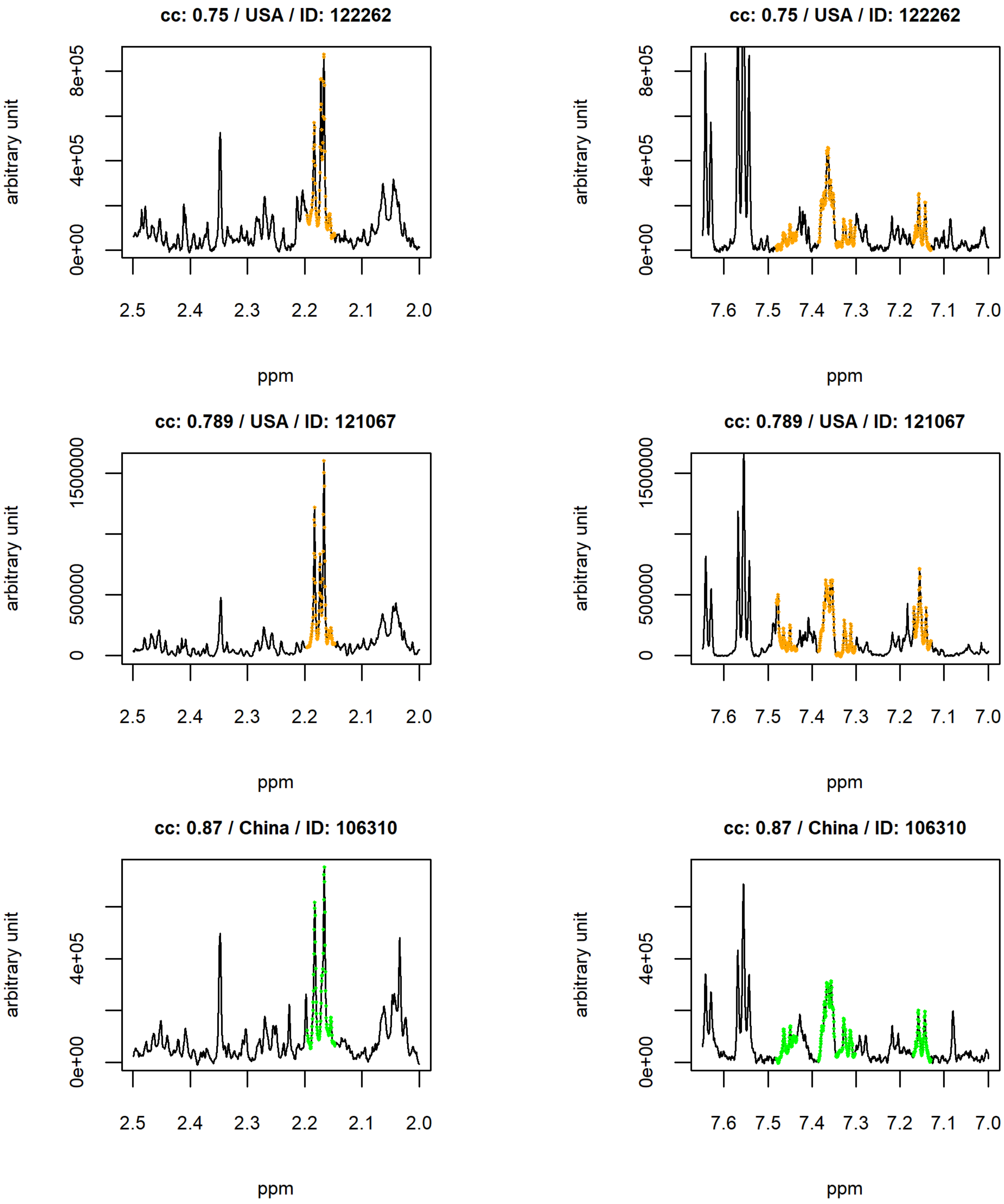
cc: 0.75 / USA / ID: 122262
cc: 0.75 / USA / ID: 122262
cc: 0.789 / USA / ID: 121067
cc: 0.789 / USA / ID: 121067
cc: 0.87 / China / ID: 106310
cc: 0.87 / China / ID: 106310
arbitrary unit
ppm
0e+00
4e+05
8e+05
0
500000
1500000
2.5
2.4
2.3
2.2
2.1
2.0
7.6
7.5
7.4
7.3
7.2
7.1
7.0

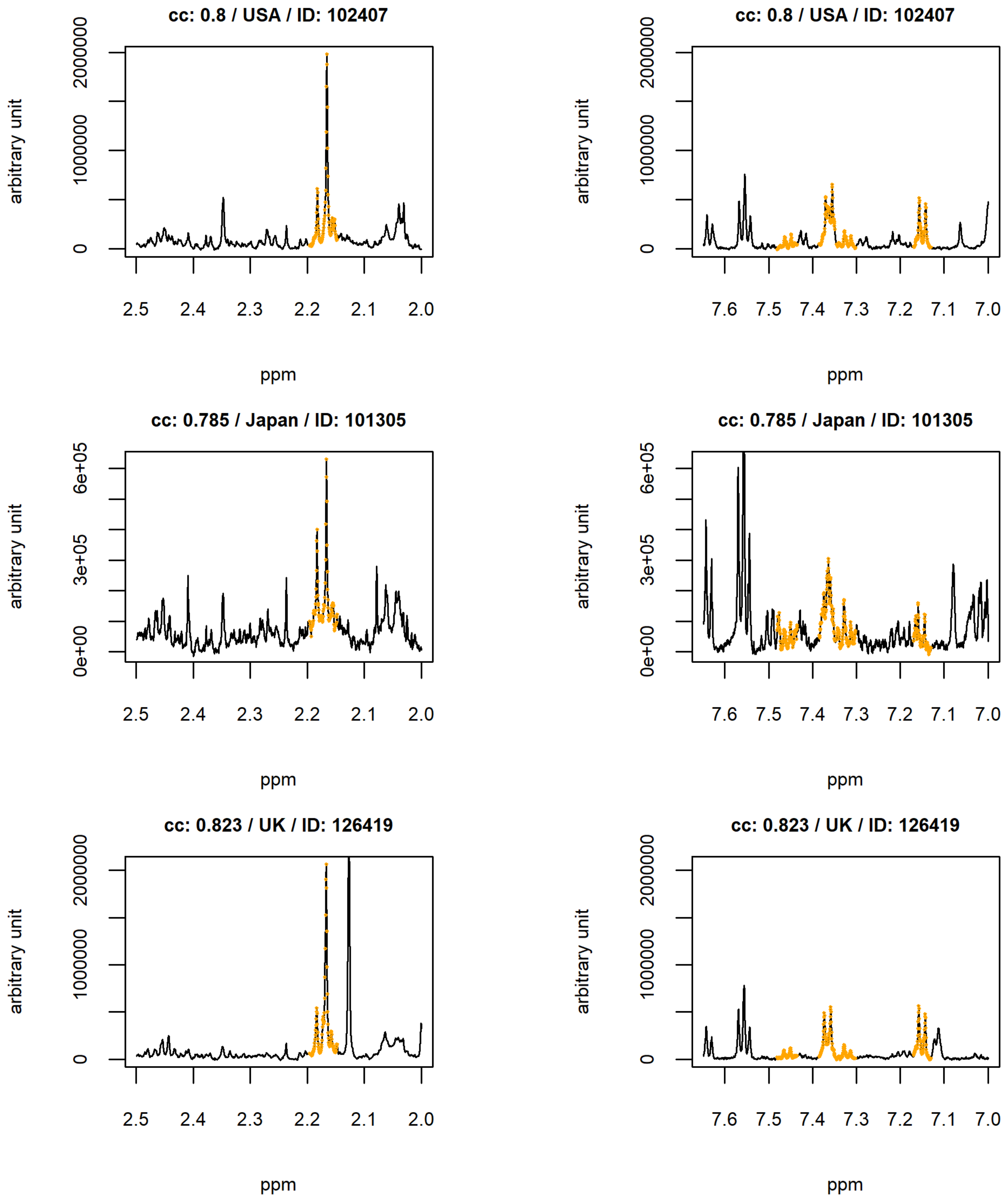
cc: 0.8 / USA / ID: 102407
arbitrary unit
0
1000000
2000000
2.5
2.4
2.3
2.2
2.1
2.0
ppm
cc: 0.8 / USA / ID: 102407
arbitrary unit
0
1000000
2000000
7.6
7.5
7.4
7.3
7.2
7.1
7.0
ppm
cc: 0.785 / Japan / ID: 101305
arbitrary unit
0e+00
3e+05
6e+05
2.5
2.4
2.3
2.2
2.1
2.0
ppm
cc: 0.785 / Japan / ID: 101305
arbitrary unit
0e+00
3e+05
6e+05
7.6
7.5
7.4
7.3
7.2
7.1
7.0
ppm
cc: 0.823 / UK / ID: 126419
arbitrary unit
0
1000000
2000000
2.5
2.4
2.3
2.2
2.1
2.0
ppm
cc: 0.823 / UK / ID: 126419
arbitrary unit
0
1000000
2000000
7.6
7.5
7.4
7.3
7.2
7.1
7.0
ppm

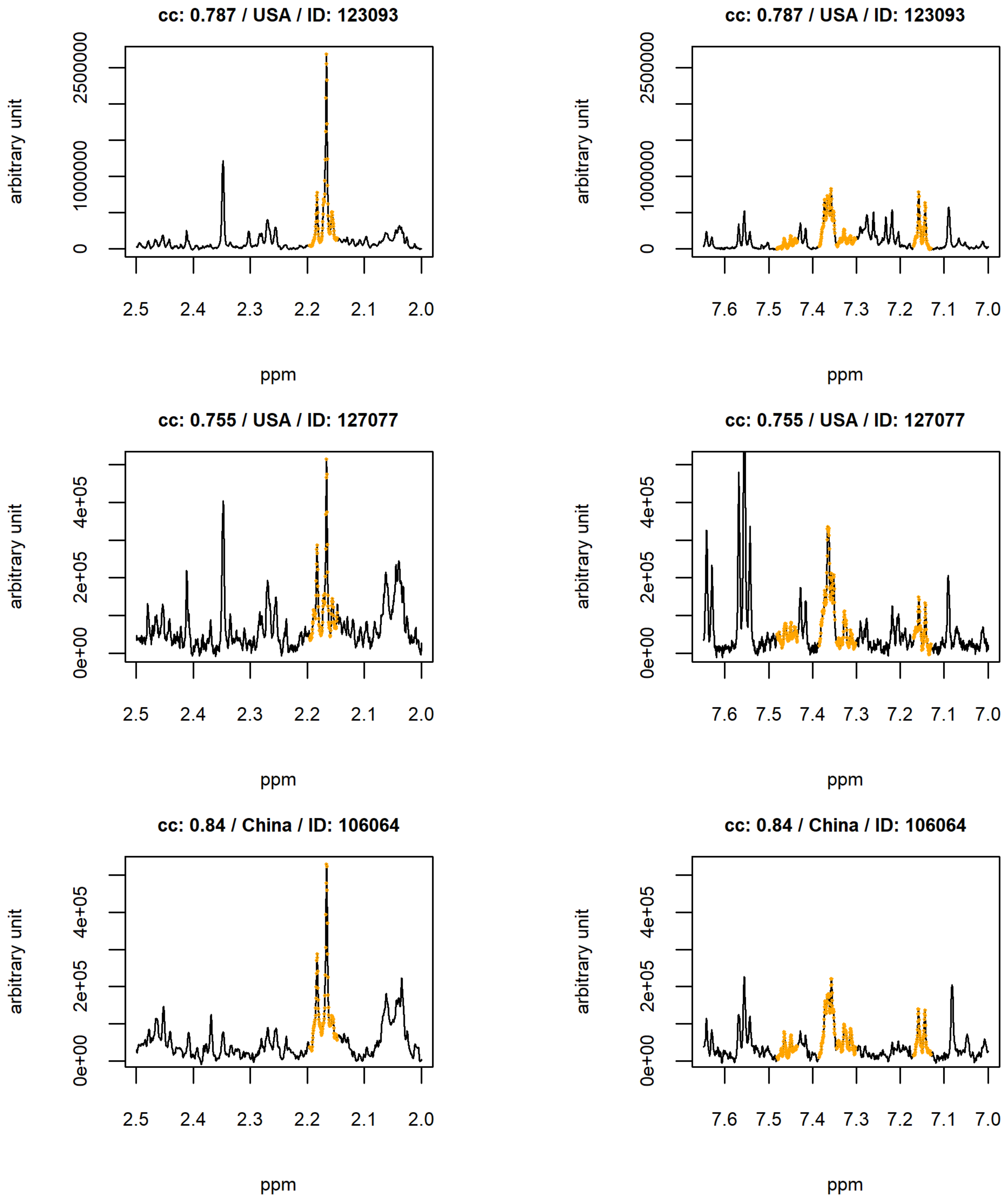

cc: 0.787 / USA / ID: 123093
cc: 0.787 / USA / ID: 123093
cc: 0.755 / USA / ID: 127077
cc: 0.755 / USA / ID: 127077
cc: 0.84 / China / ID: 106064
cc: 0.84 / China / ID: 106064
arbitrary unit
ppm
0
1000000
2500000
0e+00
2e+05
4e+05
2.5
2.4
2.3
2.2
2.1
2.0
7.6
7.5
7.4
7.3
7.2
7.1
7.0

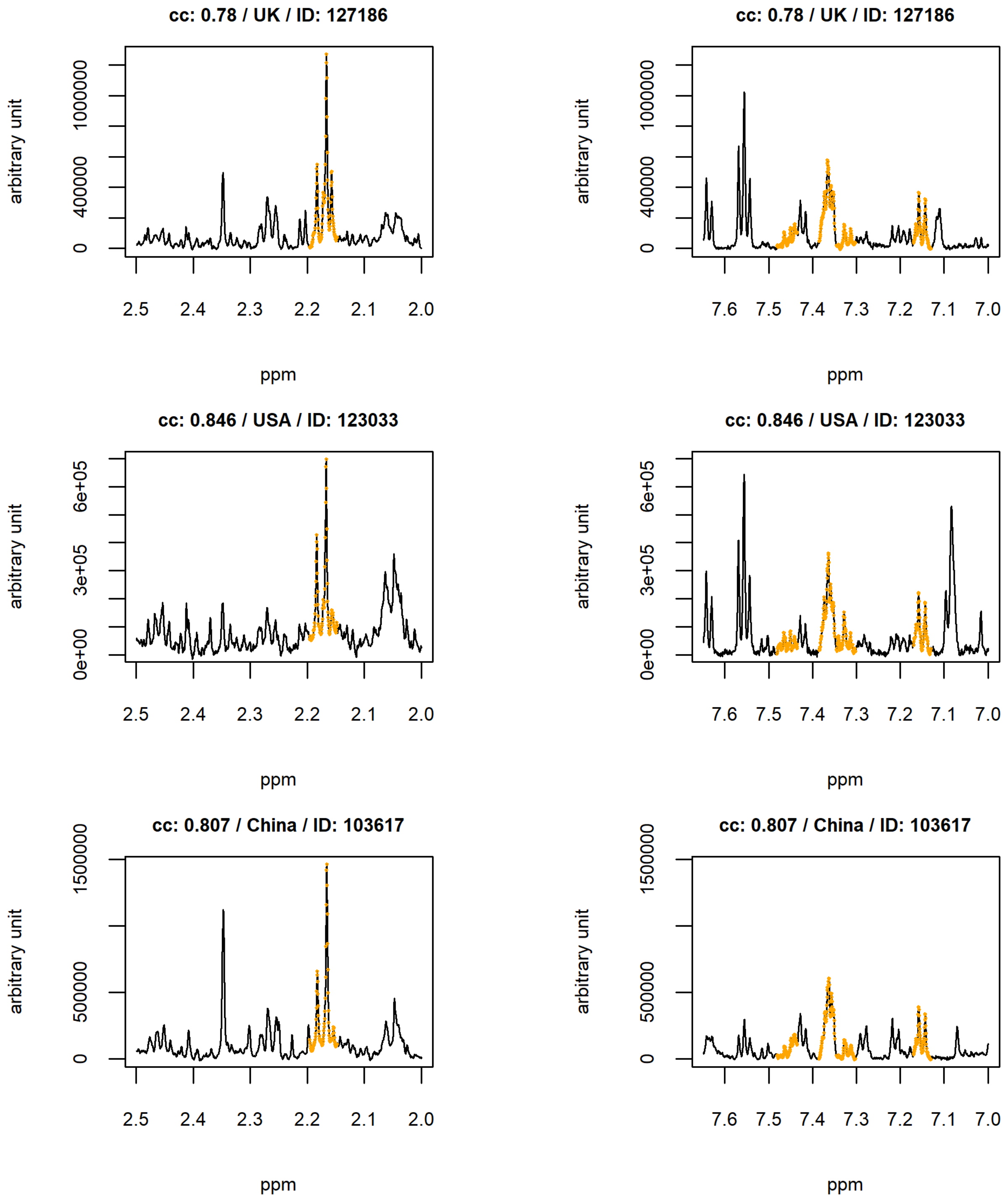
cc: 0.78 / UK / ID: 127186
arbitrary unit
0
400000
1000000
2.5
2.4
2.3
2.2
2.1
2.0
ppm
cc: 0.78 / UK / ID: 127186
arbitrary unit
0
400000
1000000
7.6
7.5
7.4
7.3
7.2
7.1
7.0
ppm
cc: 0.846 / USA / ID: 123033
arbitrary unit
0e+00
3e+05
6e+05
2.5
2.4
2.3
2.2
2.1
2.0
ppm
cc: 0.846 / USA / ID: 123033
arbitrary unit
0e+00
3e+05
6e+05
7.6
7.5
7.4
7.3
7.2
7.1
7.0
ppm
cc: 0.807 / China / ID: 103617
arbitrary unit
0
500000
1500000
2.5
2.4
2.3
2.2
2.1
2.0
ppm
cc: 0.807 / China / ID: 103617
arbitrary unit
0
500000
1500000
7.6
7.5
7.4
7.3
7.2
7.1
7.0
ppm

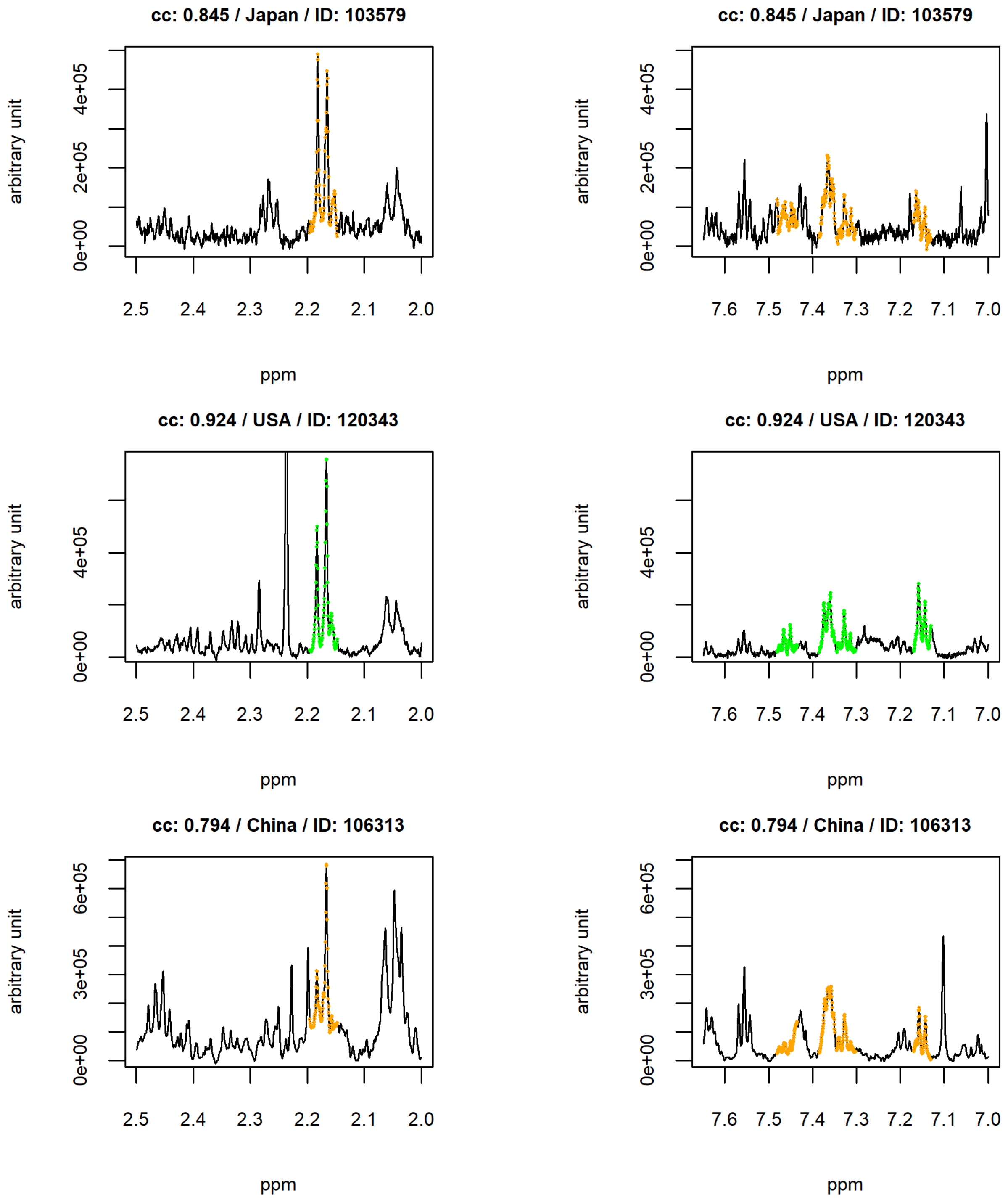
cc: 0.845 / Japan / ID: 103579
cc: 0.845 / Japan / ID: 103579
cc: 0.924 / USA / ID: 120343
cc: 0.924 / USA / ID: 120343
cc: 0.794 / China / ID: 106313
cc: 0.794 / China / ID: 106313
arbitrary unit
ppm
0e+00
2e+05
4e+05
3e+05
6e+05
2.5
2.4
2.3
2.2
2.1
2.0
7.6
7.5
7.4
7.3
7.2
7.1
7.0

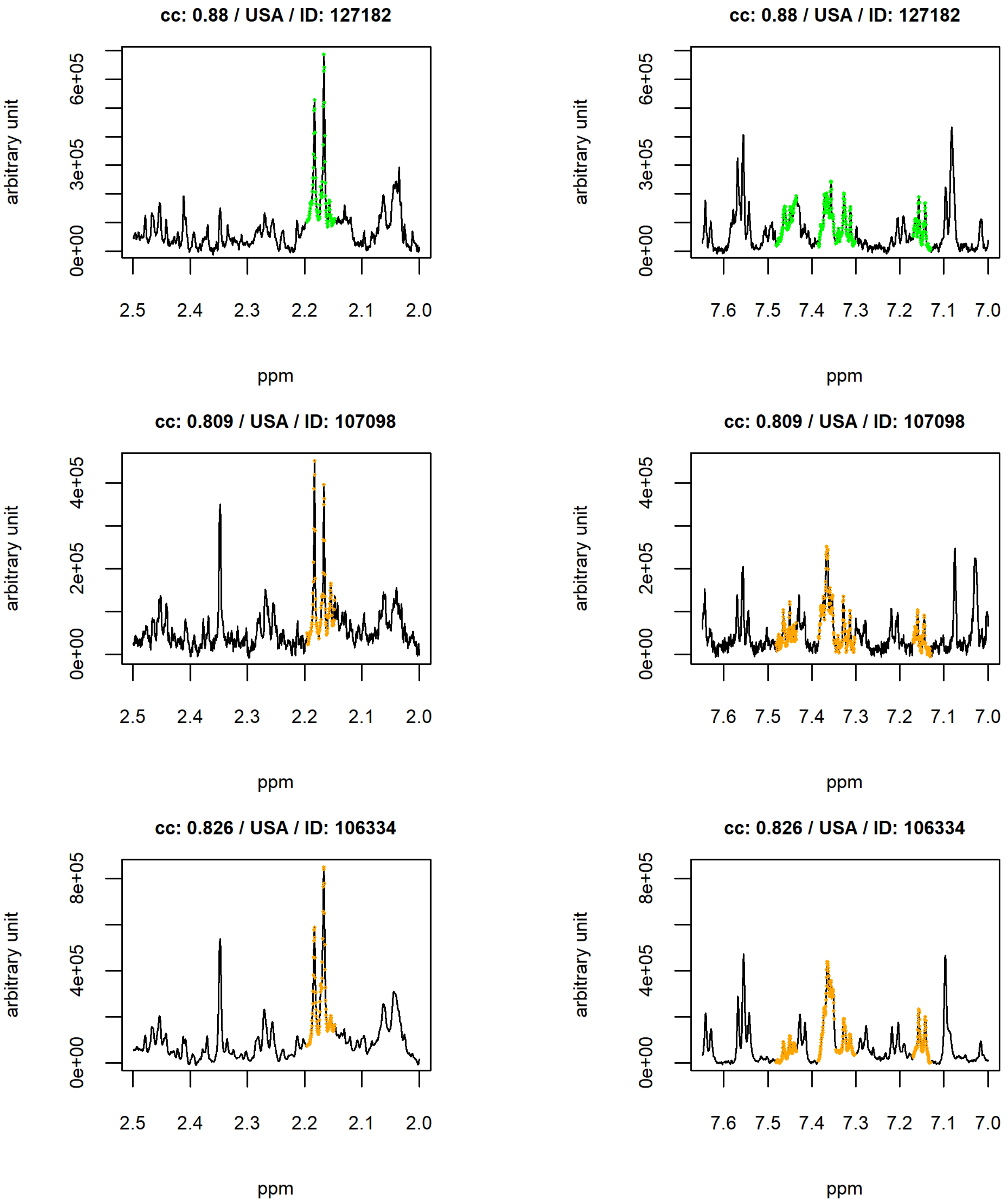
cc: 0.88 / USA / ID: 127182
cc: 0.88 / USA / ID: 127182
cc: 0.809 / USA / ID: 107098
cc: 0.809 / USA / ID: 107098
cc: 0.826 / USA / ID: 106334
cc: 0.826 / USA / ID: 106334
arbitrary unit
ppm
0e+00
3e+05
6e+05
2e+05
4e+05
8e+05
2.5
2.4
2.3
2.2
2.1
2.0
7.6
7.5
7.4
7.3
7.2
7.1
7.0

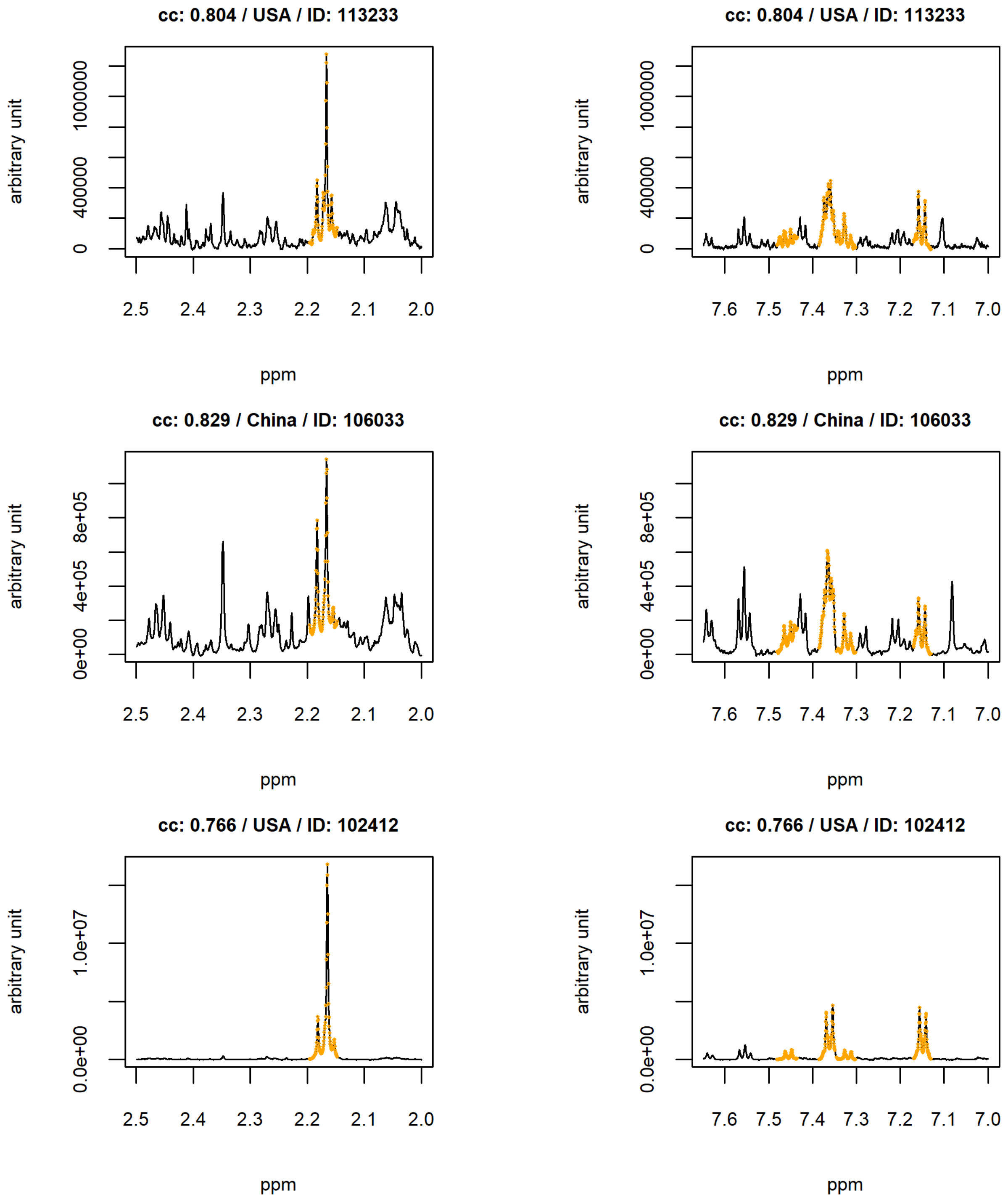
cc: 0.804 / USA / ID: 113233
arbitrary unit
0
400000
1000000
2.5
2.4
2.3
2.2
2.1
2.0
ppm
cc: 0.804 / USA / ID: 113233
arbitrary unit
0
400000
1000000
7.6
7.5
7.4
7.3
7.2
7.1
7.0
ppm
cc: 0.829 / China / ID: 106033
arbitrary unit
0e+00
4e+05
8e+05
2.5
2.4
2.3
2.2
2.1
2.0
ppm
cc: 0.829 / China / ID: 106033
arbitrary unit
0e+00
4e+05
8e+05
7.6
7.5
7.4
7.3
7.2
7.1
7.0
ppm
cc: 0.766 / USA / ID: 102412
arbitrary unit
0.0e+00
1.0e+07
2.5
2.4
2.3
2.2
2.1
2.0
ppm
cc: 0.766 / USA / ID: 102412
arbitrary unit
0.0e+00
1.0e+07
7.6
7.5
7.4
7.3
7.2
7.1
7.0
ppm

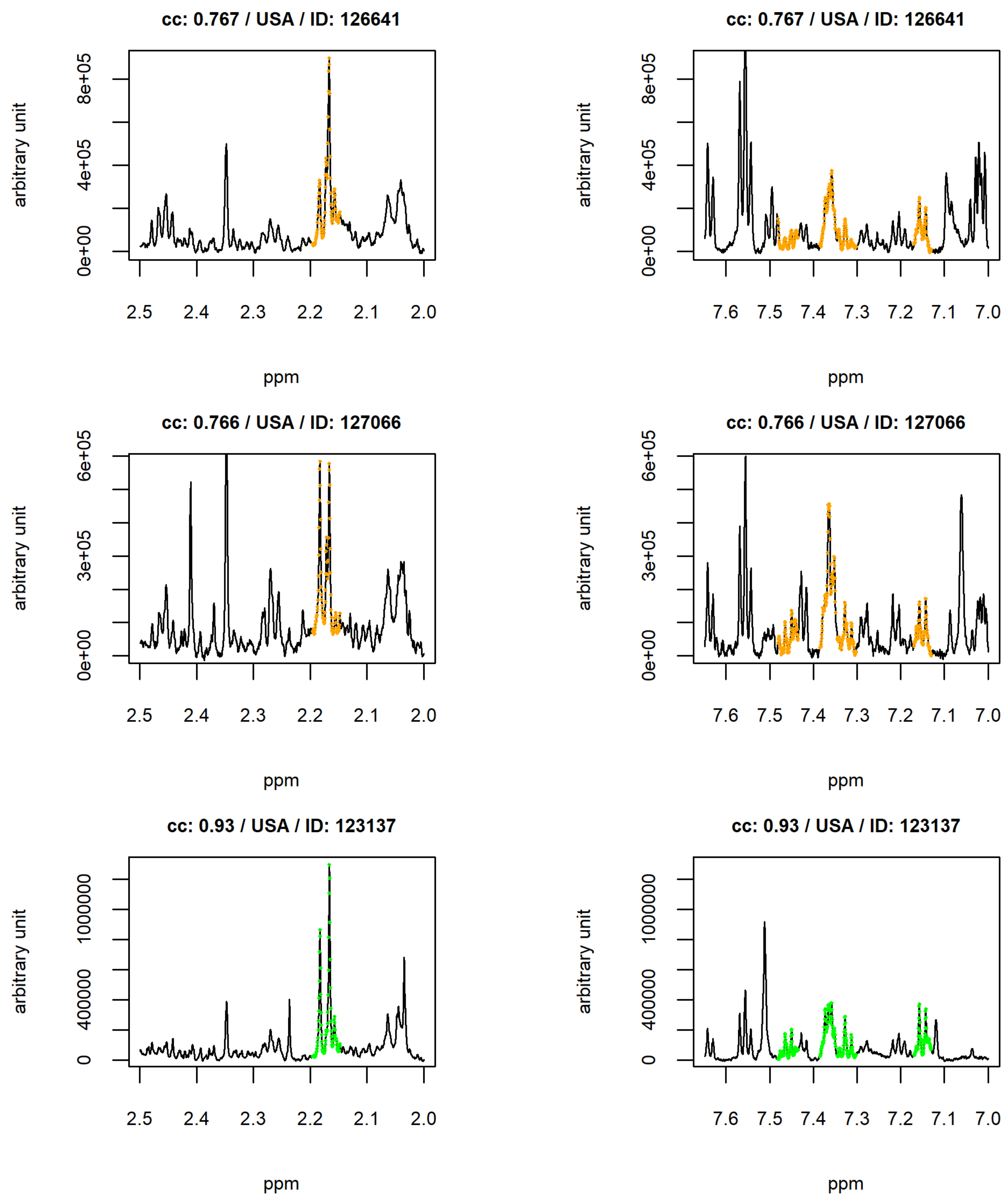
cc: 0.767 / USA / ID: 126641
cc: 0.767 / USA / ID: 126641
cc: 0.766 / USA / ID: 127066
cc: 0.766 / USA / ID: 127066
cc: 0.93 / USA / ID: 123137
cc: 0.93 / USA / ID: 123137
arbitrary unit
ppm
0e+00
4e+05
8e+05
3e+05
6e+05
0
400000
1000000
2.5
2.4
2.3
2.2
2.1
2.0
7.6
7.5
7.4
7.3
7.2
7.1
7.0

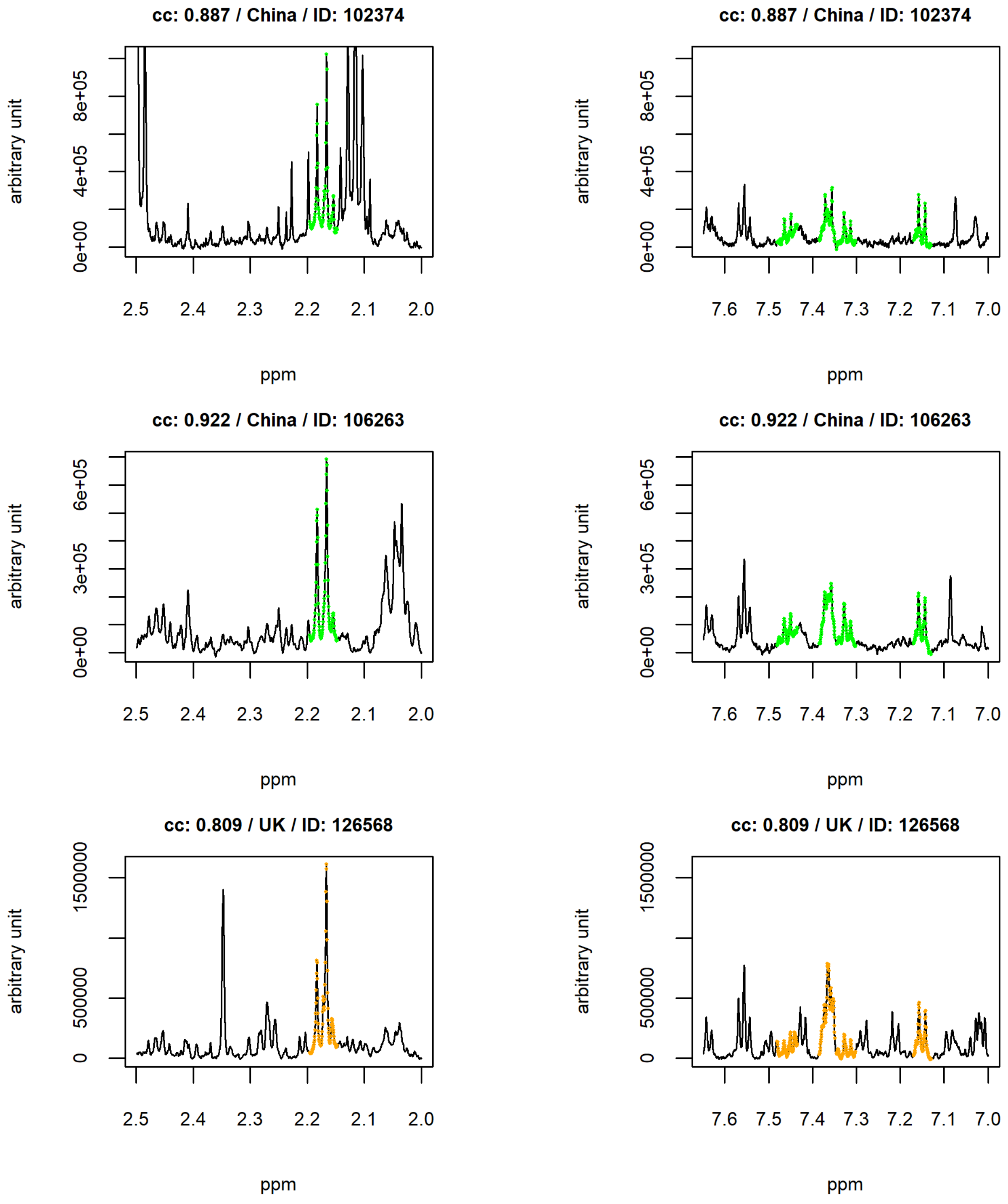
cc: 0.887 / China / ID: 102374
arbitrary unit
0e+00
4e+05
8e+05
2.5
2.4
2.3
2.2
2.1
2.0
ppm
cc: 0.887 / China / ID: 102374
arbitrary unit
0e+00
4e+05
8e+05
7.6
7.5
7.4
7.3
7.2
7.1
7.0
ppm
cc: 0.922 / China / ID: 106263
arbitrary unit
0e+00
3e+05
6e+05
2.5
2.4
2.3
2.2
2.1
2.0
ppm
cc: 0.922 / China / ID: 106263
arbitrary unit
0e+00
3e+05
6e+05
7.6
7.5
7.4
7.3
7.2
7.1
7.0
ppm
cc: 0.809 / UK / ID: 126568
arbitrary unit
0
500000
1500000
2.5
2.4
2.3
2.2
2.1
2.0
ppm
cc: 0.809 / UK / ID: 126568
arbitrary unit
0
500000
1500000
7.6
7.5
7.4
7.3
7.2
7.1
7.0
ppm

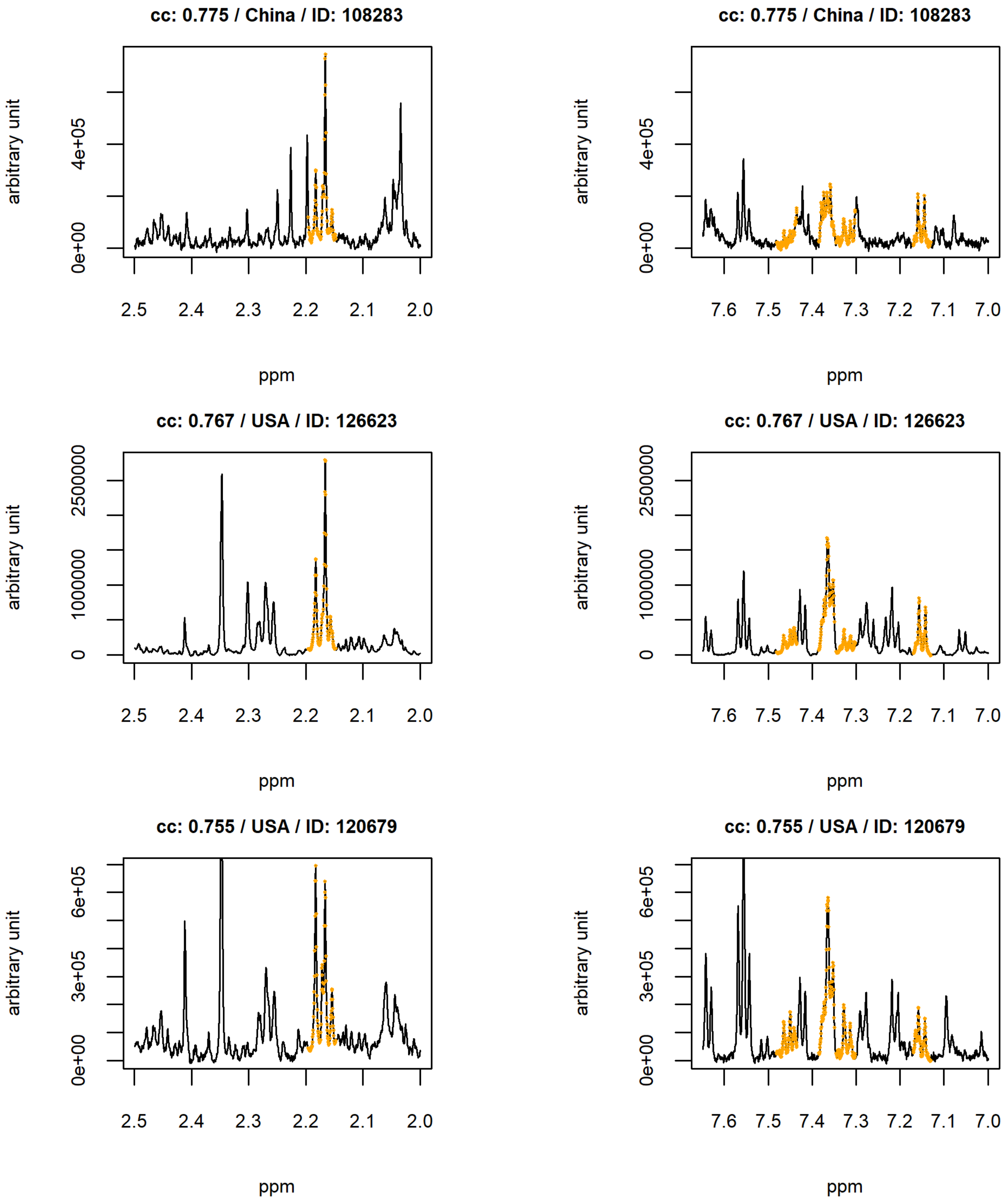
cc: 0.775 / China / ID: 108283
cc: 0.775 / China / ID: 108283
cc: 0.767 / USA / ID: 126623
cc: 0.767 / USA / ID: 126623
cc: 0.755 / USA / ID: 120679
cc: 0.755 / USA / ID: 120679
arbitrary unit
ppm
0e+00
4e+05
0
1000000
2500000
3e+05
6e+05
2.5
2.4
2.3
2.2
2.1
2.0
7.6
7.5
7.4
7.3
7.2
7.1
7.0

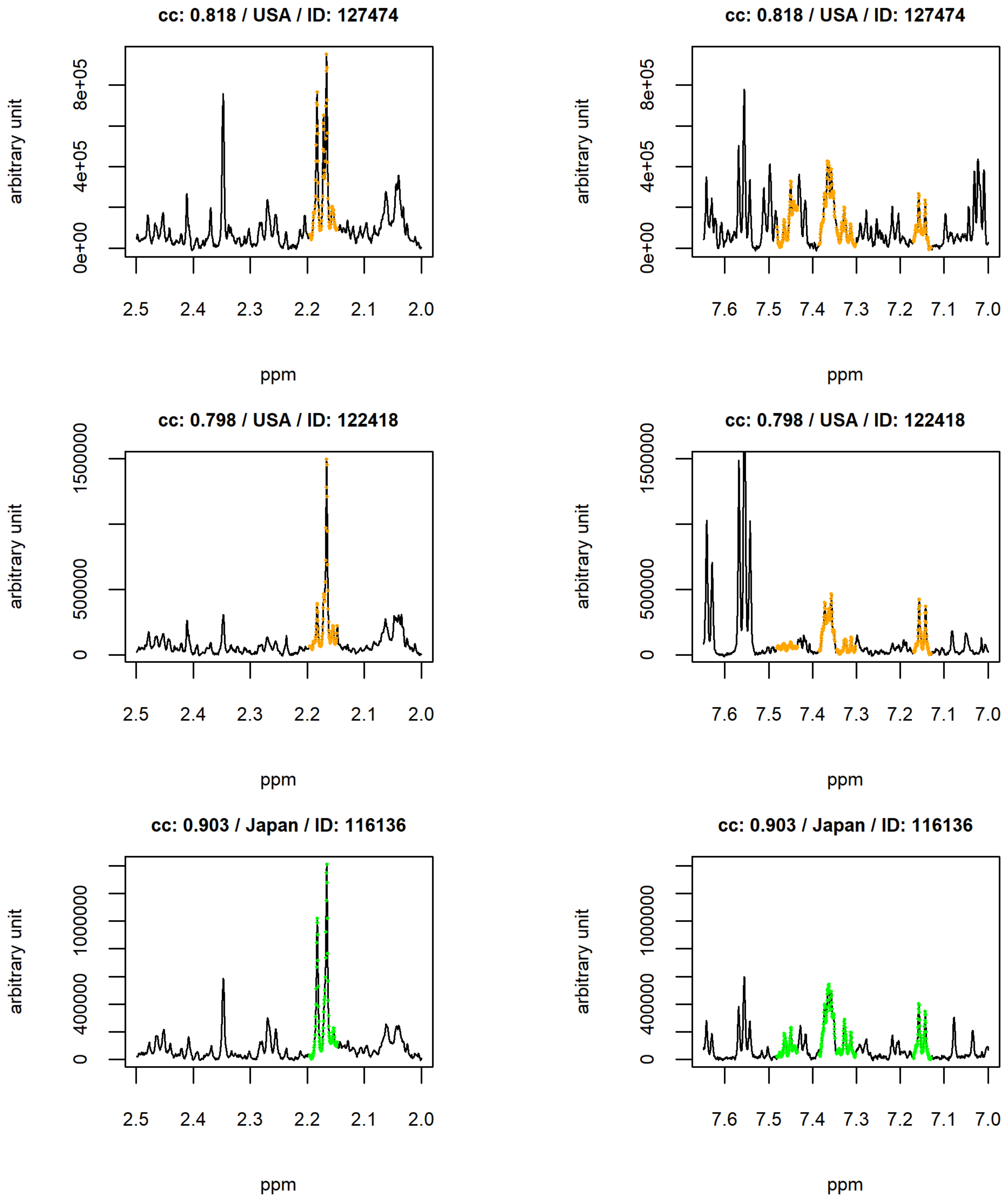
cc: 0.818 / USA / ID: 127474
arbitrary unit
0e+00
4e+05
8e+05
2.5
2.4
2.3
2.2
2.1
2.0
ppm
cc: 0.818 / USA / ID: 127474
arbitrary unit
0e+00
4e+05
8e+05
7.6
7.5
7.4
7.3
7.2
7.1
7.0
ppm
cc: 0.798 / USA / ID: 122418
arbitrary unit
0
500000
1500000
2.5
2.4
2.3
2.2
2.1
2.0
ppm
cc: 0.798 / USA / ID: 122418
arbitrary unit
0
500000
1500000
7.6
7.5
7.4
7.3
7.2
7.1
7.0
ppm
cc: 0.903 / Japan / ID: 116136
arbitrary unit
0
400000
1000000
2.5
2.4
2.3
2.2
2.1
2.0
ppm
cc: 0.903 / Japan / ID: 116136
arbitrary unit
0
400000
1000000
7.6
7.5
7.4
7.3
7.2
7.1
7.0
ppm

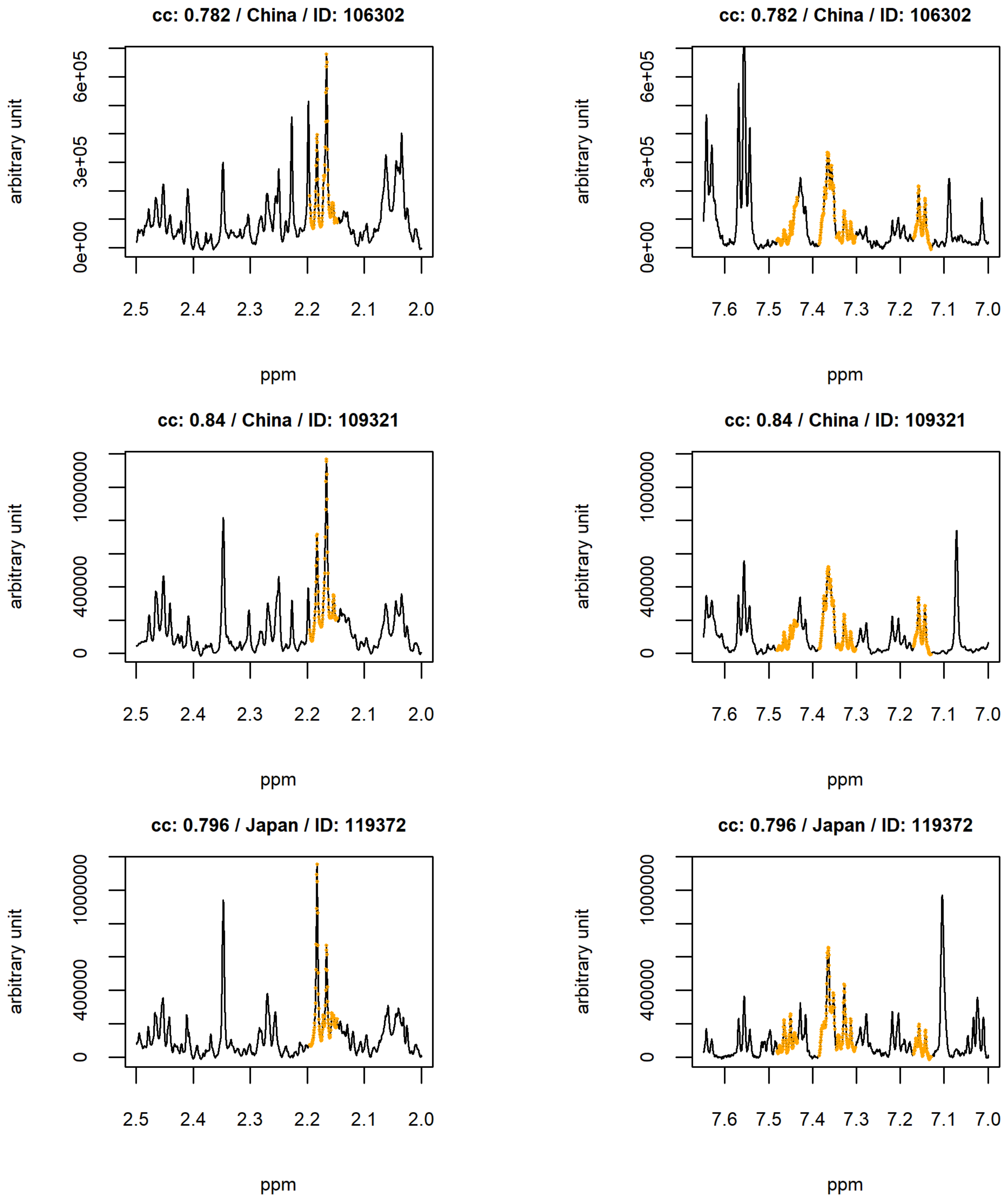
cc: 0.782 / China / ID: 106302
cc: 0.782 / China / ID: 106302
cc: 0.84 / China / ID: 109321
cc: 0.84 / China / ID: 109321
cc: 0.796 / Japan / ID: 119372
cc: 0.796 / Japan / ID: 119372
arbitrary unit
ppm
0e+00
3e+05
6e+05
0
400000
1000000
2.5
2.4
2.3
2.2
2.1
2.0
7.6
7.5
7.4
7.3
7.2
7.1
7.0

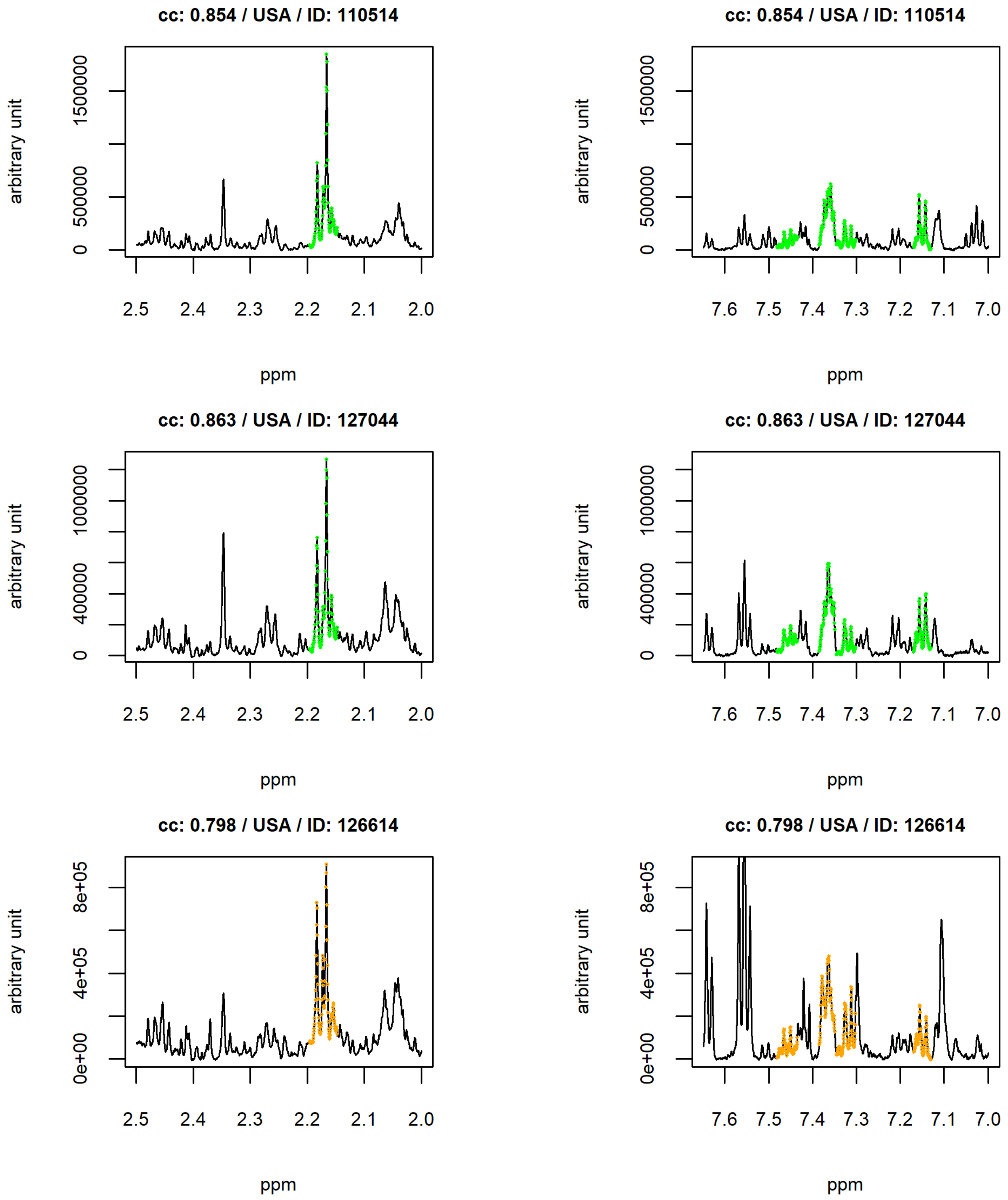
cc: 0.854 / USA / ID: 110514
arbitrary unit
0
500000
1500000
2.5
2.4
2.3
2.2
2.1
2.0
ppm
cc: 0.854 / USA / ID: 110514
arbitrary unit
0
500000
1500000
7.6
7.5
7.4
7.3
7.2
7.1
7.0
ppm
cc: 0.863 / USA / ID: 127044
arbitrary unit
0
400000
1000000
2.5
2.4
2.3
2.2
2.1
2.0
ppm
cc: 0.863 / USA / ID: 127044
arbitrary unit
0
400000
1000000
7.6
7.5
7.4
7.3
7.2
7.1
7.0
ppm
cc: 0.798 / USA / ID: 126614
arbitrary unit
0e+00
4e+05
8e+05
2.5
2.4
2.3
2.2
2.1
2.0
ppm
cc: 0.798 / USA / ID: 126614
arbitrary unit
0e+00
4e+05
8e+05
7.6
7.5
7.4
7.3
7.2
7.1
7.0
ppm

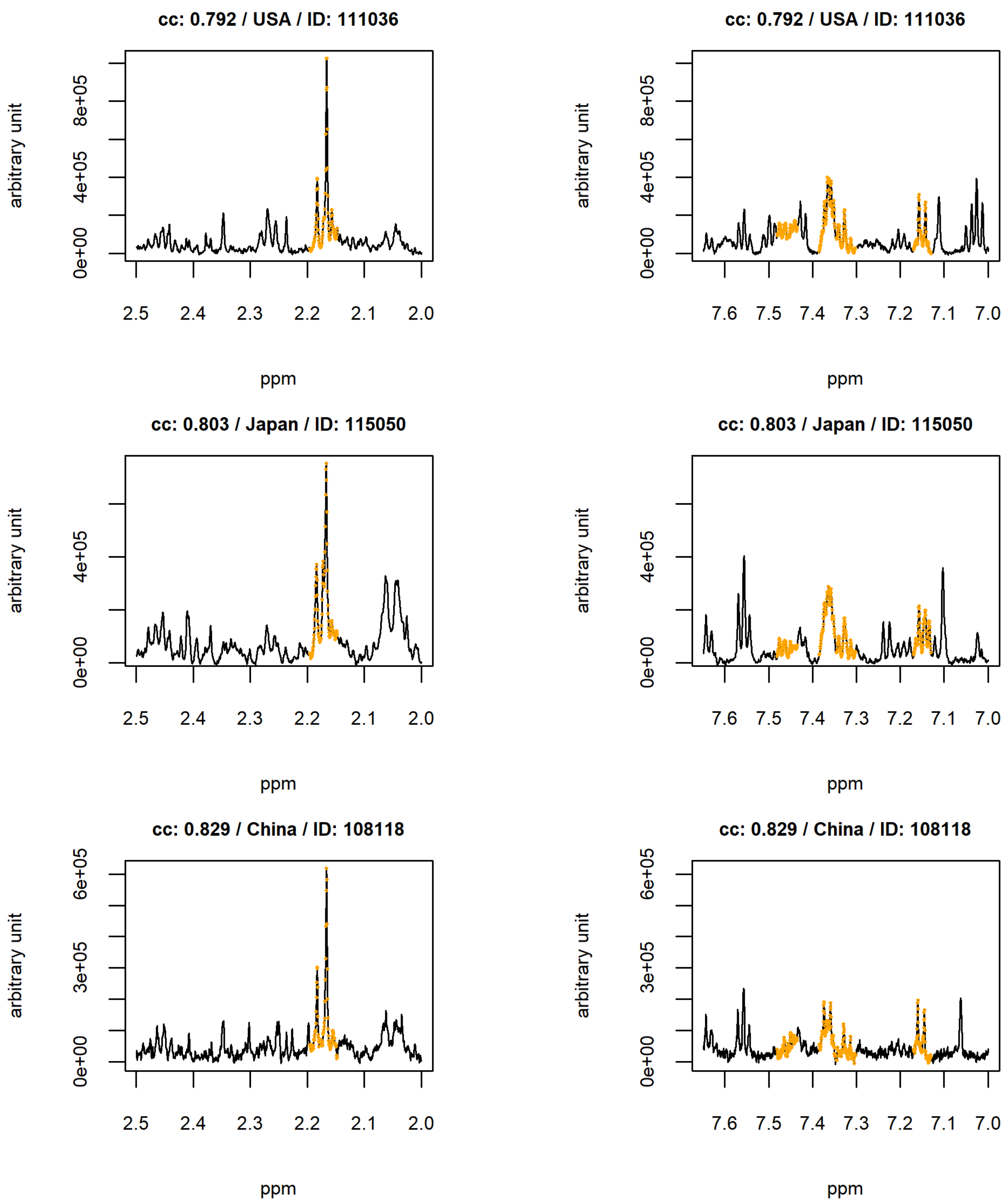
cc: 0.792 / USA / ID: 111036
arbitrary unit
0e+00
4e+05
8e+05
2.5
2.4
2.3
2.2
2.1
2.0
ppm
cc: 0.792 / USA / ID: 111036
arbitrary unit
0e+00
4e+05
8e+05
7.6
7.5
7.4
7.3
7.2
7.1
7.0
ppm
cc: 0.803 / Japan / ID: 115050
arbitrary unit
0e+00
4e+05
2.5
2.4
2.3
2.2
2.1
2.0
ppm
cc: 0.803 / Japan / ID: 115050
arbitrary unit
0e+00
4e+05
7.6
7.5
7.4
7.3
7.2
7.1
7.0
ppm
cc: 0.829 / China / ID: 108118
arbitrary unit
0e+00
3e+05
6e+05
2.5
2.4
2.3
2.2
2.1
2.0
ppm
cc: 0.829 / China / ID: 108118
arbitrary unit
0e+00
3e+05
6e+05
7.6
7.5
7.4
7.3
7.2
7.1
7.0
ppm

Supplementary Figure 8F: Here, in the COMPASS approach, we applied a relatively conservative cross-correlation threshold (CC) of 0.75 for defining the acetaminophen metabolites Using this threshold, 19 spectra were missed in the COMPASS approach but were identified in the publication by Loo *et al* is shown below.

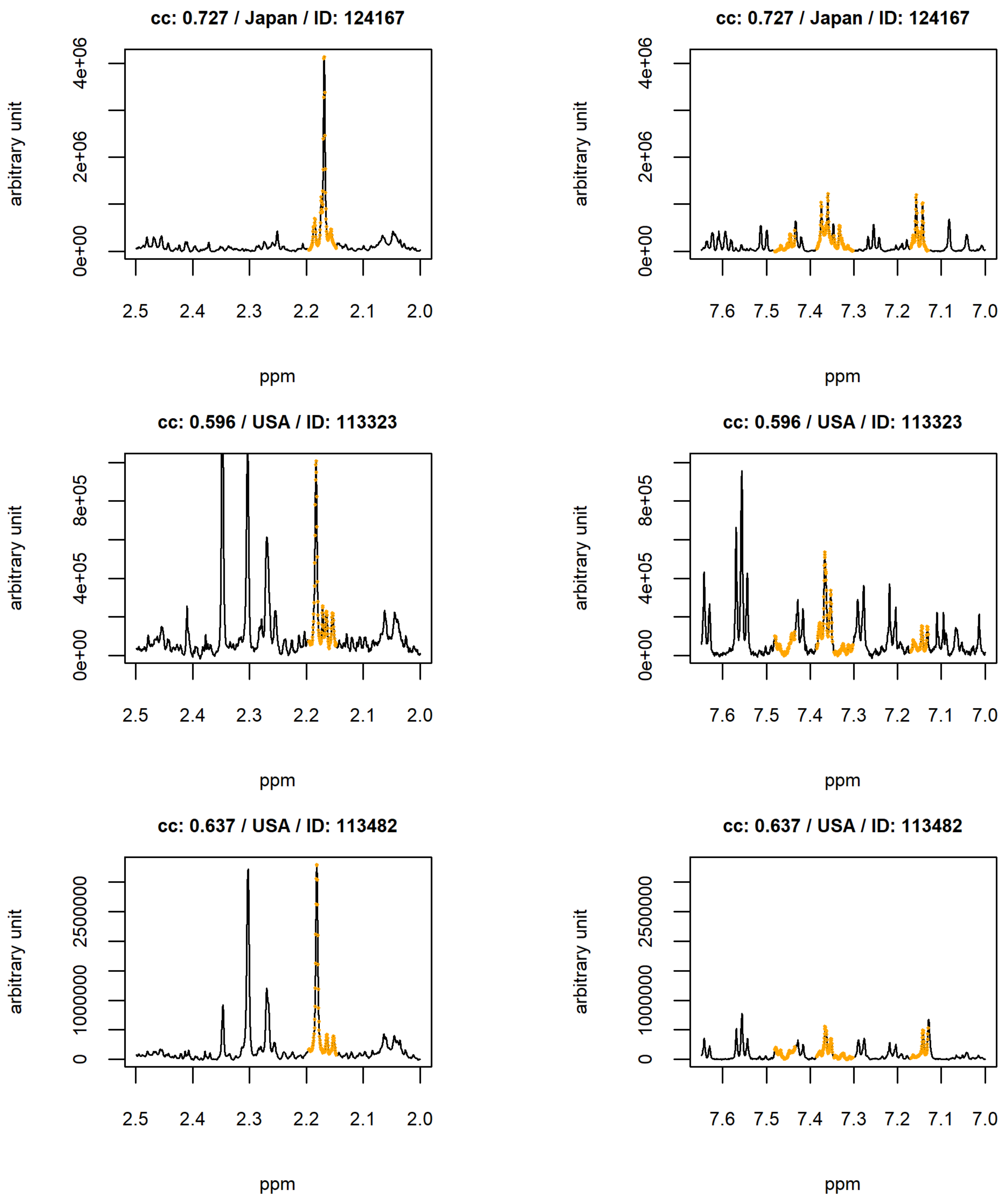

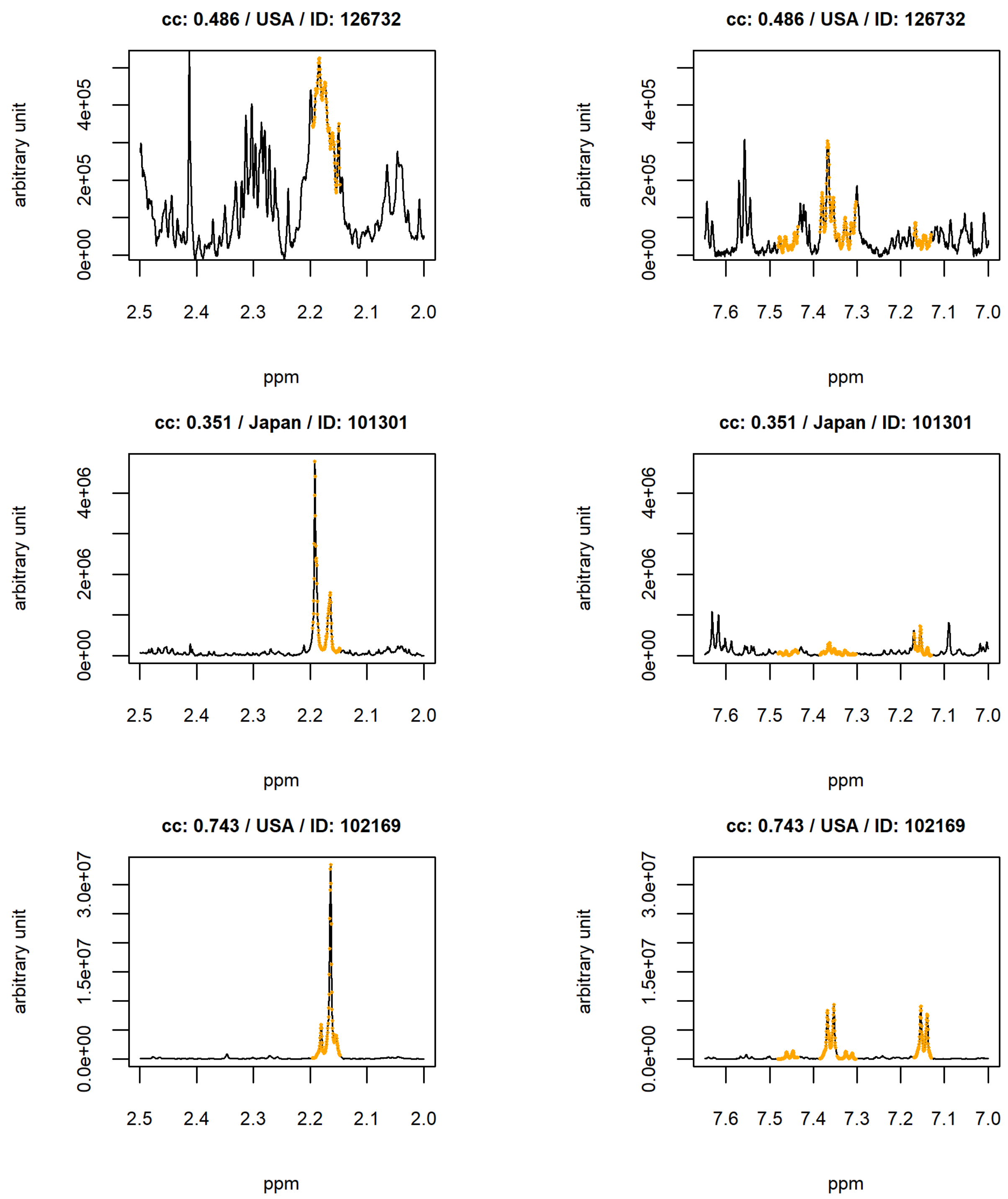
cc: 0.486 / USA / ID: 126732
cc: 0.486 / USA / ID: 126732
cc: 0.351 / Japan / ID: 101301
cc: 0.351 / Japan / ID: 101301
cc: 0.743 / USA / ID: 102169
cc: 0.743 / USA / ID: 102169
arbitrary unit
ppm
0e+00
2e+05
4e+05
2e+06
4e+06
0.0e+00
1.5e+07
3.0e+07
2.5
2.4
2.3
2.2
2.1
2.0
7.6
7.5
7.4
7.3
7.2
7.1
7.0

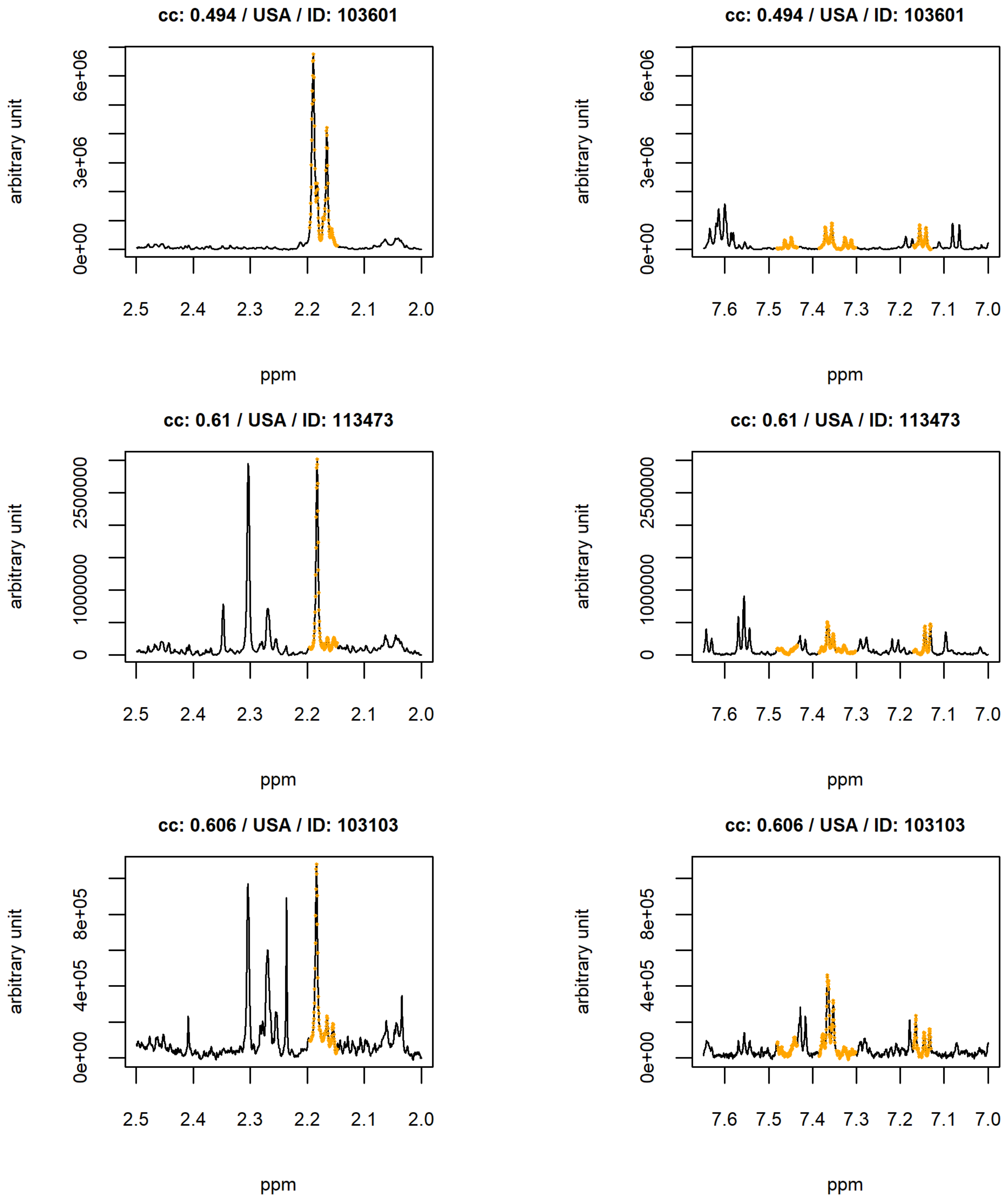
cc: 0.494 / USA / ID: 103601
arbitrary unit
0e+00
3e+06
6e+06
2.5
2.4
2.3
2.2
2.1
2.0
ppm
cc: 0.494 / USA / ID: 103601
arbitrary unit
0e+00
3e+06
6e+06
7.6
7.5
7.4
7.3
7.2
7.1
7.0
ppm
cc: 0.61 / USA / ID: 113473
arbitrary unit
0
1000000
2500000
2.5
2.4
2.3
2.2
2.1
2.0
ppm
cc: 0.61 / USA / ID: 113473
arbitrary unit
0
1000000
2500000
7.6
7.5
7.4
7.3
7.2
7.1
7.0
ppm
cc: 0.606 / USA / ID: 103103
arbitrary unit
0e+00
4e+05
8e+05
2.5
2.4
2.3
2.2
2.1
2.0
ppm
cc: 0.606 / USA / ID: 103103
arbitrary unit
0e+00
4e+05
8e+05
7.6
7.5
7.4
7.3
7.2
7.1
7.0
ppm

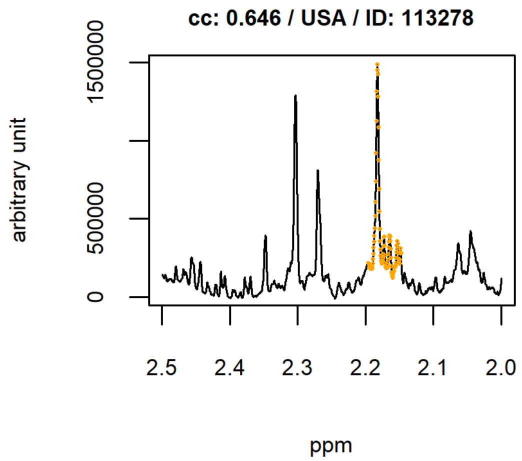
cc: 0.646 / USA / ID: 113278
arbitrary unit
0
500000
1500000
2.5
2.4
2.3
2.2
2.1
2.0
ppm

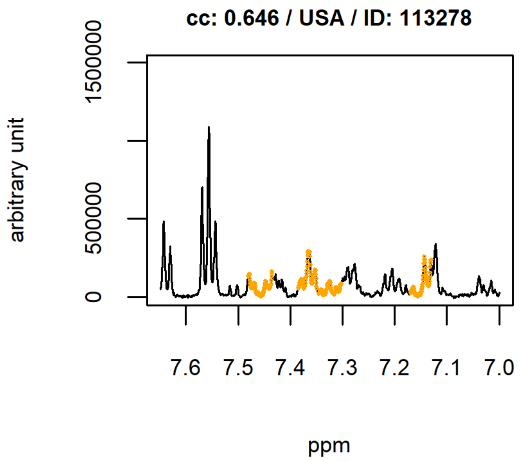
cc: 0.646 / USA / ID: 113278
arbitrary unit
0
500000
1500000
7.6
7.5
7.4
7.3
7.2
7.1
7.0
ppm

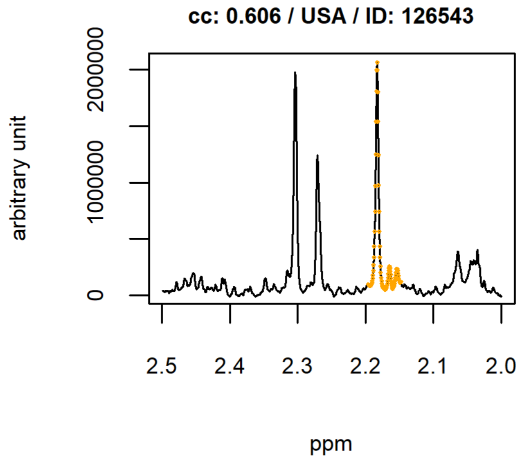
cc: 0.606 / USA / ID: 126543
arbitrary unit
0
1000000
2000000
2.5
2.4
2.3
2.2
2.1
2.0
ppm

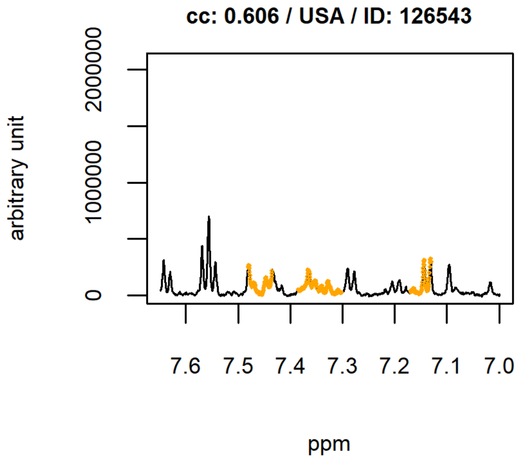
cc: 0.606 / USA / ID: 126543
arbitrary unit
0
1000000
2000000
7.6
7.5
7.4
7.3
7.2
7.1
7.0
ppm

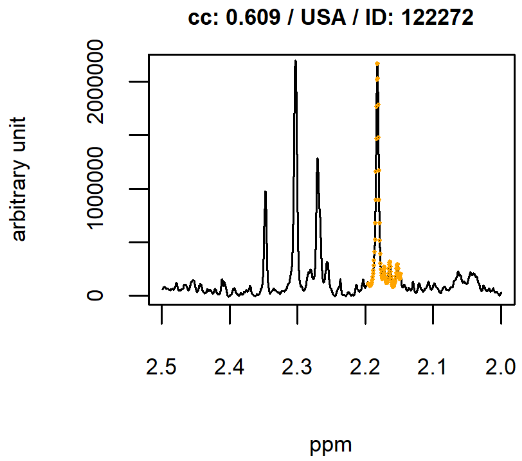
cc: 0.609 / USA / ID: 122272
arbitrary unit
0
1000000
2000000
2.5
2.4
2.3
2.2
2.1
2.0
ppm

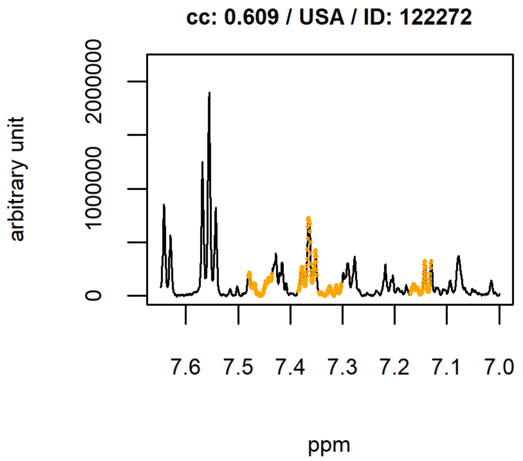
cc: 0.609 / USA / ID: 122272
arbitrary unit
0
1000000
2000000
7.6
7.5
7.4
7.3
7.2
7.1
7.0
ppm

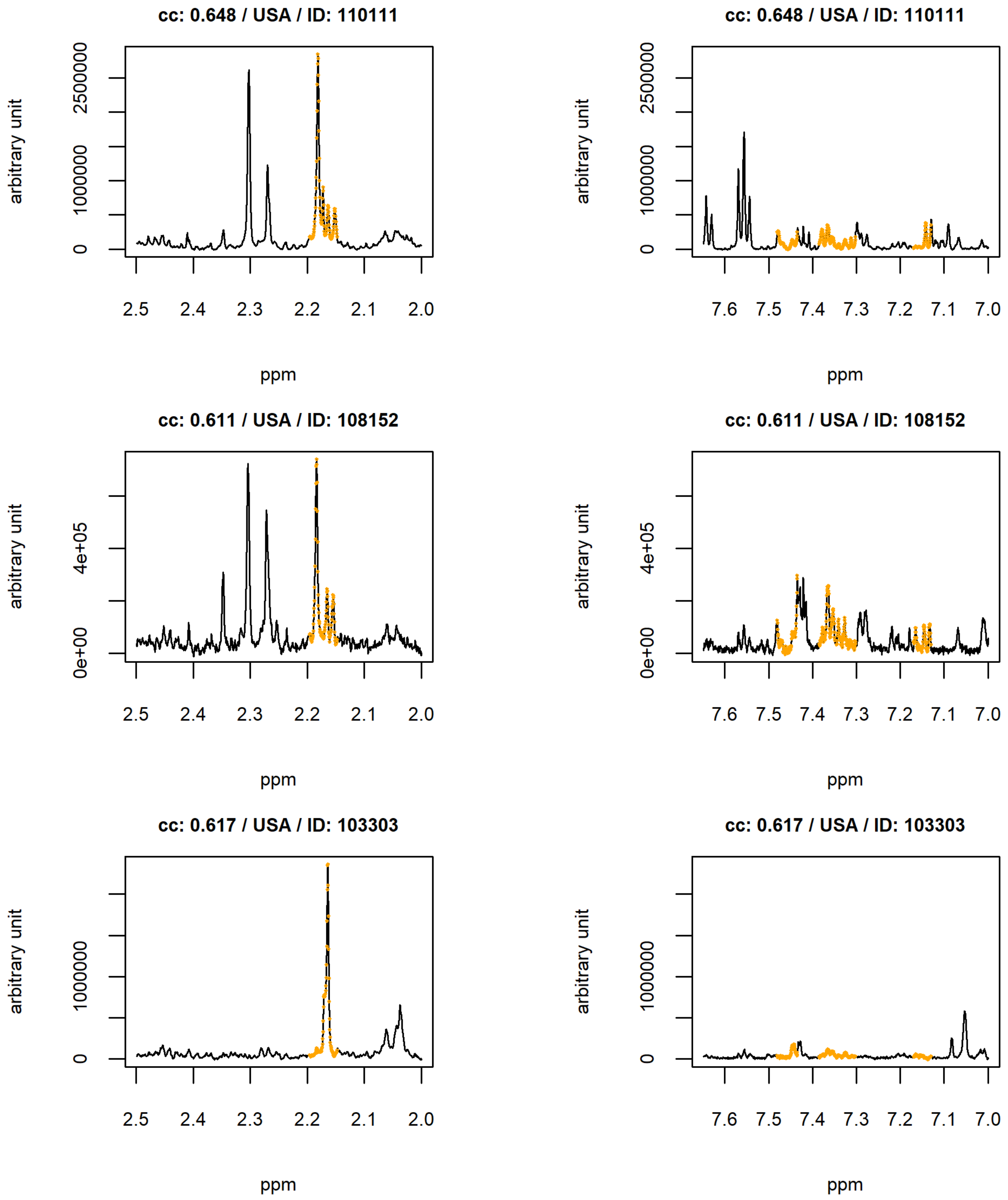
cc: 0.648 / USA / ID: 110111
cc: 0.648 / USA / ID: 110111
cc: 0.611 / USA / ID: 108152
cc: 0.611 / USA / ID: 108152
cc: 0.617 / USA / ID: 103303
cc: 0.617 / USA / ID: 103303
arbitrary unit
ppm
0
1000000
2500000
0e+00
4e+05
2.5
2.4
2.3
2.2
2.1
2.0
7.6
7.5
7.4
7.3
7.2
7.1
7.0

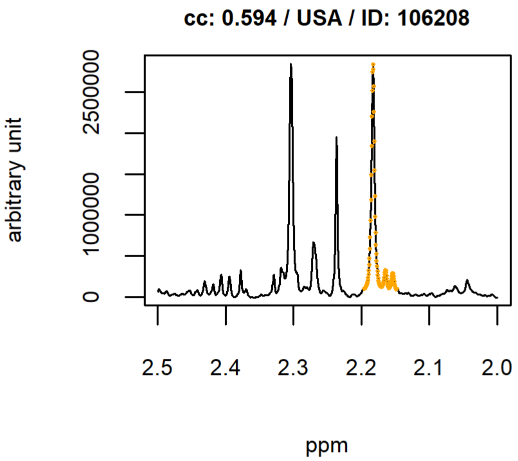
cc: 0.594 / USA / ID: 106208
arbitrary unit
0
1000000
2500000
2.5
2.4
2.3
2.2
2.1
2.0
ppm

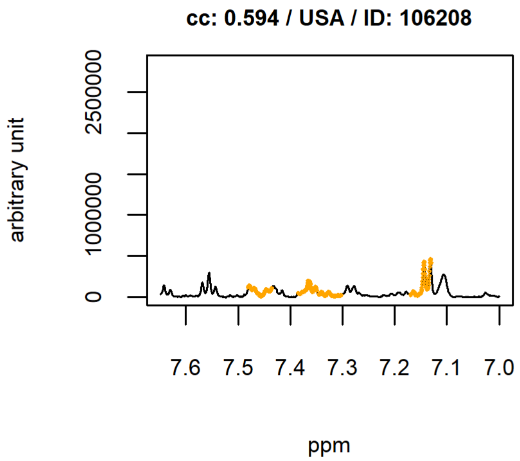
cc: 0.594 / USA / ID: 106208
arbitrary unit
0
1000000
2500000
7.6
7.5
7.4
7.3
7.2
7.1
7.0
ppm

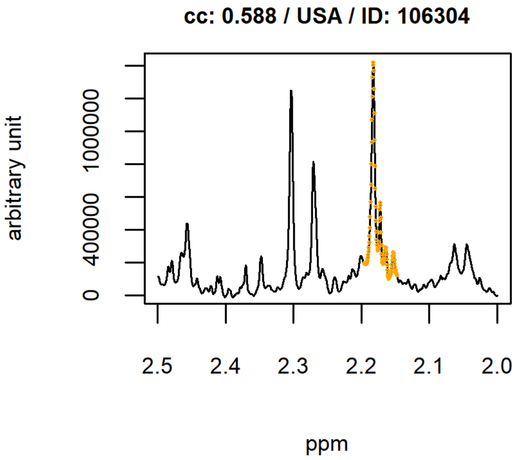
cc: 0.588 / USA / ID: 106304
arbitrary unit
0
400000
1000000
2.5
2.4
2.3
2.2
2.1
2.0
ppm

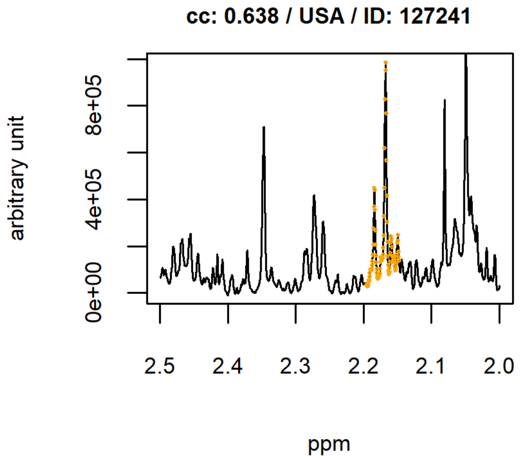
cc: 0.638 / USA / ID: 127241
arbitrary unit
0e+00
4e+05
8e+05
2.5
2.4
2.3
2.2
2.1
2.0
ppm

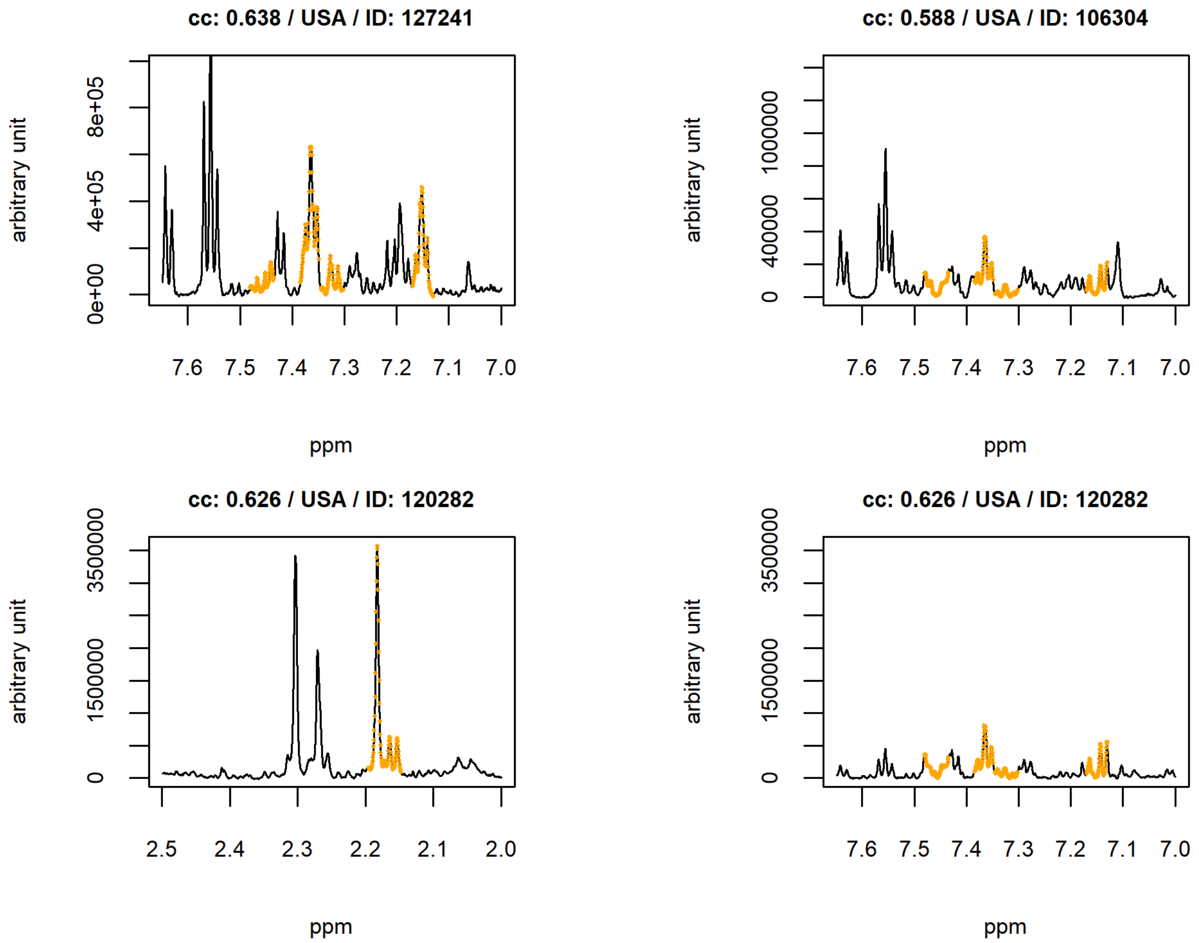

Supplementary Table 8A: Population statistics for acetaminophen based on COMPASS approach

```
##
## China Japan    UK   USA
##    36    24    65   234
```

Supplementary Table 8B: Population statistics for acetaminophen based on previous orthogonal PLS-DA prediction method by Loo *et al* 2012, the number of spectra containing acetaminophen metabolites in the urine and by country

```
##
## China Japan    UK   USA
##    23    20    61   220
```
