# Supplementary material for: Strategy for improved characterization of human metabolic phenotypes using a COmbined Multi-block Principal components Analysis with Statistical Spectroscopy (COMPASS)
Source: Bioinformatics. 2020 Jul 21;36(21):5229–36. doi: 10.1093/bioinformatics/btaa649 (PMC7850059; doi:10.1093/bioinformatics/btaa649)
Supplement: btaa649_Supplementary_Data [file btaa649_supplementary_data.zip › Supp 9_Glucose.pdf]

Supplementary Material 9: Typical output for COMPASS approach as illustrated using glucose

Supplementary Figure 9A: Robust reference pattern of glucose as identified by STOCSY.

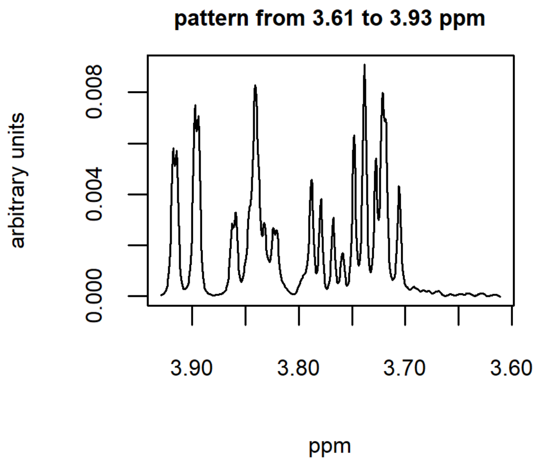

Supplementary Figure 9B: Distribution of cross-correlation using robust reference pattern of glucose as shown in Supplementary Figure 9A and color coded to countries: China (red), Japan (turquoise), UK (blue), and USA (grey).

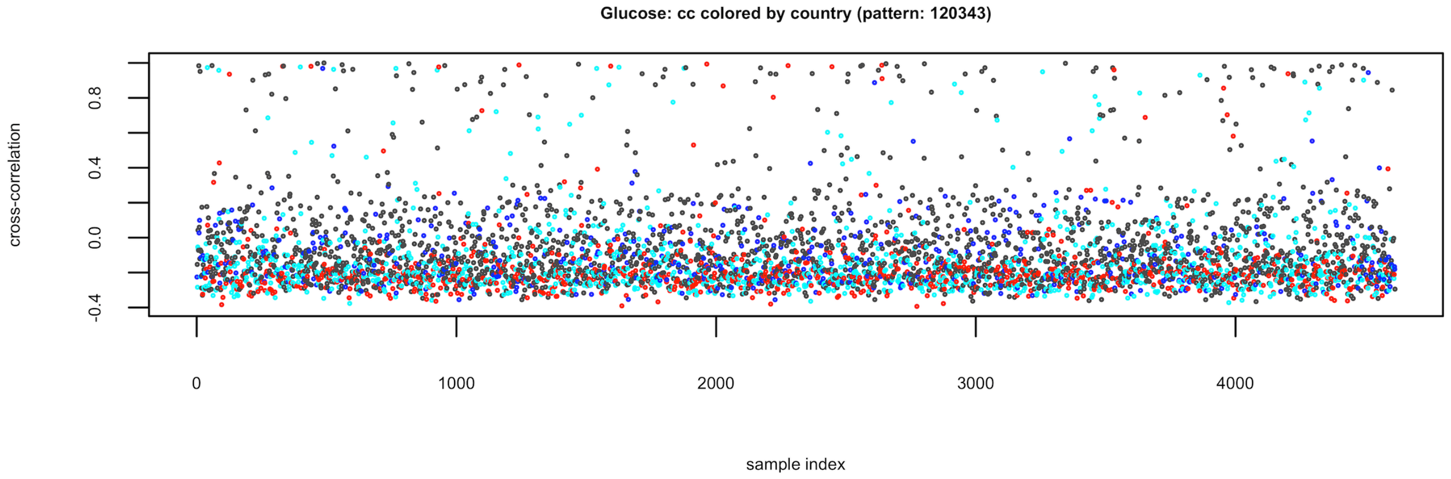

Supplementary Figure 9C: NMR spectra in the dataset showing glucose pattern with high cross correlation threshold (CC) value > 0.9 (in green), intermediate CC between 0.5 to 0.9 (in amber) and low threshold showing no feature at CC < 0.5 (in red). We have presented 6 randomly selected spectra in each category. Note, users may choose to output more spectra within the COMPASS framework.

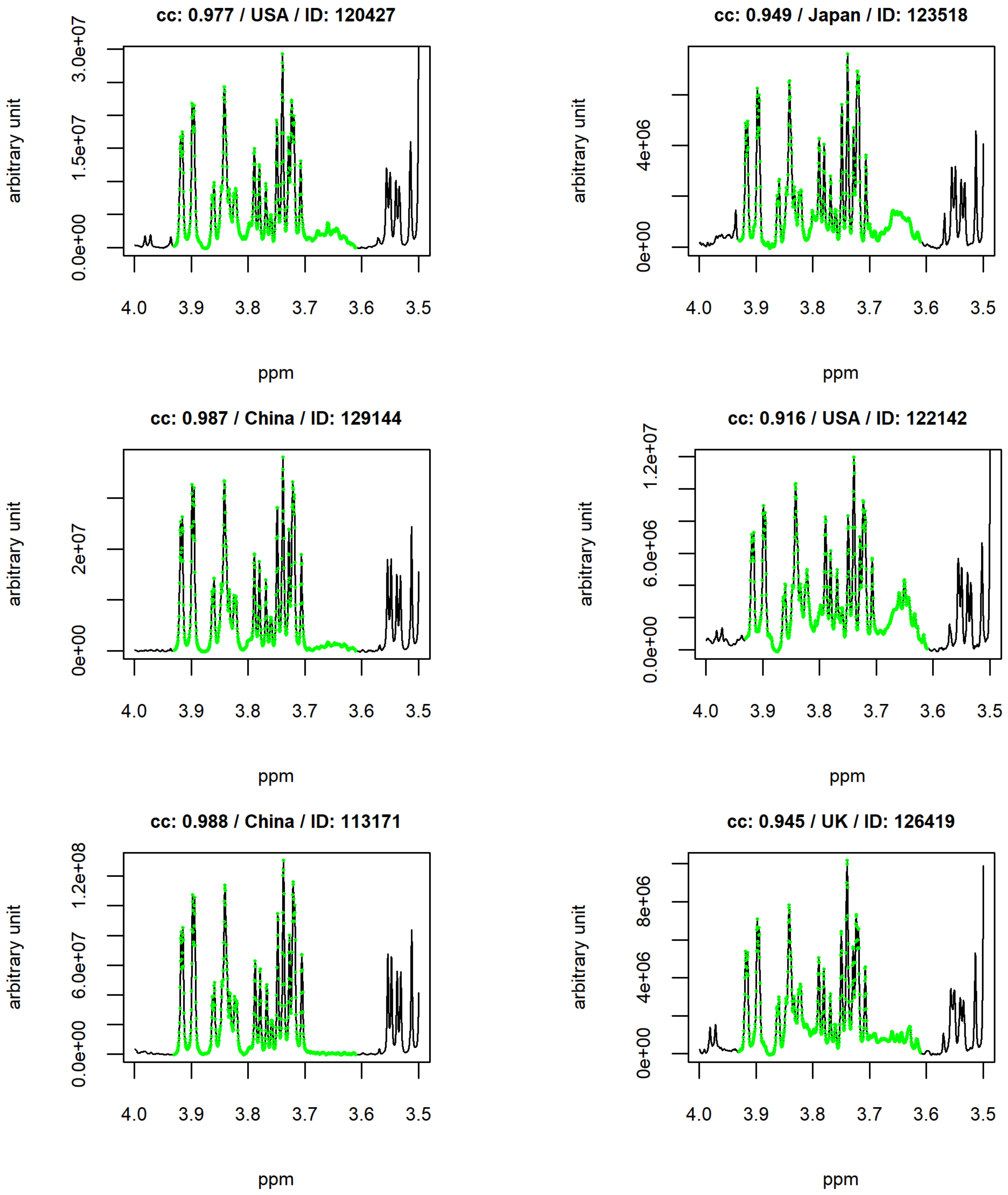

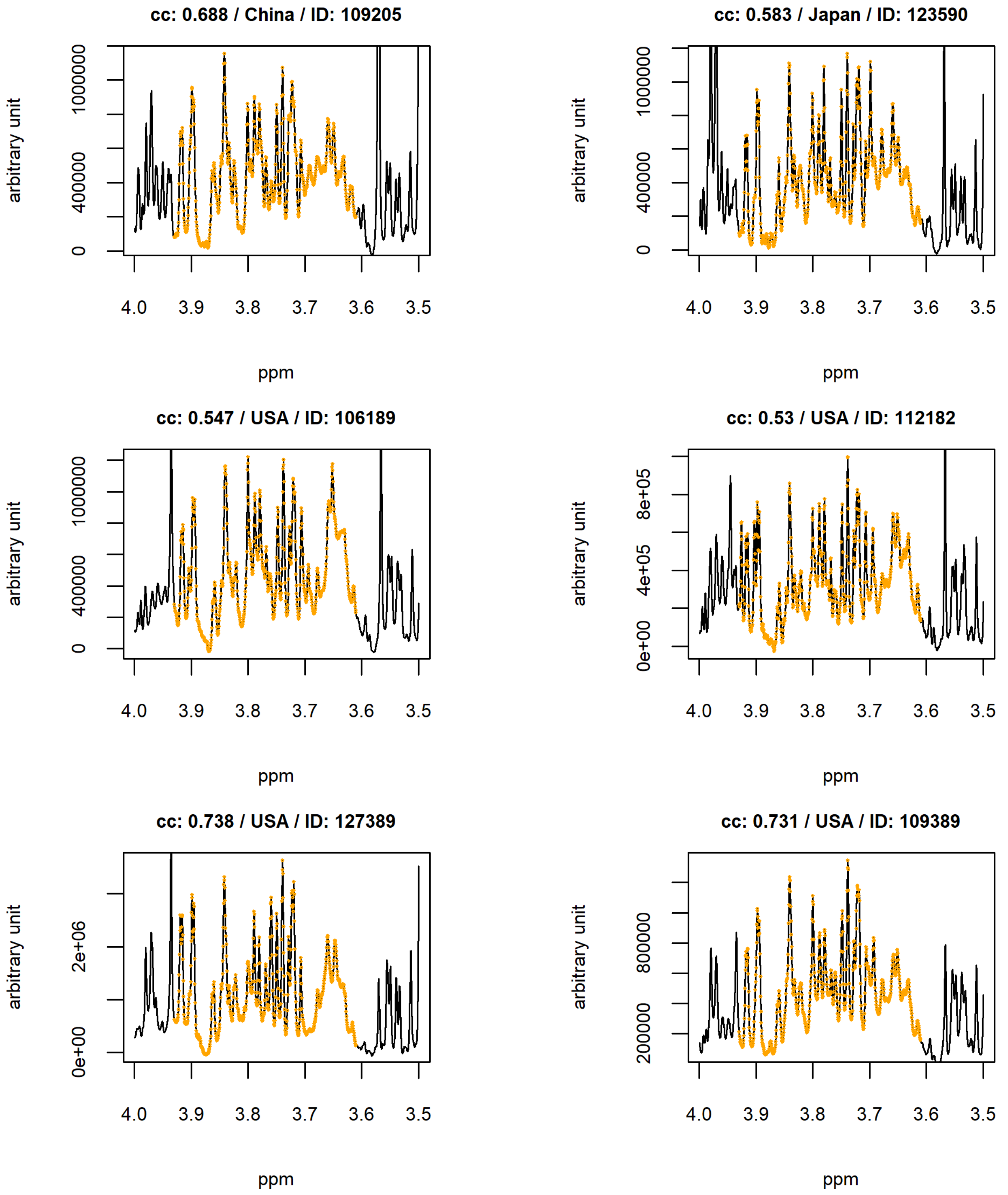
cc: 0.688 / China / ID: 109205
cc: 0.583 / Japan / ID: 123590
cc: 0.547 / USA / ID: 106189
cc: 0.53 / USA / ID: 112182
cc: 0.738 / USA / ID: 127389
cc: 0.731 / USA / ID: 109389
arbitrary unit
ppm
4.0
3.9
3.8
3.7
3.6
3.5
0
400000
1000000
0e+00
4e+05
8e+05
2e+06
200000
800000

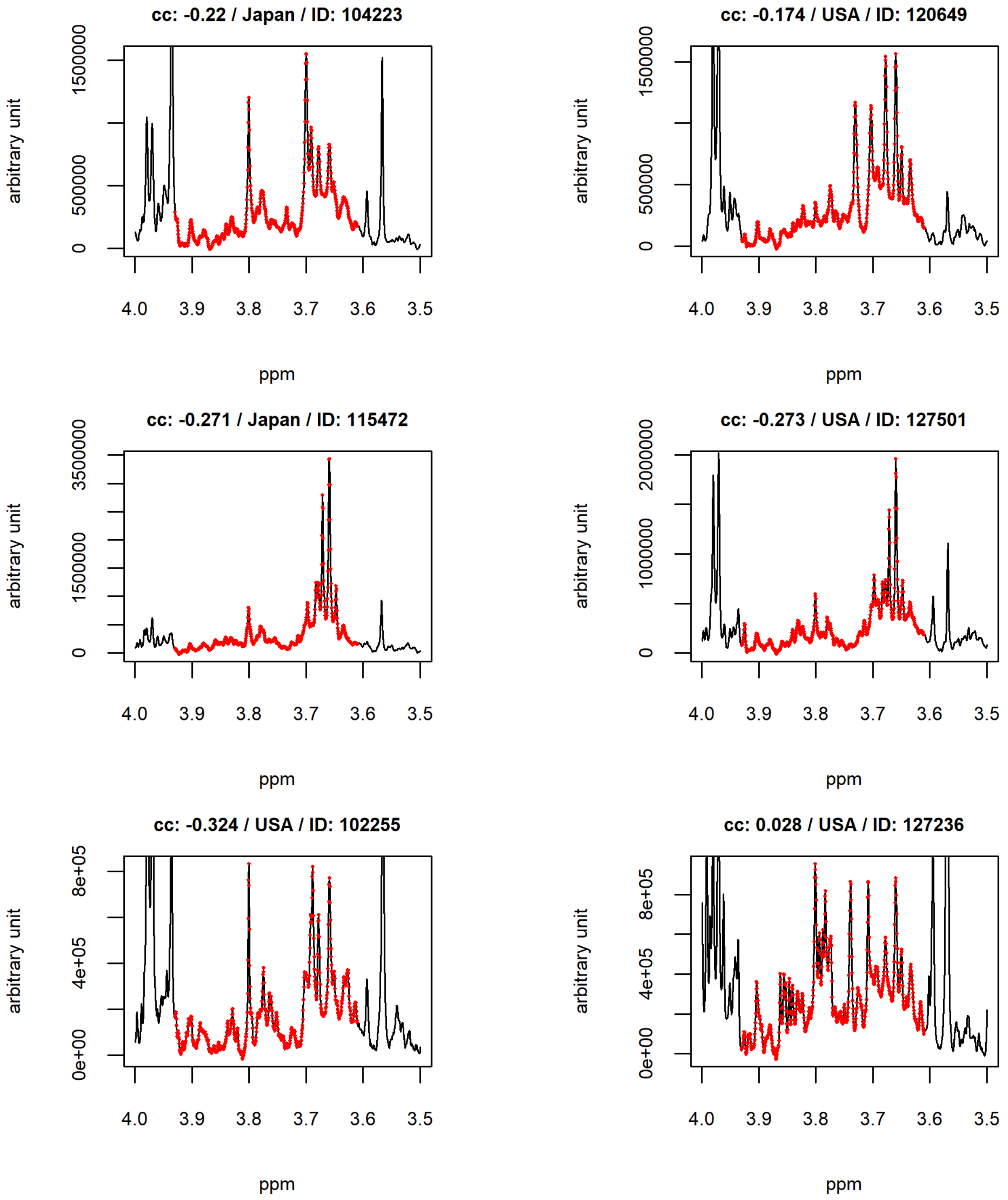
cc: -0.22 / Japan / ID: 104223
arbitrary unit
0
500000
1500000
4.0
3.9
3.8
3.7
3.6
3.5
ppm
cc: -0.174 / USA / ID: 120649
arbitrary unit
0
500000
1500000
4.0
3.9
3.8
3.7
3.6
3.5
ppm
cc: -0.271 / Japan / ID: 115472
arbitrary unit
0
1500000
3500000
4.0
3.9
3.8
3.7
3.6
3.5
ppm
cc: -0.273 / USA / ID: 127501
arbitrary unit
0
1000000
2000000
4.0
3.9
3.8
3.7
3.6
3.5
ppm
cc: -0.324 / USA / ID: 102255
arbitrary unit
0e+00
4e+05
8e+05
4.0
3.9
3.8
3.7
3.6
3.5
ppm
cc: 0.028 / USA / ID: 127236
arbitrary unit
0e+00
4e+05
8e+05
4.0
3.9
3.8
3.7
3.6
3.5
ppm

Supplementary Table 9: Population statistics for glucose using COMPASS approach

Percentage of samples with Glucose by country

```
##
## China Japan    UK   USA
##   2.5   3.3   1.4   5.4
```

Total number of samples with Glucose by country

```
##
## China Japan    UK   USA
##    21    38     7   117
```

Total number of samples with Glucose

```
## [1] 183
```
